# Cell-free DNA methylation markers for differential diagnosis of hepatocellular carcinoma

**DOI:** 10.1186/s12916-021-02201-3

**Published:** 2022-01-14

**Authors:** Biyuan Luo, Fang Ma, Hao Liu, Jixiong Hu, Le Rao, Chun Liu, Yongfang Jiang, Shuyu Kuangzeng, Xuan Lin, Chenyang Wang, Yiyu Lei, Zhongzhou Si, Guangshun Chen, Ning Zhou, Chengbai Liang, Fangqing Jiang, Fenge Liu, Weidong Dai, Wei Liu, Yawen Gao, Zhihong Li, Xi Li, Guangyu Zhou, Bingsi Li, Zhihong Zhang, Weiqi Nian, Lihua Luo, Xianling Liu

**Affiliations:** 1 Department of Oncology, The Second XiangYa Hospital of Central South University, 139 Renmin Middle Road, Changsha, 410011 Hunan Province China; 2 Burning Rock Biotech, Guangzhou, 510300 Guangdong China; 3 Department of General Surgery, The Second XiangYa Hospital of Central South University, 139 Renmin Middle Road, Changsha, 410011 Hunan Province China; 4 Department of Hepatopancreatobiliary Surgery, The Second Xiangya Hospital, Central South University, 139 Renmin Middle Road, Changsha, 410011 Hunan Province China; 5 Department of Infectious Disease, The Second Xiangya Hospital, Central South University, 139 Renmin Middle Road, Changsha, 410011 Hunan Province China; 6 Department of Oncology, 331 Hospital of Zhuzhou, Zhuzhou, 412002 Hunan Province China; 7 Center of Organ Transplantation, The Second Xiangya Hospital of Central South University, 139 Renmin Middle Road, Changsha, 410011 Hunan Province China; 8 Department of Hepatobiliary Surgery, Hunan Provincial Hospital, Hunan Normal University, No. 61 Jiafang West Road, Changsha, Hunan Province China; 9 Department of Gastroenterology, The Second Xiangya Hospital of Central South University, 139 Renmin Middle Road, Changsha, 410011 Hunan Province China; 10 Department of Infectious Disease, The First Hospital of Changsha, No. 311, Yingpan Road, Changsha, 410005 Hunan Province China; 11 Department of Orthopedics, The Second Xiangya Hospital, Central South University, Changsha, 410011 China; 12 Hunan Key Laboratory of Tumor Models and Individualized Medicine, The Second Xiangya Hospital, Changsha, 410011 China; 13 Chongqing University Cancer Hospital, No.181, Hangyu Road, Shapingba District, Chongqing, China; 14 Department of Oncology, Central Hospital of Enshi Autonomous Prefecture, Enshi Clinical College of Wuhan University, Enshi, 445000 Hubei Province People’s Republic of China

**Keywords:** DNA methylation, cfDNA, Hepatocellular carcinoma, Early detection of cancer, Liver cirrhosis

## Abstract

**Background:**

Aberrant DNA methylation may offer opportunities in revolutionizing cancer screening and diagnosis. We sought to identify a non-invasive DNA methylation-based screening approach using cell-free DNA (cfDNA) for early detection of hepatocellular carcinoma (HCC).

**Methods:**

Differentially, DNA methylation blocks were determined by comparing methylation profiles of biopsy-proven HCC, liver cirrhosis, and normal tissue samples with high throughput DNA bisulfite sequencing. A multi-layer HCC screening model was subsequently constructed based on tissue-derived differentially methylated blocks (DMBs). This model was tested in a cohort consisting of 120 HCC, 92 liver cirrhotic, and 290 healthy plasma samples including 65 hepatitis B surface antigen-seropositive (HBsAg+) samples, independently validated in a cohort consisting of 67 HCC, 111 liver cirrhotic, and 242 healthy plasma samples including 56 HBsAg+ samples.

**Results:**

Based on methylation profiling of tissue samples, 2321 DMBs were identified, which were subsequently used to construct a cfDNA-based HCC screening model, achieved a sensitivity of 86% and specificity of 98% in the training cohort and a sensitivity of 84% and specificity of 96% in the independent validation cohort. This model obtained a sensitivity of 76% in 37 early-stage HCC (Barcelona clinical liver cancer [BCLC] stage 0-A) patients. The screening model can effectively discriminate HCC patients from non-HCC controls, including liver cirrhotic patients, asymptomatic HBsAg+ and healthy individuals, achieving an AUC of 0.957(95% CI 0.939–0.975), whereas serum α-fetoprotein (AFP) only achieved an AUC of 0.803 (95% CI 0.758–0.847). Besides detecting patients with early-stage HCC from non-HCC controls, this model showed high capacity for distinguishing early-stage HCC from a high risk population (AUC=0.934; 95% CI 0.905–0.963), also significantly outperforming AFP. Furthermore, our model also showed superior performance in distinguishing HCC with normal AFP (< 20ng ml^−1^) from high risk population (AUC=0.93; 95% CI 0.892–0.969).

**Conclusions:**

We have developed a sensitive blood-based non-invasive HCC screening model which can effectively distinguish early-stage HCC patients from high risk population and demonstrated its performance through an independent validation cohort.

**Trial registration:**

The study was approved by the ethic committee of The Second Xiangya Hospital of Central South University (KYLL2018072) and Chongqing University Cancer Hospital (2019167). The study is registered at ClinicalTrials.gov(#NCT04383353).

**Supplementary Information:**

The online version contains supplementary material available at 10.1186/s12916-021-02201-3.

## Background

Hepatocellular carcinoma (HCC), the most prevalent form of liver cancer, is the 3rd leading cause of cancer-related deaths worldwide [1]. The majority of HCC cases develop progressively from chronic liver disease, primarily due to hepatitis B virus/hepatitis C virus (HBV/HCV) infection, or obesity-driven non-alcoholic fatty liver disease (NAFLD), are usually associated with advanced fibrosis or liver cirrhosis (LC) [2, 3]. HCC is a cancer where early detection would make a significant difference. Early-stage patients have much-improved prognosis compared to advanced stage patients, due to the relative efficacy of curative treatments (surgical resection, transplantation, or radiofrequency ablation) compared with systemic therapy [4]. Currently, HCC routine screening (every 6 months) in high risk population primarily relies on the detection of serum protein marker alpha-fetoprotein (AFP) and ultrasound imaging. Due to the lack of adequate specificity and sensitivity, AFP is challenged in recent studies, and no more recommended by the European Association for the Study of the Liver (EASL) and the American Association for the Study of Liver Diseases (AASLD) [5–7]. Ultrasound imaging is relatively inexpensive and a less demanding procedure for screening, but the sensitivity of ultrasound alone in small nodules (<2 cm) is only 21% [8]. Magnetic resonance imaging (MRI)/ computer tomography (CT) scan can exceed a sensitivity of 50% in early-stage subjects, but this procedure is typically reserved for those at risk since it is expensive and uncomfortable [8]. Other blood-based protein biomarkers such as des-γ-carboxyprothrombin (DCP), glypican-3 (GP3), and Golgi protein 73 (GP73) are not recommended in clinic [9–12]. Currently, most HCC cases are detected on the basis of clinical symptoms at advanced stage, rather than by high-quality screening techniques. The development of an earlier and more accurate screening assay remains an urgent unmet clinical need.

The utilization of cancer-linked genomic and epigenomic alterations for diagnosis, prognosis, and personalized medicine is becoming increasingly popular. Liquid biopsy, assessing circulating tumor DNA (ctDNA) released from apoptotic or necrotic tumor cells, can be used to interrogate the genomic and epigenomic profiles of a tumor [6]. Many studies have shown the promising results in ctDNA-based early cancer detection and highlighted its potentials in revolutionizing cancer screening and diagnosis [13, 14]. Among various tumor types studied, screening for HCC had achived the highest sensitivity, possibly due to the abundant blood supply in the liver [15]. Of all mechanisms for epigenetic alterations, DNA methylation alteration is the most common type. Comparing with genomic alterations, utilizing DNA methylation as a screening approach offers several advantages: [1] aberrant DNA methylation occurs when a methyl group (CH_3_) is added to a cytosine base in a cytosine–phosphate–guanine (CpG) dinucleotide, controlling gene transcription and expression, suggesting that altered DNA methylation patterns could be one of the first detectable neoplastic changes thus reflects the early changes in tumors [16, 17] [2]. Methylation alterations are frequently found in specific genomic regions such as CpG islands, which provides an opportunity to analyze multiple altered sites within each targeted region and tremendous number of targeted regions by targeted sequencing [17, 18]. At present, studies on utilization of methylation alterations were often conducted in the advanced HCC populations and healthy individuals as control, limiting their widespread application as the routine screening tool in the high-risk population including LC patients and hepatitis B surface antigen-seropositive (HBsAg+) individuals [19, 20]. The very few studies that included LC patients did not cover the full spectrum of cirrhosis (various causes and states). The performance of assays conducted in such population would be compromised in high-risk population due to the co-existence of inflammation, cirrhosis, and/or precancerous lesions. Therefore, it is necessary to profile healthy individuals, LC, and HCC patients in parallel to precisely identify early-stage HCC cases in high-risk population.

To overcome these problems, we developed and validated an HCC screening model based on cfDNA methylation profiles to effectively distinguish patients with HCC from the high risk population with chronic hepatitis B (CHB) or LC, as well as from the non-HCC individuals. Importantly, we compared the performance of our HCC screening model with AFP in distinguishing HCC patients who were AFP-normal and early-stage HCC patients (Barcelona Clinic Liver Cancer [BCLC] stage 0-A) from the high-risk population. We also investigated whether clinical parameters, including but not limited to aspartate transaminase (AST), alanine transaminase (ALT), and AFP values, would affect the performance of the HCC screening model. Here, we reported a multi-layer HCC screening model based on cfDNA methylation profiles and domenstrated it could be a reliable approach in the early dection of HCC in clincal practice.

## Methods

### Study design and participants

The aims of this study are [1] marker identification (from tissue samples) and [2] HCC screening model construction and validation (both from plasma samples). This study involved 187 HCC participants, and 735 participants without HCC (203 LC patients and 532 healthy individuals). All the participants were enrolled from December 2017 to June 2019, collected from 3 medical centers in China (The Second Xiangya Hospital of Central South University [*n*=502, the training cohort], Hunan People’s Hospital and Chongqing University Cancer Hospital [*N*=420, the validation cohort]). Inclusion criteria included [1] ≥ 18 years of age and [2] must be treatment (surgery or chemotherapy) -naïve. Patients with intrahepatic cholangiocarcinoma including combined hepatocellular-cholangiocarcinoma or other malignancies were excluded. Healthy individuals were defined as having no clinical symptoms of liver disease nor history of cancer at the time of enrollment. HCC and LC tissue samples were either obtained at the time of segmental surgical resection or at biopsy. Normal liver tissues were obtained from liver donors who died of non-liver related causes. We conducted the pathology review on all of the tissue samples. All stages of HCC patients were included with a bias toward BCLC stage B or lower. HCC cases and healthy controls are age and sex balanced. Tissue samples were used to screen differentially DNA methylation blocks which can be used for healthy, LC and HCC plasma sample classification. A multi-layer HCC screening model was subsequently constructed based on tissue-derived differentially methylated makers and further validated in an independent cohort.

### Power analysis

The study statistical plan incorporated group sizes of 144 HCC patients, 144 LC patients, and 144 healthy controls. It was sufficient to verify that our assay had an expected sensitivity and specificity both at 75% with a power of 1−β = 90% and a significance level of α = 0.05. Additional HCC cases, LC, and healthy controls were incorporated into the study due to their availability.

### DNA extraction from tissues and plasma

DNA from tumor, LC, and healthy liver tissue were extracted using the QIAamp DNA FFPE Tissue Kit (Qiagen, Valencia, CA, USA). The presence of tumor cells in HCC tissue samples and the abscence of tumor cells in non-HCC tissue samples were confirmed by histopathological assessment prior to DNA extraction. Circulating cfDNA was recovered from 4 to 5 ml of plasma using the QIAamp Circulating Nucleic Acid kit (Qiagen, Valencia, CA, USA). DNA was quantified with the Qubit 2.0 fluorimeter (ThermoFisher Scientific, Waltham, MA, USA). The distribution of the amount of input is shown in Additional file 3: Fig. S1. Extracted tissue DNA and cfDNA were stored in IDTE buffer at −20°C and −80°C, respectively.

### Marker discovery and validation

We identified the differential methylation sites using Infinium HumanMethylation450K array data downloaded from The Cancer Genome Atlas (TCGA) database with the Benjamini–Hochberrg-corrected false discovery rate (FDR)<0.05. We used data from 656 normal WBC samples in the Gene Expression Omnibus (GEO) dataset to exclude hypermethylated CpG sites in haematopoietic lineage (>0.1). CpG sites on X or Y chromosomes were removed. We identified differentially methylated CpG sites. In addition, we included CpG sites that are associated with common cancers in previous studies. 85,250 CpG sites were identified in the marker discovery phase. The selected CpG sites were segregated into 8147 blocks (Additional file 1: Table S1) and later validated using data from tissue samples and plasma from healthy individuals [21].

### Targeted bisulfite sequencing

Fragmented tissue DNA (peak approximately 200bp) and cfDNA were subjected to bisulfite conversion using EZ-96 DNA methylation-lightening MagPrep (Zymo research, CA, USA). Briefly, purified DNA was treated with sodium bisulfite. Subsequently, the converted single-strand DNA molecules were ligated to a splinted adapter and amplified by an uracil-tolerating DNA polymerase to generate whole-genome BS-seq libraries. Custom-designed methylation profiling RNA baits were used for target enrichment which covers the 85,250 CpG sites and spans 1.16 mega base of the human genome. The target libraries were subsequently quantified by real-time PCR (Kapa Biosciences Wilmington, MA, USA) and sequenced on NovaSeq 6000 (Illumina, San Diego, CA, USA) with an average sequencing depth of 500X for tissue samples and 1000X for plasma samples. The total reads number for plasma is 49.24 million on average, given 2x150bp sequencing. The library preparation process includes five steps: DNA end-repair, Tail-and-Tag, single-tag DNA amplification, PCR amplification, and target enrichment.

### Methylation data processing

Raw sequencing data (.fastq) were first trimmed by Trimmomatic (v.0.36) and then aligned by BWA-meth (v.0.2.0) to the C to T- and G to A-transformed hg19 reference genome [22]. PCR duplicate reads were identified and removed by Picard tools (v.1.138). Paired reads were stitched together to represent the originating DNA fragments, and those with discordant pairing, or low mapping quality (MAPQ<60) were removed from further analyses.

### Model construction

A custom module was built to classify samples using two layers of models: (i) three linear kernel support vector machine (SVM) models: a malignant versus healthy model (MH model), a malignant versus benign model (MB model), and a benign versus healthy model (BH model). Each model searches for a hyperplane with maximal distances from both two pre-defined training classes. Like all linear classifiers, the decision function is presented as $f\left(\mathrm{x}\right)= wT\ \mathrm{x}+b$, where *w* = [ *w*_1_, *w*_2_, …, *w*_*k*_ ]*T* is the weight vector and *b* represents the distance of the hyperplane from the origin. (ii) A multinomial logistic regression model: for each sample, the output from the MH, MB, and BH models was fed into a multinomial logistic regression model to obtain a cancer/benign/healthy assignment as a final prediction. Both layers were trained by the stochastic gradient descent (SGD) algorithm, and the performance of the training set was assessed by iterated 5-fold cross-validation. During the independent validation phase, the model with locked parameters was applied directly to the blind samples and the clinical information was not released until all analyses were completed.

### Statistical analysis

Means and differences of the means with 95% confidence interval (CI) were calculated using the Wilson's score CI. A *p* value of <0.05 was considered statistically significant. Differences between the groups were calculated using the two-tailed Student’s *t* test, the Kruskal-Wallis test, or the Fisher’s exact test, where appropriate. All statistical analyses were performed with R (R version 3.4.0; R: The R-Project for Statistical Computing, Vienna, Austria) using default functions and packages “*FactoMineR*” (v2.4) and “*factoextra*” (v1.0.7). The differential methylation regions were called using the package “*limma*” (v2.0), and the cut-off was set as Benjamini–Hochberrg-corrected FDR <0.05. Linear models and empirical Bayes methods were used for assessing differential expression in microarray experiments. The first layer of HCC screening model was constructed by applying the package “*e1071*” (v1.7-9) using a linear kernel with C set as 1. The second layer of HCC screening model was trained with the package “*nnet”* (v7.3-16) using the single layer model.

### Ethics committee approval

The study was approved by the ethic committee of The Second Xiangya Hospital of Central South University (KYLL2018072) and Chongqing University Cancer Hospital (2019167). All collection and usage of human samples and clinical data were in accordance with the principles of the Declaration of Helsinki. Written informed contents were obtained from all participants for the use of their tissue or plasma samples.

## Results

### Patient characteristics

**Fig. 1:**
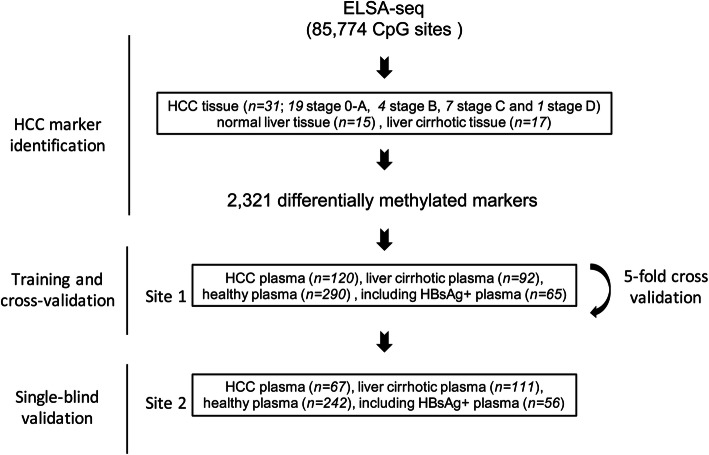
Workflow chart of data generation and diagnosis analysis. ELSA-seq panel 85,250 CpG sites were applied to a training cohort of 15 normal liver tissue, 17 liver cirrhotic tissue, 31 HCC tissue (19 stage 0-A, 4 stage B, 7 stage C, and 1 stage D) to identify a final selection of 2321 differentially methylated markers. These markers were applied to a training and cross-validation cohort and also a single-blind validation cohort.

Tissue samples, obtained from 31 treatment-naïve HCC patients with various stages, 17 LC patients and 15 liver donors were used for screening differentially DNA methylation blocks to classify HCC patients and non-HCC controls. The HCC screening model, comprising differentially DNA methylation markers derived from tissue samples, was constructed using plasma samples obtained from 120 patients with HCC, 92 LC patients and 290 healthy individuals, including 65 HBsAg+ individuals. This model was subsequently validated in an independent cohort consisting of 67 patients with HCC, 111 LC patients, and 242 healthy individuals, including 56 HBsAg+ individuals. The study design was depicted in Fig. 1. Detailed patient characteristics were summarized in Table 1.

**Table 1:** Patient characteristics of the study population

	HCC			Cirrhosis			Healthy Individuals		
	Training	Validation	***P*** value	Training	Validation	***P*** value	Training	Validation	***P*** value
Total, *n*	120	67		92	111		290	242	
Age, years									
Mean ± SD	53(24-83)	54(23-74)	0.599	48(26-81)	52(22-82)	0.005	50(21-89)	50(18-79)	0.633
AFP, ng/ml^#^									
Negative, ≤20 ng/ml	41	20	0.869	53	65	0.617	83	63	
Positive, >20 ng/ml	77	42		21	32		0	0	
UNK	2	5		18	14		207	176	
Gender									
Male	106	55	0.273	74	89	0.86	236	192	0.584
Female	14	12		17	22		54	50	
UNK				1					
HBV infection^*^									
No	11	9	0.461	22	9	0.003	60	54	0.896
Yes	105	57		67	98		65	56	
UNK	4	1		3	4		165	132	
Child-Pugh class									
A	102	54	0.491	14	39	0.004			
B	15	9		33	37				
C	1	2		44	35				
UNK	2	2		1					
BCLC stage									
0	7	3	0.698						
A	65	34							
B	18	8							
C	27	18							
D	3	4							

^#^AFP was not reported by some healthy individuals; ^*^HBV status was not reported by some healthy individuals

*AFP* alpha-fetorprotein, *BCLC* Barcelona Clinic Liver Cancer, *HBV* hepatitis B virus, *HCC* hepatocellular carcinoma, *UNK* unknown, *SD* standard deviation

### The identification of HCC markers from tissue samples

To identify markers for distinguishing HCC patients, DNA methylation profiles of tissue sampes were compared by performing capture-based targeted bisulfite sequencing. Collectively, 2321 differentially methylated markers, including 2293 tumor-specific and 279 tissue-specific markers were identified by comparing the methylation levels of tumor tissues to plasma from healthy individuals and normal liver tissue to plasma from healthy individuals, respectively. Among them, 251 markers were both tissue and tumor-specific. The performace of an unsupervised clustering based on 2321 markers in tissue samples achieved a sensitivity of 94% and a specificity of 100% with an area under curve (AUC) of 99.8% (95% CI 98.6–100%) (Additional file 3: Fig. S2A, Additional file 2: Table S2). Of 2293 tumor-specific markers, 2082 are hypermethylated and 211 are hypomethylated (Additional file 3: Fig. S2B); of 279 tissue-specific markers, 158 are hypermethylated and 121 are hypomethylated (Additional file 3: Fig. S2C). Based on GO and KEGG pathway analysis (Additional file 3: Fig. S3), we found that tumor-specific markers tends to enrich in cancerization-related functions while tissue-specific markers are more abundant in categories of development and differentiation.

### Circulating free DNA-based prediction for HCC and LC

We aimed to develop an HCC screening model based on methylation profiles obtained from cfDNA to distinguish patients with HCC from LC patients and healthy individuals. An unsupervised clustering was performed to visualize the methylation profiles of the plasma samples from training cohort for the tissue-derived 2321 markers (Additional file 3: Fig. S4). Although the methylation signals were less distinct in plasma samples due to the low tumor shedding in early-stage HCC, an increase in methylation intensity was observed from healthy individuals to HCC patients.

Firstly, three sub-models (MH, BH, and MB models) were trained based on the training data for the selected 2321 differentially methylated markers. The classification accuracies were highly consistent between the training and single-blind validation sets in 3 sub-models, confirming effective modeling and minimal overfitting. The MH model, distinguishing HCC samples from healthy controls, achieved a sensitivity of 84% and a specificity of 100% in the training cohort (AUC=0.992), and a sensitivity of 82% and a specificity of 100% in the validation cohort (AUC=0.984; Additional file 3: Fig. S5A-D). The BH model, distinguishing LC samples from healthy controls, had a sensitivity of 88% and specificity of 98% in the training cohort (AUC=0.983), and a sensitivity of 66% and a specificity of 99% in the validation cohort (AUC=0.933; Additional file 3: Fig. S6A-D). Tissue-specific markers made significant contributions to BH score (Additional file 3: Fig. S7) The MB model, distinguishing HCC samples from LC controls, had a sensitivity of 88% and a specificity of 90% (AUC=0.968) and yielded a sensitivity of 90% and a specificity 81% in validation cohort (AUC=0.943; Additional file 3: Fig. S8A-D). Subsequently, a multinomial logistic regression model was built up using the predictive values generated from three sub-models (MH, BH, and MB models) to achieve the screening results for unknown samples. Overall, the HCC screening model yielded a sensitivity of 86% and a specificity of 98% in the training data set (AUC=0.98, 95% CI 0.969–0.991), and a sensitivity of 84% and a specificity of 96% in the validation data (AUC=0.97, 95% CI 0.945–0.994; Table 2 & Additional file 3: Fig. S9). Sensitivity improved with advancing disease stage (Table 2). Sensitivity in the training set was 79% in stage 0-A (57/72), 94% in stage B (17/18), and 97% in stage C-D (29/30) patients. Sensitivity in the validation set was 76% in stage 0-A (28/37), 88% in stage B (7/8), and 95% in stage C-D (21/22) patients.

**Table 2:** Performance of tissue-derived markers in plasma samples (training and validation cohort)

***Training cohort***		Predicted				
	Total	Healthy	Cirrhosis	HCC	Sensitivity (%)	Specificity (%)
***Healthy***	290	284	3	3		98
***Cirrhosis***	92	14	72	6		93
***Healthy+Cirrhosis***	382	298	75	9		98
***0-A***	72	10	5	57	79	
***B***	18	0	1	17	94	
***C-D***	30	0	1	29	97	
***HCC***	120	10	7	103	86	
***Validation cohort***		**Predicted**				
	**Total**	**Healthy**	**Cirrhosis**	**HCC**	**Sensitivity (%)**	**Specificity (%)**
***Healthy***	242	241	0	1		100
***Cirrhosis***	111	36	62	13		88
***Healthy+Cirrhosis***	353	277	62	14		96
***0-A***	37	9	0	28	76	
***B***	8	1	0	7	88	
***C-D***	22	1	0	21	95	
***HCC***	67	11	0	56	84	

### Clinical significance of malignant score and benign score

**Fig. 2:**
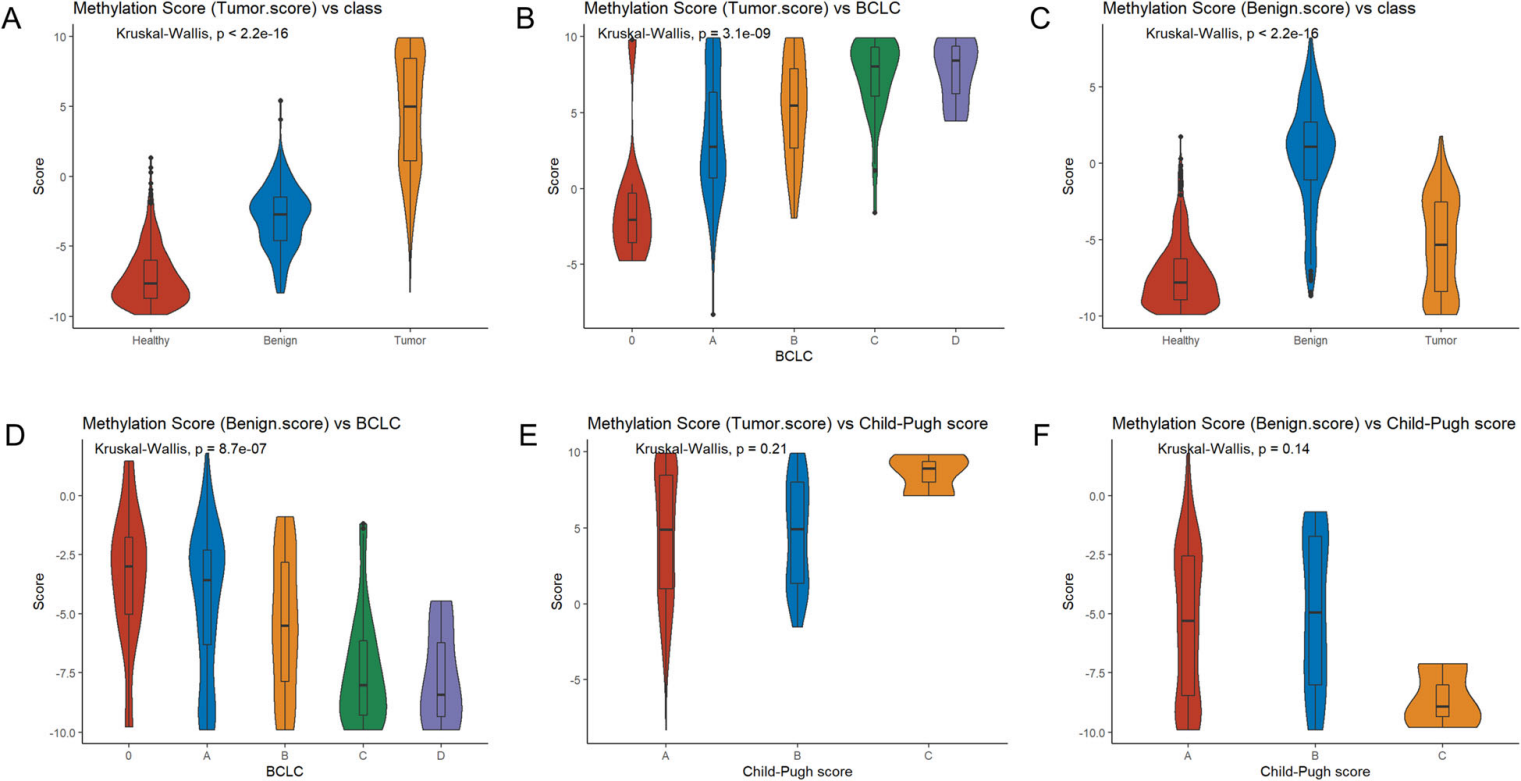
Clinical significance of malignant score and benign score. **A**, **C** The distribution of tumor score or benign score in HCC, LC patients, and healthy individuals. **B**, **D** The distribution of tumor score or benign score in BCLC (0-D) stage. **E**, **F** The distribution of tumor score or benign score in Child-Pugh score (**A**–**C**)

**Fig. 3:**
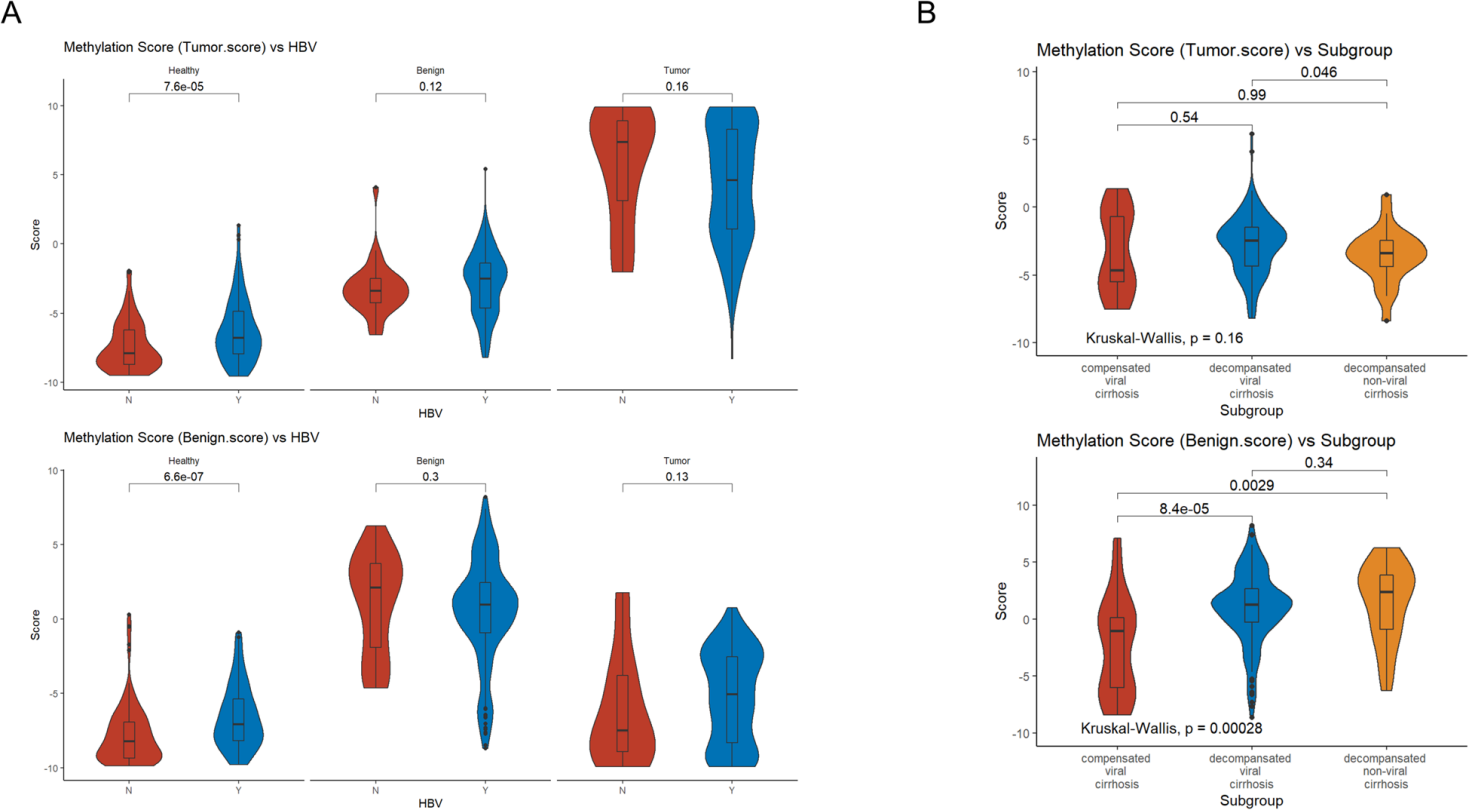
The correlation of tumor score and benign score with cause of cirrhosis (HBV or non-HBV) and the stage of cirrhosis (compensated vs decompensated). **A** HBV status does not affect tumor nor benign score for HCC diagnosis. **B** Patients’ cirrhosis stage affects benign score but not tumor score

To investigate the clinical significance of malignant and benign scores, which were the outputs of our HCC screening model, we correlated both scores with several clinical parameters, including BCLC stage, Child-Pugh (CP) score, and liver diseases with hepatitis B virus/hepatitis C virus (HBV/HCV) infection. The median malignant score of HCC, cirrhosis, and healthy controls were 5.4, −2.6, and −8.2, respectively (Fig. 2A). Our analysis showed that malignant score progressively increased from stage 0 to D (*p*<0.01) (Fig. 2B). Benign score, reflecting the properties of cirrhotic livers, is significantly higher in patients with cirrhosis (*p*<0.01) with a median benign score of 0.9. Healthy individuals and patients with HCC had a median benign score of −8.3 and −5.5, respectively (Fig. 2C). Both malignant score (*p*=0.21) and benign score (*p*=0.14) were comparable among patients with CP score A, B, or C, suggesting both scores were not affected by CP scores. In patients with HCC and non-HCC controls, HBV and HCV infectious background did not affect malignant nor benign score (Fig. 3A & Additional file 3: Fig. S10). Compensated viral cirrhosis, decompensated viral cirrhosis, and decompensated non-viral cirrhosis groups had comparable malignant scores (Kruskal-Wallis, *p*=0.16), further suggesting that malignant score primarily reflects the properties of tumor. Decompensated viral cirrhosis and decompensated non-viral cirrhosis had comparable benign scores (*p*=0.34; Fig. 3B). Compensated viral cirrhosis had a significantly lower benign score than decompensated viral cirrhosis (*p*<0.01) and decompensated non-viral cirrhosis (*p*=0.0029), suggesting that benign score primarily reflects the properties of cirrhosis.

### Performance of the HCC screening model and AFP

**Fig. 4:**
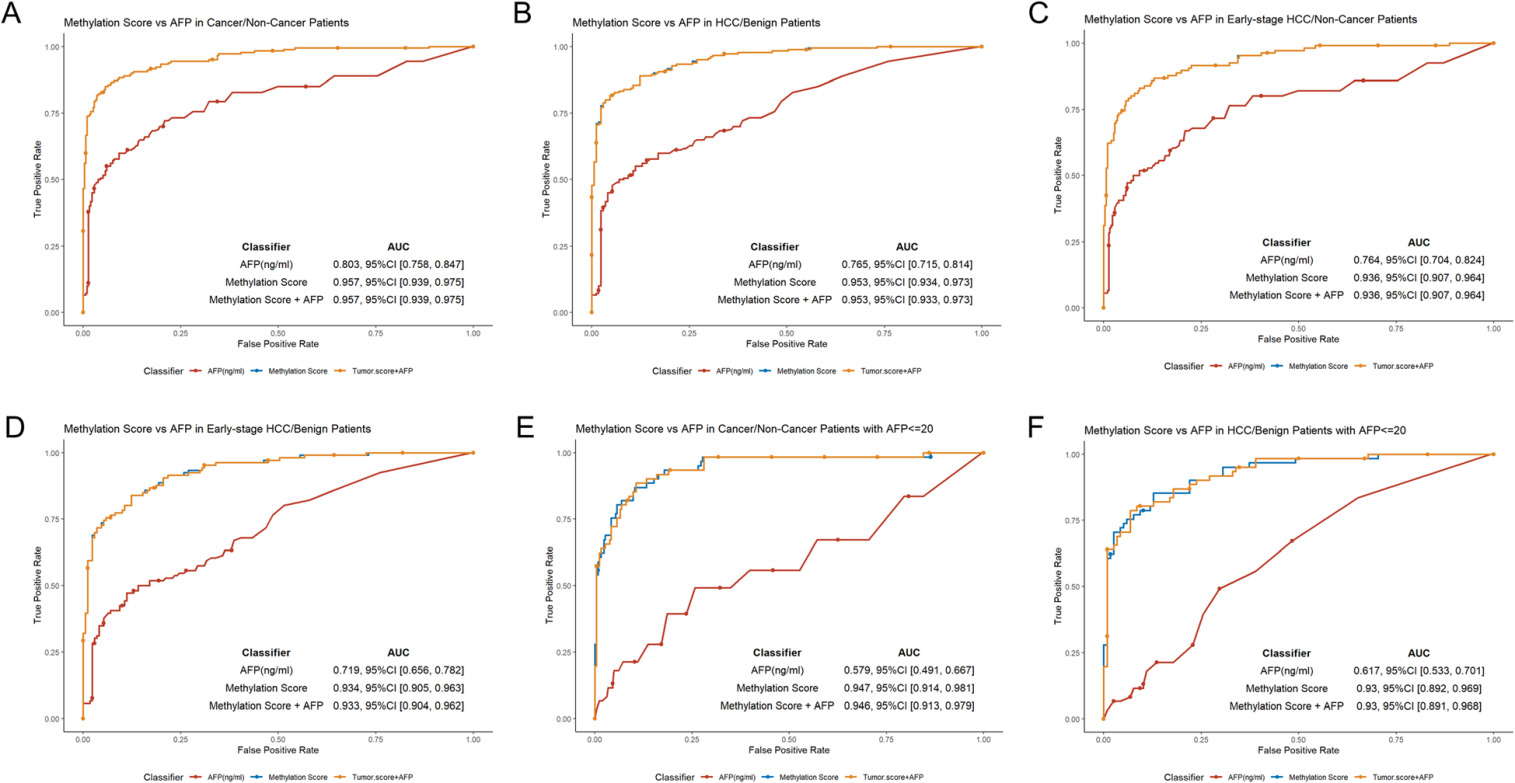
HCC detection accuracy of methylation score compared with serum AFP tests. The performance of methylation score and AFP, in differentiating HCC patients from non-cancerous individuals (**A**); in differentiating HCC patients from LC patients (**B**); in distinguishing early-stage HCC patients from non-cancerous individuals (**C**); in distinguishing early-stage HCC patients from LC patients (**D**); in differentiating HCC patients with an AFP level ≤ 20 ng/mL from non-HCC individuals (**E**); in differentiating HCC patients with an AFP level ≤ 20 ng/mL from LC patients (**F**)

Currently, serum AFP levels, as the only blood-based biomarker for HCC screening, suffers from low accuracy, which severely limits its clinical utility. In biopsy-proven HCC patients, the malignant score demonstrated superior accuracy than AFP (cutoff of 20 ng ml^−1^) in differentiating HCC patients from non-cancerous individuals (AUC 0.957 versus 0.803, Fig. 4A, B). We further compared the performance of these two predictors in 109 early-stage HCC (BCLC stage 0-A) patients. The HCC screening model significantly outperformed than AFP in distinguishing early-stage HCC patients from non-HCC individuals (AUC 0.936 versus 0.764, Fig. 4C). Malignant score also showed a better detection performance than AFP in distinguishing early-stage HCC patients from cirrhotic patients (AUC 0.934 versus 0.719, Fig. 4D).

In addition, we investigated the performance of our model in a set of 61 HCC patients with normal serum AFP (< 20ng ml^−1^), which achieved an AUC of 0.947 (95% CI 0.905–0.963) in differentiating HCC patients with normal AFP from non-HCC individuals. In contrast, AFP only exhibited an AUC of 0.579 (95% CI 0.491–0.667; Fig. 4E) in the same setting. Our model also showed an AUC of 0.930 (95% CI 0.892–0.969) in differentiating HCC patients with normal serum AFP from cirrhotic patients; while AFP had an AUC of 0.617 (95% CI 0.533–0.701; Fig. 4F). Addition of AFP did not statistically improve the overall accuracy of the malignant score (Fig. 4A-F). Collectively, these results revealed that the advantage of malignant score was over AFP in differentiation of the early-stage HCC among the average-risk population and high-risk population.

### Clinical characteristics of false positive and false negative samples

**Fig. 5:**
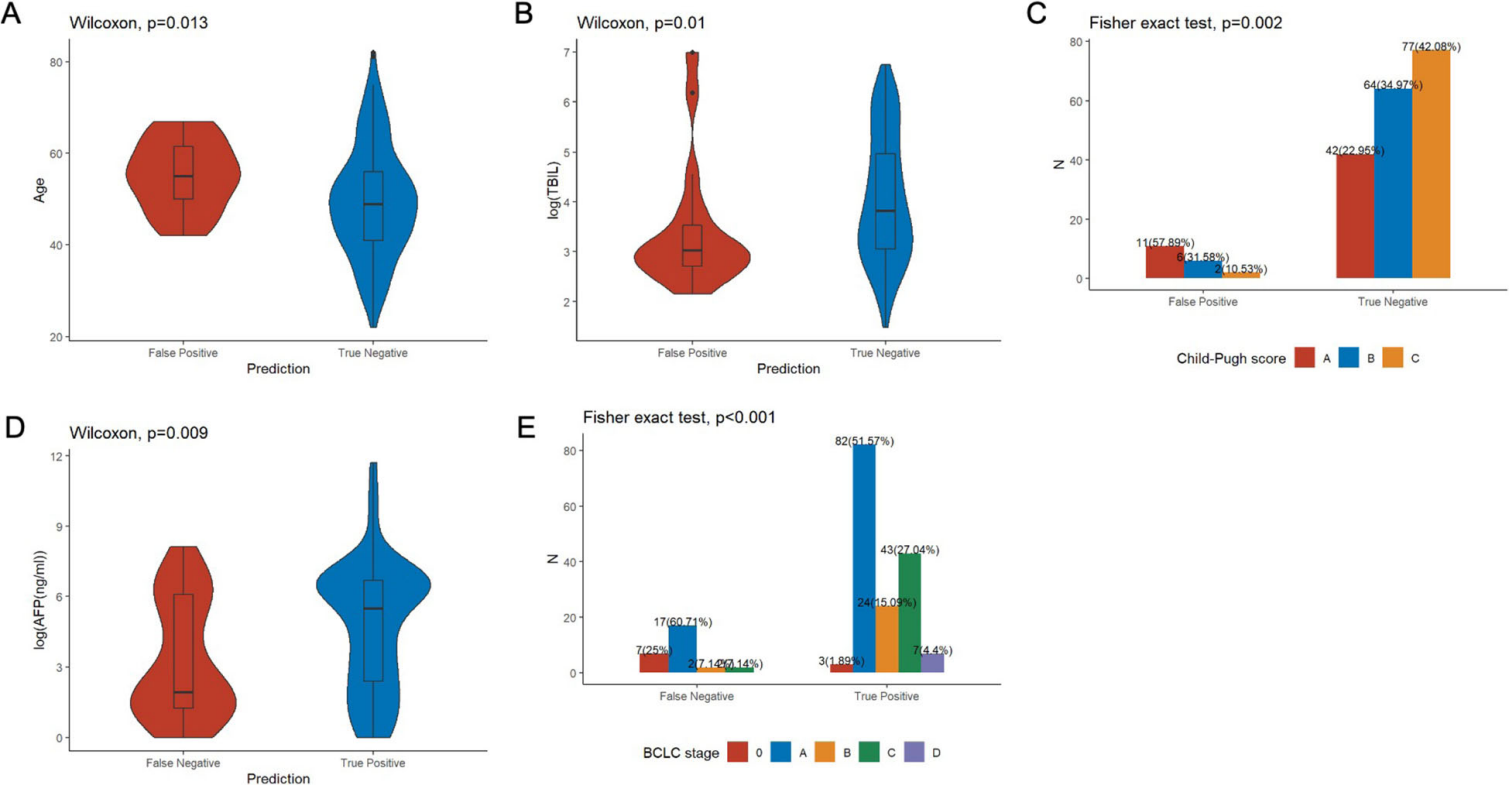
Clinical characteristics of misclassified samples. **A**–**C** Age, bilirubin level, and Child-Pugh score were significantly different between false postives (misclassified LC samples) and true negatives; (**D**, **E)** AFP levels and the distribution of BCLC stage were significantly different between false negatives (misclassified HCC samples) and true postives

To evaluate the clinical characteristics of mis-classified samples, we compared the clinical parameters of 19 false positive cases (cirrhotic samples classified as HCC cases; 6 from training cohort and 13 from validation cohort) and 183 true negative cases (cirrhotic samples classified as non-HCC cases). Our analysis revealed that age, bilirubin level, and Child-Pugh score were significantly different between 19 false positive and 183 true negative samples (Fig. 5A-C). The misclassified patients were older (Wilcoxon, *p*=0.013) with lower bilirubin levels (Wilcoxon, *p*=0.01) than true negatives. The distribution of CP scores in misclassified patients was also significantly lower than true nagetives (Fisher’s exact test, *p*=0.002). The rest investigated clinical parameters were comparable between the two groups, including ALT, AST, and AFP (Additional file 3: Fig. S11A-C). We also compared the clinical parameters between 28 false negative samples (HCC patients classified as non-HCC cases; 17 from training cohort and 11 from validation cohort) and 159 true positive samples (HCC patients classified as HCC cases). False negative cases had a significantly lower AFP (Wilcoxon, *p*=0.09) and earlier BCLC stage than ture positive cases (Fisher’s exact test, *p*<0.001; Fig. 5D, E). The rest clinical parameters were comparable, including age and bilirubin level (Additional file 3: Fig. S11D-E).

## Discussion

Early detection is the most effective way to reduce HCC mortality. In this study, we sought to develop and validate a cfDNA-based multi-layer HCC screening model for the early detection of HCC from patients with liver disease and healthy controls using targeted bisulfite sequencing, a highly sensitive DNA methylation profiling technique based on NGS. A total of 2321 differentially methylated markers were identified by comparing the methylation profiles obtained from HCC, normal, and LC tissue samples. Our model yielded significantly improved performance over serum AFP testing for early-stage HCC versus non-HCC controls, and of the most significant clinical importance, early-stage HCC versus high-risk patients with non-malignant liver disease including LC and HBV infection. Importantly, our model also showed superior performance over AFP by accurately detecting those HCC cases that would have failed to be detected by AFP testing alone. This multi-layer model based on the 2 intermediate outputs, tumor, and benign scores, which primarily reflected the properties of tumors and cirrhosis, achieved differential diagnosis for HCC cases. Taken together, this study suggested that our model had the potentials of becoming an integrated part of HCC surveilance, for early screening of HCC patients from high risk subjects.

Identifying biomarkers for early cancer detection with minimal invasiveness is still an emerging field. Numerous studies have explored the feasibility of ctDNA-based somatic mutation profiles and concluded such technique may not be adequate [23–25]. A tumor cell usually harbors only one copy of mutant DNA. A major challenge associated with utilizing somatic mutation obtained from ctDNA for cancer detection is that an early-stage tumor may not be able release enough copies of the mutant DNA. The TRACERx study revealed that only 13% of stage I lung adenocarcinoma patients had detectable mutations [26]. In a large-scale prospective study, only 27% of early-stage patients were detected by assessing mutations and protein levels in blood testing [27].

Methylation profiling of ctDNA has shown great potential to overcome the limited amount of ctDNA in circulation and the lack of recurrent mutations. DNA methylation alterations occur very early in tumorigenesis, even prior to the emergence of somatic mutation, and also offer an opportunity to identify the early stage cancer before clinical symptoms emerged [16, 28]. Many promising HCC methylation-based screening models have been proposed. Liu et al. reported the study of targeted methylation analysis of circulating cfDNA screening test for over 50 cancer types across stages supported by GRAIL, Inc. [29] 25 patients with stage I–III hepatobiliary cancer were included, demonstrating a sensitivity of 68% at 99% specificity. Kisiel et al. proposed a panel of 6 methylated DNA markers based on differentially expressed genes derived from HCC and control tissues, which achived a sensitivity of 95% and a specificity of 92% when stage I–IV HCC case were detected in high-risk population. Importantly, this panel detected 3/4 stage 0, 39/42 stage A HCC cases [30]. In addition, Xu et al. compared differential DNA methylation profiles of HCC tissues and blood leukocytes to derive 401 candidate markers which were further refined to a panel of 10 markers for the construction of the diagnostic model, yielding a sensitivity of 83.3% and specificity of 90.5% in the validation cohort (stage I–IV HCC cases) [13]. Cai et al. presented a genome-wide 5-hydroxymethylcytosines (5hmC)-based screening model that distinguished early-stage HCC cases (stage 0-A) from the high-risk population, achieving an AUC of 0.884 in the external validation cohort [31]. In addition, DNA methylation alterations could be inflenced by many biological factors, and pre-specified case–control studies may not reflect the full spectrum of the disease owing to selection bias. Carefully well designed studies in the intended use screening population are still required to evaluate the clinical applicability of these studies.

An effective screening assay needs to demonstrate sufficiently high specificity to minimize the risk of overdiagnosis (false positive rate) and to avoid unnecessary anxiety and the follow-up examinations of the non-HCC individuals [32]. Our model have achived high specificities and yeilded 19 false positive samples in both training and validation cohorts. It is possible that these signals were detected from some tumor lesions which were missed by CT-scan screening. These misclassified patients were often senior adults with lower bilirubin levels. We are tracking these false positive individuals to determine whether they have an increased risk of developing HCC.

Despite the significance of our screening model, several limitations might impede the interpretation of our results. Firstly, only cirrhotic patients were included as benign liver disease. Other non-malignant liver diseases would be helpful to improve the model for achieving the most optimal performance. Secondly, the number of enrolled HCC patients in the validation cohort was relatively small, especially the number of the early-stage HCC patients. Additional 0-A stage HCC participants would be helpful to validate the robustness of our model. Thirdly, the LC group in the training cohort has a median age of 47, which is lower than the healthy individuals and HCC patients. An age-matched training set might reduce the potential selection bias. Fourly, given the social economic effectors on the cost of the test at ideal test frequency for intended population as frequent as every 6 months, the current version of the test is not cost-effective enough and might not meet the needs in real-world clinical diagnostics from a social economics perspective. Since methylation-based tests are capable of detecting the cancer and locating the tissue of origin for simultaneously, we anticipate sensitive and cost-effective multi-cancer detection tests will benifit the general public as the technology evovles and more clinical studies have been carried out.

## Conclusions

Collectively, our study provides evidence that cfDNA-based DNA methylation profiling can be used as a non-invasive screening assay for early-stage HCC in clinical settings. We developed a highly sensitive and specific model for HCC early screening, which can accurately distinguish HCC patients from the high risk population with a history of LC or CHB. A multicenter study conducted in the high risk population is needed to further define the efficiency of this model. Based on these results, a prospective Pan-CanceR Early DetectIon ProjeCT (PREDICT study) has been registered in ClinicalTrials.gov with NCT number (NCT04383353) and is ongoing.

## Supplementary Information

**Additional file 1: Table S1**. **Table S1**. 2,321 differentially methylated markers were selected in model construction.

**Additional file 2: Table S2**. **Table S2**. Performance of tissue-derived markers in tissue-samples.

**Additional file 3: Figures S1-S11**. **Fig. S1** The input DNA amount health, benign, and tumor tissue samples in sequencing analysis. **Fig. S2** cfDNA methylation analysis of HCC diagnosis. **Fig. S3** GO and KEGG pathway analysis of benign and cancer signals. **Fig. S4** Unsupervised hierarchical clustering of tissue-derived 2321 methylation markers selected for HCC diagnosis in the tissue samples and plasma samples. **Fig. S5** The specificity and sensitivity of MH model distinguishing tumor from healthy individuals in the training and the validation cohort. **Fig. S6** The specificity and sensitivity of BH model distinguishing tumor from healthy individuals in the training and the validation cohort. **Fig. S7** The comparison of non-tissue tissue-specific markers and tissue-specific markers in BH model contribution. **Fig. S8** The specificity and sensitivity of MB model distinguishing tumor from healthy individuals in the training and the validation cohort. **Fig. S9** The ROC of the HCC screening model (malignant score) for HCC diagnosis in the training and the validation cohort. **Fig. S10** The correlation of malignant score and benign score with cause of cirrhosis (HCV or non-HCV). **Fig. S11** Clinical characteristics of misclassified cirrhotic samples.

## Data Availability

The datasets used and/or analyzed during the current study are available from the corresponding author on reasonable request.

## Abbreviations

cfDNA: Cell-free DNA
ctDNA: Circulating tumor DNA
HCC: Hepatocellular carcinoma
ROC: Receiver operating characteristic
AUC: Area under the curve
CI: Confidence interval
HR: Hazard ratio
AFP: Alpha-fetoprotein
HBV: Hepatitis B virus
HCV: Hepatitis C virus
CHB: Chronic hepatitis B
LC: Liver cirrhosis
HBsAg: Hepatitis B surface antigen

## References

[1] Torre LA, Bray F, Siegel RL, Ferlay J, Lortet-Tieulent J, Jemal A (2015). Global cancer statistics, 2012. CA: Cancer J Clin.

[2] Fuchs BC, Hoshida Y, Fujii T, Wei L, Yamada S, Lauwers GY (2014). Epidermal growth factor receptor inhibition attenuates liver fibrosis and development of hepatocellular carcinoma. Hepatology.

[3] Zhang DY, Friedman SL (2012). Fibrosis-dependent mechanisms of hepatocarcinogenesis. Hepatology.

[4] Bruix J, Sherman M (2011). AASLD practice guideline: management of hepatocellular carcinoma: an update. Hepatol (Baltimore, Md).

[5] Marrero J, Feng Z, Wang Y, Nguyen M, Befeler A, Roberts L (2009). Alpha-fetoprotein, des-gamma carboxyprothrombin, and lectin-bound alpha-fetoprotein in early hepatocellular carcinoma. Gastroenterology.

[6] The Origin and Mechanism of Circulating DNA. Annals of the New York Academy of Sciences. 2000;906(CIRCULATING NUCLEIC ACIDS IN PLASMA OR SERUM):161-8. 10.1111/j.1749-6632.2000.tb06608.x 10818614

[7] Bruix J, Sherman M (2011). Management of hepatocellular carcinoma: an update. Hepatology.

[8] Yu NC, Chaudhari V, Raman SS, Lassman C, Tong MJ, Busuttil RW (2011). CT and MRI improve detection of hepatocellular carcinoma, compared with ultrasound alone, in patients with cirrhosis. Clin Gastroenterol Hepatol.

[9] Luo P, Wu S, Yu Y, Ming X, Li S, Zuo X, et al. Current status and perspective biomarkers in AFP negative HCC: towards screening for and diagnosing hepatocellular carcinoma at an earlier stage. Pathol Oncol Res. 2019. 10.1007/s12253-019-00585-5 30661224

[10] Tsuchiya N, Sawada Y, Endo I, Saito K, Uemura Y, Nakatsura T (2015). Biomarkers for the early diagnosis of hepatocellular carcinoma. World J Gastroenterol.

[11] Kladney RD, Cui X, Bulla GA, Brunt EM, Fimmel CJ (2002). Expression of GP73, a resident Golgi membrane protein, in viral and nonviral liver disease. Hepatology.

[12] Feng J, Zhu R, Chang C, Yu L, Sun L (2016). CK19 and glypican 3 expression profiling in the prognostic indication for patients with HCC after surgical resection. Plos ONE.

[13] Bettegowda C, Sausen M, Leary RJ, Kinde I, Wang Y, Agrawal N (2014). Detection of circulating tumor DNA in early- and late-stage human malignancies. Sci Transl Med.

[14] Chaudhuri AA, Chabon JJ, Lovejoy AF, Newman AM, Stehr H, Azad TD (2017). Early detection of molecular residual disease in localized lung cancer by circulating tumor DNA profiling. Cancer Disc.

[15] Cohen J, Li L, Wang Y, Thoburn C, Afsari B, Danilova L (2018). Detection and localization of surgically resectable cancers with a multi-analyte blood test. Sci(New York, NY).

[16] Baylin SB, Jones PA (2011). A decade of exploring the cancer epigenome—biological and translational implications. Nat Rev Cancer.

[17] Irizarry RA, Ladd-Acosta C, Wen B, Wu Z, Montano C, Onyango P (2009). The human colon cancer methylome shows similar hypo- and hypermethylation at conserved tissue-specific CpG island shores. Nat Genet.

[18] Toyota M, Ahuja N, Ohe-Toyota M, Herman JG, Baylin SB, Issa JP (1999). CpG island methylator phenotype in colorectal cancer. Proc Natl Acad Sci U S A.

[19] Chan KC, Jiang P, Chan CW, Sun K, Wong J, Hui EP (2013). Noninvasive detection of cancer-associated genome-wide hypomethylation and copy number aberrations by plasma DNA bisulfite sequencing. Proc Natl Acad Sci U S A.

[20] Wen L, Li J, Guo H, Liu X, Zheng S, Zhang D (2015). Genome-scale detection of hypermethylated CpG islands in circulating cell-free DNA of hepatocellular carcinoma patients. Cell Res.

[21] Liang N, Li B, Jia Z, Wang C, Wu P, Zheng T (2021). Ultrasensitive detection of circulating tumour DNA via deep methylation sequencing aided by machine learning. Nat Biomed Eng.

[22] Pedersen BS, Eyring K, De S, Yang IV, Schwartz DA (2014). Fast Accurate Alignment Long Bisulfite-seq Reads.

[23] Gao J, Wang H, Zang W, Li B, Rao G, Li L (2017). Circulating tumor DNA functions as an alternative for tissue to overcome tumor heterogeneity in advanced gastric cancer. Cancer Sci.

[24] Mack P, Banks K, Espenschied C, Burich R, Zill O, Lee C (2020). Spectrum of driver mutations and clinical impact of circulating tumor DNA analysis in non-small cell lung cancer: Analysis of over 8000 cases. Cancer.

[25] Noguchi T, Iwahashi N, Sakai K, Matsuda K, Matsukawa H, Toujima S, et al. Comprehensive gene mutation profiling of circulating tumor DNA in ovarian cancer: its pathological and prognostic impact. Cancers. 2020;12(11). 10.3390/cancers12113382 PMC7697720 33207545

[26] Abbosh C, Birkbak NJ, Wilson GA, Jamal-Hanjani M, Constantin T, Salari R (2017). Phylogenetic ctDNA analysis depicts early-stage lung cancer evolution. Nature.

[27] Lennon AM, Buchanan AH, Kinde I, Warren A, Honushefsky A, Cohain AT, et al. Feasibility of blood testing combined with PET-CT to screen for cancer and guide intervention. Science. 2020;369(6499). 10.1126/science.abb9601 PMC7509949 32345712

[28] Baylin SB, Jones PA. Epigenetic determinants of cancer. Cold Spring Harb Perspect Biol. 2016;8(9). 10.1101/cshperspect.a019505 PMC5008069 27194046

[29] Liu M, Oxnard G, Klein E, Swanton C, Seiden M (2020). Sensitive and specific multi-cancer detection and localization using methylation signatures in cell-free DNA. Ann Oncol Off J Eur Soc Med Onco.

[30] Kisiel JB, Dukek BA, V.S.R. Kanipakam R, Ghoz HM, Yab TC, Berger CK (2019). Hepatocellular carcinoma detection by plasma methylated DNA: Discovery, Phase I Pilot, and Phase II Clinical Validation. Hepatology.

[31] Cai J, Chen L, Zhang Z, Zhang X, Lu X, Liu W (2019). Genome-wide mapping of 5-hydroxymethylcytosines in circulating cell-free DNA as a non-invasive approach for early detection of hepatocellular carcinoma. Gut.

[32] Siegel RL, Miller KD, Jemal A (2019). Cancer statistics, 2019. CA Cancer J Clin.

