# Supplementary material for: Cell-free DNA methylation markers for differential diagnosis of hepatocellular carcinoma
Source: BMC Med. 2022 Jan 14;20:8. doi: 10.1186/s12916-021-02201-3 (PMC8759185; doi:10.1186/s12916-021-02201-3)
Supplement: Supplementary file 1 — Additional file 1: Table S1. Table S1. 2,321 differentially methylated markers were selected in model construction. [file 12916_2021_2201_MOESM1_ESM.docx]

Supplementary Table 1: 2,321 differentially methylated markers were selected in model construction. Differentially methylated markers were grouped in cancer-specific, tissue-specific or both, and hypermethylation or hypomethylation.

| **Chr** | **Start** | **End** | **Group** | **Methylation Type** |
| --- | --- | --- | --- | --- |
| 10 | 101280170 | 101280280 | Cancer-specific | hypermethylation |
| 10 | 101280295 | 101280382 | Cancer-specific | hypermethylation |
| 10 | 101283432 | 101283493 | Cancer-specific | hypermethylation |
| 10 | 101287803 | 101287926 | Cancer-specific | hypermethylation |
| 10 | 101292470 | 101292574 | Cancer-specific | hypermethylation |
| 10 | 101293826 | 101293899 | Cancer-specific | hypermethylation |
| 10 | 101294524 | 101294602 | Cancer-specific | hypermethylation |
| 10 | 101294604 | 101294744 | Cancer-specific | hypermethylation |
| 10 | 101295738 | 101295848 | Cancer-specific | hypermethylation |
| 10 | 102419086 | 102419230 | Cancer-specific | hypermethylation |
| 10 | 102419238 | 102419320 | Cancer-specific | hypermethylation |
| 10 | 102473600 | 102473724 | Cancer-specific | hypermethylation |
| 10 | 102484106 | 102484239 | Cancer-specific | hypermethylation |
| 10 | 102484230 | 102484357 | Cancer-specific | hypermethylation |
| 10 | 102495234 | 102495341 | Cancer-specific | hypermethylation |
| 10 | 102495347 | 102495460 | Cancer-specific | hypermethylation |
| 10 | 102497563 | 102497642 | Cancer-specific | hypermethylation |
| 10 | 102893866 | 102894098 | Cancer-specific | hypermethylation |
| 10 | 102894110 | 102894283 | Cancer-specific | hypermethylation |
| 10 | 102894540 | 102894646 | Cancer-specific | hypermethylation |
| 10 | 102894641 | 102894716 | Cancer-specific | hypermethylation |
| 10 | 102894923 | 102895027 | Cancer-specific | hypermethylation |
| 10 | 102895027 | 102895146 | Cancer-specific | hypermethylation |
| 10 | 102900004 | 102900148 | Cancer-specific | hypermethylation |
| 10 | 102900142 | 102900255 | Cancer-specific | hypermethylation |
| 10 | 102986856 | 102986973 | Cancer-specific | hypermethylation |
| 10 | 102986966 | 102987094 | Cancer-specific | hypermethylation |
| 10 | 102988140 | 102988248 | Cancer-specific | hypermethylation |
| 10 | 103043884 | 103044040 | Cancer-specific | hypermethylation |
| 10 | 103044071 | 103044133 | Cancer-specific | hypermethylation |
| 10 | 105036584 | 105036784 | Cancer-specific | hypermethylation |
| 10 | 105239078 | 105239434 | Both | hypomethylation |
| 10 | 106399561 | 106399629 | Cancer-specific | hypermethylation |
| 10 | 115514694 | 115514981 | Both | hypomethylation |
| 10 | 118892463 | 118892642 | Cancer-specific | hypermethylation |
| 10 | 118893040 | 118893166 | Cancer-specific | hypermethylation |
| 10 | 118893167 | 118893259 | Cancer-specific | hypermethylation |
| 10 | 118897726 | 118897807 | Cancer-specific | hypermethylation |
| 10 | 119297347 | 119297544 | Cancer-specific | hypermethylation |
| 10 | 119297539 | 119297668 | Cancer-specific | hypermethylation |
| 10 | 119301635 | 119301683 | Cancer-specific | hypermethylation |
| 10 | 119301716 | 119301835 | Cancer-specific | hypermethylation |
| 10 | 119301926 | 119302026 | Cancer-specific | hypermethylation |
| 10 | 119303098 | 119303232 | Cancer-specific | hypermethylation |
| 10 | 124514653 | 124514822 | Both | hypomethylation |
| 10 | 124910909 | 124910996 | Cancer-specific | hypermethylation |
| 10 | 126765263 | 126765630 | Both | hypomethylation |
| 10 | 128076471 | 128076664 | Cancer-specific | hypermethylation |
| 10 | 128076787 | 128076874 | Cancer-specific | hypermethylation |
| 10 | 128076903 | 128077024 | Cancer-specific | hypermethylation |
| 10 | 130085081 | 130085206 | Cancer-specific | hypermethylation |
| 10 | 133208827 | 133209007 | Both | hypomethylation |
| 10 | 133209020 | 133209131 | Both | hypomethylation |
| 10 | 133318076 | 133318215 | Cancer-specific | hypomethylation |
| 10 | 133318222 | 133318414 | Both | hypomethylation |
| 10 | 133850648 | 133850733 | Cancer-specific | hypermethylation |
| 10 | 133850753 | 133850832 | Cancer-specific | hypermethylation |
| 10 | 134000002 | 134000131 | Cancer-specific | hypermethylation |
| 10 | 134598804 | 134598902 | Cancer-specific | hypermethylation |
| 10 | 134598897 | 134599042 | Cancer-specific | hypermethylation |
| 10 | 134599714 | 134600215 | Cancer-specific | hypermethylation |
| 10 | 134828016 | 134828372 | Cancer-specific | hypomethylation |
| 10 | 135043988 | 135044101 | Cancer-specific | hypermethylation |
| 10 | 135044107 | 135044220 | Cancer-specific | hypermethylation |
| 10 | 135092053 | 135092245 | Tissue-specific | hypomethylation |
| 10 | 135092257 | 135092339 | Tissue-specific | hypomethylation |
| 10 | 15761862 | 15761959 | Cancer-specific | hypermethylation |
| 10 | 16562508 | 16562651 | Cancer-specific | hypermethylation |
| 10 | 16562654 | 16562751 | Cancer-specific | hypermethylation |
| 10 | 1714259 | 1714417 | Both | hypomethylation |
| 10 | 1714445 | 1714635 | Cancer-specific | hypomethylation |
| 10 | 17269595 | 17269735 | Tissue-specific | hypomethylation |
| 10 | 17270250 | 17270451 | Cancer-specific | hypermethylation |
| 10 | 17270465 | 17270608 | Cancer-specific | hypermethylation |
| 10 | 17270597 | 17270709 | Cancer-specific | hypermethylation |
| 10 | 17271108 | 17271301 | Cancer-specific | hypermethylation |
| 10 | 17271296 | 17271462 | Cancer-specific | hypermethylation |
| 10 | 17271459 | 17271639 | Cancer-specific | hypermethylation |
| 10 | 22623970 | 22624117 | Cancer-specific | hypermethylation |
| 10 | 22624555 | 22624763 | Cancer-specific | hypermethylation |
| 10 | 22634308 | 22634423 | Cancer-specific | hypermethylation |
| 10 | 22634425 | 22634544 | Cancer-specific | hypermethylation |
| 10 | 22765574 | 22765828 | Cancer-specific | hypermethylation |
| 10 | 22765833 | 22766088 | Cancer-specific | hypermethylation |
| 10 | 23216720 | 23216903 | Cancer-specific | hypermethylation |
| 10 | 23216903 | 23216966 | Cancer-specific | hypermethylation |
| 10 | 23463008 | 23463213 | Cancer-specific | hypermethylation |
| 10 | 23463250 | 23463384 | Cancer-specific | hypermethylation |
| 10 | 23463375 | 23463500 | Cancer-specific | hypermethylation |
| 10 | 23484433 | 23484504 | Cancer-specific | hypermethylation |
| 10 | 23484549 | 23484611 | Cancer-specific | hypermethylation |
| 10 | 23984816 | 23984894 | Cancer-specific | hypermethylation |
| 10 | 25464606 | 25464730 | Cancer-specific | hypermethylation |
| 10 | 25464735 | 25464832 | Cancer-specific | hypermethylation |
| 10 | 25465423 | 25465548 | Cancer-specific | hypermethylation |
| 10 | 28034037 | 28034115 | Cancer-specific | hypermethylation |
| 10 | 29811415 | 29811633 | Tissue-specific | hypermethylation |
| 10 | 29811652 | 29811779 | Cancer-specific | hypermethylation |
| 10 | 31074024 | 31074084 | Cancer-specific | hypermethylation |
| 10 | 3110948 | 3111064 | Cancer-specific | hypermethylation |
| 10 | 43249953 | 43250094 | Cancer-specific | hypermethylation |
| 10 | 43428344 | 43428392 | Cancer-specific | hypermethylation |
| 10 | 43428409 | 43428506 | Cancer-specific | hypermethylation |
| 10 | 43818316 | 43818443 | Cancer-specific | hypermethylation |
| 10 | 43818452 | 43818544 | Cancer-specific | hypermethylation |
| 10 | 45914721 | 45914806 | Cancer-specific | hypermethylation |
| 10 | 45914822 | 45914956 | Cancer-specific | hypermethylation |
| 10 | 54068565 | 54068751 | Cancer-specific | hypermethylation |
| 10 | 57389848 | 57389894 | Cancer-specific | hypermethylation |
| 10 | 57390184 | 57390459 | Cancer-specific | hypermethylation |
| 10 | 63422504 | 63422594 | Tissue-specific | hypermethylation |
| 10 | 63422599 | 63422751 | Cancer-specific | hypermethylation |
| 10 | 63422752 | 63422967 | Cancer-specific | hypermethylation |
| 10 | 72200499 | 72200614 | Cancer-specific | hypermethylation |
| 10 | 72200605 | 72200718 | Cancer-specific | hypermethylation |
| 10 | 72200915 | 72201020 | Cancer-specific | hypermethylation |
| 10 | 72201010 | 72201133 | Cancer-specific | hypermethylation |
| 10 | 7453208 | 7453360 | Cancer-specific | hypermethylation |
| 10 | 83455561 | 83455603 | Both | hypomethylation |
| 10 | 85958393 | 85958458 | Cancer-specific | hypomethylation |
| 10 | 91351220 | 91351418 | Both | hypomethylation |
| 10 | 93647465 | 93647598 | Cancer-specific | hypermethylation |
| 10 | 93647596 | 93647726 | Cancer-specific | hypermethylation |
| 10 | 94180257 | 94180383 | Cancer-specific | hypermethylation |
| 10 | 94180376 | 94180508 | Cancer-specific | hypermethylation |
| 10 | 94828022 | 94828142 | Cancer-specific | hypermethylation |
| 10 | 94828134 | 94828228 | Cancer-specific | hypermethylation |
| 11 | 101454553 | 101454655 | Cancer-specific | hypermethylation |
| 11 | 101454669 | 101454772 | Cancer-specific | hypermethylation |
| 11 | 104034111 | 104034234 | Cancer-specific | hypermethylation |
| 11 | 104034233 | 104034339 | Both | hypermethylation |
| 11 | 105481175 | 105481290 | Cancer-specific | hypermethylation |
| 11 | 105481285 | 105481523 | Cancer-specific | hypermethylation |
| 11 | 106888767 | 106888844 | Cancer-specific | hypermethylation |
| 11 | 107799541 | 107799618 | Cancer-specific | hypermethylation |
| 11 | 111411091 | 111411319 | Cancer-specific | hypermethylation |
| 11 | 111411316 | 111411430 | Cancer-specific | hypermethylation |
| 11 | 111411418 | 111411507 | Cancer-specific | hypermethylation |
| 11 | 111411629 | 111411745 | Cancer-specific | hypermethylation |
| 11 | 111411749 | 111411874 | Cancer-specific | hypermethylation |
| 11 | 113346272 | 113346395 | Cancer-specific | hypermethylation |
| 11 | 116451115 | 116451247 | Cancer-specific | hypermethylation |
| 11 | 116451242 | 116451315 | Cancer-specific | hypermethylation |
| 11 | 116700429 | 116700563 | Both | hypomethylation |
| 11 | 12030066 | 12030194 | Cancer-specific | hypermethylation |
| 11 | 12030194 | 12030336 | Cancer-specific | hypermethylation |
| 11 | 12030324 | 12030554 | Cancer-specific | hypermethylation |
| 11 | 12030925 | 12031155 | Cancer-specific | hypermethylation |
| 11 | 120434948 | 120435076 | Both | hypermethylation |
| 11 | 120435070 | 120435173 | Both | hypermethylation |
| 11 | 121800425 | 121800504 | Cancer-specific | hypermethylation |
| 11 | 124056237 | 124056374 | Both | hypomethylation |
| 11 | 124735126 | 124735231 | Cancer-specific | hypermethylation |
| 11 | 12621073 | 12621087 | Both | hypomethylation |
| 11 | 13030796 | 13030910 | Cancer-specific | hypermethylation |
| 11 | 13030902 | 13030994 | Cancer-specific | hypermethylation |
| 11 | 131780269 | 131780388 | Cancer-specific | hypermethylation |
| 11 | 131780377 | 131780459 | Cancer-specific | hypermethylation |
| 11 | 131781137 | 131781278 | Cancer-specific | hypermethylation |
| 11 | 132643314 | 132643422 | Cancer-specific | hypomethylation |
| 11 | 132917412 | 132917490 | Both | hypomethylation |
| 11 | 132917500 | 132917576 | Cancer-specific | hypomethylation |
| 11 | 134213665 | 134214015 | Both | hypomethylation |
| 11 | 14995194 | 14995240 | Cancer-specific | hypermethylation |
| 11 | 16023697 | 16023723 | Both | hypomethylation |
| 11 | 16023841 | 16023855 | Tissue-specific | hypomethylation |
| 11 | 16632223 | 16632461 | Both | hypermethylation |
| 11 | 16632502 | 16632731 | Both | hypermethylation |
| 11 | 17565760 | 17565885 | Cancer-specific | hypermethylation |
| 11 | 17565878 | 17565964 | Cancer-specific | hypermethylation |
| 11 | 1769949 | 1770110 | Cancer-specific | hypermethylation |
| 11 | 1770109 | 1770188 | Cancer-specific | hypermethylation |
| 11 | 18258048 | 18258379 | Both | hypomethylation |
| 11 | 19263801 | 19263918 | Cancer-specific | hypermethylation |
| 11 | 19735718 | 19735814 | Cancer-specific | hypermethylation |
| 11 | 20182200 | 20182266 | Cancer-specific | hypermethylation |
| 11 | 20618817 | 20618969 | Cancer-specific | hypermethylation |
| 11 | 2223080 | 2223094 | Cancer-specific | hypomethylation |
| 11 | 2291526 | 2291687 | Cancer-specific | hypermethylation |
| 11 | 2291695 | 2291807 | Cancer-specific | hypermethylation |
| 11 | 2291873 | 2291954 | Cancer-specific | hypermethylation |
| 11 | 2291965 | 2292113 | Cancer-specific | hypermethylation |
| 11 | 2292320 | 2292435 | Cancer-specific | hypermethylation |
| 11 | 2292430 | 2292565 | Cancer-specific | hypermethylation |
| 11 | 30605928 | 30606116 | Cancer-specific | hypermethylation |
| 11 | 31819373 | 31819430 | Cancer-specific | hypermethylation |
| 11 | 31819437 | 31819505 | Cancer-specific | hypermethylation |
| 11 | 31821264 | 31821362 | Cancer-specific | hypermethylation |
| 11 | 31821361 | 31821504 | Cancer-specific | hypermethylation |
| 11 | 31822208 | 31822372 | Cancer-specific | hypermethylation |
| 11 | 31822372 | 31822562 | Cancer-specific | hypermethylation |
| 11 | 31825962 | 31826088 | Cancer-specific | hypermethylation |
| 11 | 31826298 | 31826498 | Cancer-specific | hypermethylation |
| 11 | 31826551 | 31826700 | Cancer-specific | hypermethylation |
| 11 | 31827750 | 31827856 | Cancer-specific | hypermethylation |
| 11 | 31838650 | 31838799 | Cancer-specific | hypermethylation |
| 11 | 31839201 | 31839470 | Cancer-specific | hypermethylation |
| 11 | 31839514 | 31839643 | Cancer-specific | hypermethylation |
| 11 | 31840573 | 31840722 | Cancer-specific | hypermethylation |
| 11 | 31840723 | 31840821 | Cancer-specific | hypermethylation |
| 11 | 31841420 | 31841543 | Cancer-specific | hypermethylation |
| 11 | 31841558 | 31841649 | Cancer-specific | hypermethylation |
| 11 | 31848481 | 31848621 | Cancer-specific | hypermethylation |
| 11 | 31848634 | 31848754 | Cancer-specific | hypermethylation |
| 11 | 32454868 | 32454994 | Cancer-specific | hypermethylation |
| 11 | 32455007 | 32455088 | Cancer-specific | hypermethylation |
| 11 | 43602720 | 43602833 | Cancer-specific | hypermethylation |
| 11 | 43602838 | 43603004 | Cancer-specific | hypermethylation |
| 11 | 44325697 | 44325827 | Cancer-specific | hypermethylation |
| 11 | 44325816 | 44325939 | Cancer-specific | hypermethylation |
| 11 | 44326284 | 44326427 | Cancer-specific | hypermethylation |
| 11 | 44326417 | 44326523 | Cancer-specific | hypermethylation |
| 11 | 45377235 | 45377337 | Cancer-specific | hypermethylation |
| 11 | 57364932 | 57365058 | Both | hypomethylation |
| 11 | 57405669 | 57405717 | Both | hypermethylation |
| 11 | 57405764 | 57405897 | Cancer-specific | hypermethylation |
| 11 | 58672761 | 58672886 | Cancer-specific | hypermethylation |
| 11 | 58672878 | 58673007 | Cancer-specific | hypermethylation |
| 11 | 58673329 | 58673484 | Cancer-specific | hypermethylation |
| 11 | 58673520 | 58673560 | Cancer-specific | hypermethylation |
| 11 | 60282391 | 60282623 | Both | hypomethylation |
| 11 | 60718746 | 60718889 | Cancer-specific | hypermethylation |
| 11 | 60718878 | 60718991 | Cancer-specific | hypermethylation |
| 11 | 62476434 | 62476476 | Cancer-specific | hypermethylation |
| 11 | 62476519 | 62476569 | Cancer-specific | hypermethylation |
| 11 | 62476945 | 62477189 | Cancer-specific | hypermethylation |
| 11 | 62477220 | 62477380 | Cancer-specific | hypermethylation |
| 11 | 637035 | 637180 | Cancer-specific | hypermethylation |
| 11 | 637168 | 637284 | Cancer-specific | hypermethylation |
| 11 | 64136122 | 64136220 | Tissue-specific | hypermethylation |
| 11 | 64480558 | 64480700 | Cancer-specific | hypermethylation |
| 11 | 64480690 | 64480807 | Cancer-specific | hypermethylation |
| 11 | 64512963 | 64513143 | Cancer-specific | hypermethylation |
| 11 | 64513149 | 64513275 | Cancer-specific | hypermethylation |
| 11 | 65601075 | 65601135 | Cancer-specific | hypermethylation |
| 11 | 66081358 | 66081449 | Tissue-specific | hypermethylation |
| 11 | 66326640 | 66326887 | Cancer-specific | hypermethylation |
| 11 | 69634390 | 69634498 | Cancer-specific | hypermethylation |
| 11 | 71955479 | 71955584 | Cancer-specific | hypermethylation |
| 11 | 7273354 | 7273554 | Cancer-specific | hypermethylation |
| 11 | 74178148 | 74178275 | Cancer-specific | hypermethylation |
| 11 | 74178272 | 74178385 | Cancer-specific | hypermethylation |
| 11 | 75379484 | 75379661 | Cancer-specific | hypermethylation |
| 11 | 75379651 | 75379764 | Cancer-specific | hypermethylation |
| 11 | 75379755 | 75379888 | Cancer-specific | hypermethylation |
| 11 | 75379911 | 75379987 | Cancer-specific | hypermethylation |
| 11 | 8102352 | 8102431 | Cancer-specific | hypermethylation |
| 11 | 8102422 | 8102528 | Cancer-specific | hypermethylation |
| 11 | 8190539 | 8190629 | Cancer-specific | hypermethylation |
| 11 | 8190619 | 8190733 | Cancer-specific | hypermethylation |
| 11 | 8290282 | 8290328 | Cancer-specific | hypermethylation |
| 11 | 85437536 | 85437841 | Cancer-specific | hypomethylation |
| 11 | 8615552 | 8615733 | Cancer-specific | hypermethylation |
| 11 | 8615730 | 8615952 | Cancer-specific | hypermethylation |
| 11 | 92703141 | 92703224 | Cancer-specific | hypermethylation |
| 11 | 92703290 | 92703440 | Cancer-specific | hypermethylation |
| 11 | 94134639 | 94134701 | Cancer-specific | hypermethylation |
| 12 | 103352333 | 103352475 | Cancer-specific | hypermethylation |
| 12 | 103352496 | 103352575 | Cancer-specific | hypermethylation |
| 12 | 104697119 | 104697241 | Cancer-specific | hypermethylation |
| 12 | 104697305 | 104697638 | Cancer-specific | hypermethylation |
| 12 | 104852226 | 104852374 | Cancer-specific | hypermethylation |
| 12 | 104852367 | 104852555 | Cancer-specific | hypermethylation |
| 12 | 106974500 | 106974563 | Cancer-specific | hypermethylation |
| 12 | 108297619 | 108297682 | Cancer-specific | hypermethylation |
| 12 | 113228538 | 113228552 | Cancer-specific | hypomethylation |
| 12 | 113901083 | 113901331 | Cancer-specific | hypermethylation |
| 12 | 113901410 | 113901507 | Cancer-specific | hypermethylation |
| 12 | 115135852 | 115136010 | Cancer-specific | hypermethylation |
| 12 | 115136168 | 115136294 | Cancer-specific | hypermethylation |
| 12 | 115136291 | 115136356 | Cancer-specific | hypermethylation |
| 12 | 122667555 | 122667679 | Cancer-specific | hypermethylation |
| 12 | 122667672 | 122667743 | Cancer-specific | hypermethylation |
| 12 | 125533921 | 125534029 | Cancer-specific | hypermethylation |
| 12 | 125534036 | 125534166 | Cancer-specific | hypermethylation |
| 12 | 128751913 | 128752025 | Cancer-specific | hypermethylation |
| 12 | 128752033 | 128752154 | Cancer-specific | hypermethylation |
| 12 | 130388896 | 130388969 | Cancer-specific | hypermethylation |
| 12 | 130527074 | 130527123 | Cancer-specific | hypermethylation |
| 12 | 130823619 | 130823747 | Cancer-specific | hypermethylation |
| 12 | 130823740 | 130823863 | Cancer-specific | hypermethylation |
| 12 | 131246537 | 131246595 | Tissue-specific | hypomethylation |
| 12 | 132102061 | 132102454 | Both | hypomethylation |
| 12 | 133195101 | 133195203 | Cancer-specific | hypermethylation |
| 12 | 133481407 | 133481527 | Cancer-specific | hypermethylation |
| 12 | 133481521 | 133481596 | Cancer-specific | hypermethylation |
| 12 | 133484914 | 133485054 | Cancer-specific | hypermethylation |
| 12 | 133485058 | 133485343 | Cancer-specific | hypermethylation |
| 12 | 133485332 | 133485588 | Cancer-specific | hypermethylation |
| 12 | 133485584 | 133485823 | Cancer-specific | hypermethylation |
| 12 | 14849173 | 14849512 | Both | hypomethylation |
| 12 | 15475584 | 15475666 | Cancer-specific | hypermethylation |
| 12 | 16759775 | 16759800 | Both | hypermethylation |
| 12 | 21810364 | 21810532 | Cancer-specific | hypermethylation |
| 12 | 21810534 | 21810609 | Cancer-specific | hypermethylation |
| 12 | 286662 | 286761 | Cancer-specific | hypomethylation |
| 12 | 291703 | 291838 | Both | hypermethylation |
| 12 | 33592157 | 33592266 | Cancer-specific | hypermethylation |
| 12 | 33592263 | 33592401 | Cancer-specific | hypermethylation |
| 12 | 33592529 | 33592649 | Cancer-specific | hypermethylation |
| 12 | 33592652 | 33592751 | Cancer-specific | hypermethylation |
| 12 | 42326346 | 42326427 | Cancer-specific | hypermethylation |
| 12 | 42326700 | 42326769 | Cancer-specific | hypermethylation |
| 12 | 4381592 | 4381865 | Cancer-specific | hypermethylation |
| 12 | 4383692 | 4383865 | Cancer-specific | hypermethylation |
| 12 | 43944761 | 43944930 | Cancer-specific | hypermethylation |
| 12 | 43944928 | 43945051 | Cancer-specific | hypermethylation |
| 12 | 45269020 | 45269105 | Cancer-specific | hypermethylation |
| 12 | 48397441 | 48397597 | Cancer-specific | hypermethylation |
| 12 | 48397612 | 48397691 | Cancer-specific | hypermethylation |
| 12 | 48578096 | 48578199 | Cancer-specific | hypermethylation |
| 12 | 4919049 | 4919154 | Cancer-specific | hypermethylation |
| 12 | 49297811 | 49297928 | Cancer-specific | hypermethylation |
| 12 | 49297950 | 49298092 | Cancer-specific | hypermethylation |
| 12 | 5019132 | 5019249 | Cancer-specific | hypermethylation |
| 12 | 5019489 | 5019605 | Cancer-specific | hypermethylation |
| 12 | 52214765 | 52214906 | Cancer-specific | hypermethylation |
| 12 | 52214909 | 52215010 | Cancer-specific | hypermethylation |
| 12 | 54321654 | 54321778 | Cancer-specific | hypermethylation |
| 12 | 54611321 | 54611481 | Cancer-specific | hypermethylation |
| 12 | 54611522 | 54611713 | Cancer-specific | hypermethylation |
| 12 | 58025892 | 58026051 | Cancer-specific | hypermethylation |
| 12 | 62584949 | 62585082 | Cancer-specific | hypermethylation |
| 12 | 64784127 | 64784373 | Cancer-specific | hypermethylation |
| 12 | 64784374 | 64784599 | Cancer-specific | hypermethylation |
| 12 | 66122782 | 66123047 | Cancer-specific | hypermethylation |
| 12 | 66216901 | 66216995 | Cancer-specific | hypermethylation |
| 12 | 85305620 | 85305806 | Cancer-specific | hypermethylation |
| 12 | 85306358 | 85306601 | Cancer-specific | hypermethylation |
| 12 | 85306689 | 85306900 | Cancer-specific | hypermethylation |
| 12 | 85673193 | 85673250 | Cancer-specific | hypermethylation |
| 12 | 85673263 | 85673360 | Cancer-specific | hypermethylation |
| 12 | 95941862 | 95942008 | Cancer-specific | hypermethylation |
| 12 | 95942002 | 95942107 | Cancer-specific | hypermethylation |
| 12 | 95942634 | 95942985 | Cancer-specific | hypermethylation |
| 12 | 99139460 | 99139685 | Cancer-specific | hypermethylation |
| 12 | 99139679 | 99139833 | Cancer-specific | hypermethylation |
| 12 | 99289311 | 99289405 | Cancer-specific | hypermethylation |
| 13 | 100624143 | 100624241 | Cancer-specific | hypermethylation |
| 13 | 100627232 | 100627365 | Cancer-specific | hypermethylation |
| 13 | 100627359 | 100627467 | Cancer-specific | hypermethylation |
| 13 | 100641522 | 100641612 | Cancer-specific | hypermethylation |
| 13 | 100641632 | 100641765 | Cancer-specific | hypermethylation |
| 13 | 100642005 | 100642115 | Cancer-specific | hypermethylation |
| 13 | 100642112 | 100642194 | Cancer-specific | hypermethylation |
| 13 | 100642662 | 100642806 | Cancer-specific | hypermethylation |
| 13 | 100642815 | 100642892 | Cancer-specific | hypermethylation |
| 13 | 100643197 | 100643319 | Cancer-specific | hypermethylation |
| 13 | 100643322 | 100643428 | Cancer-specific | hypermethylation |
| 13 | 103054940 | 103054994 | Both | hypermethylation |
| 13 | 105934921 | 105935097 | Both | hypomethylation |
| 13 | 106426059 | 106426169 | Cancer-specific | hypomethylation |
| 13 | 106762552 | 106762582 | Cancer-specific | hypomethylation |
| 13 | 106762795 | 106762830 | Both | hypomethylation |
| 13 | 106882447 | 106882707 | Cancer-specific | hypomethylation |
| 13 | 106886345 | 106886649 | Both | hypomethylation |
| 13 | 107026171 | 107026185 | Both | hypomethylation |
| 13 | 107027422 | 107027791 | Cancer-specific | hypomethylation |
| 13 | 108520938 | 108521069 | Cancer-specific | hypermethylation |
| 13 | 110960059 | 110960154 | Cancer-specific | hypermethylation |
| 13 | 111773836 | 111773965 | Both | hypomethylation |
| 13 | 111773988 | 111774134 | Cancer-specific | hypomethylation |
| 13 | 112711462 | 112711621 | Cancer-specific | hypermethylation |
| 13 | 112711965 | 112712054 | Cancer-specific | hypermethylation |
| 13 | 112721587 | 112721657 | Cancer-specific | hypermethylation |
| 13 | 112721662 | 112721828 | Cancer-specific | hypermethylation |
| 13 | 112722215 | 112722362 | Cancer-specific | hypermethylation |
| 13 | 112722372 | 112722459 | Cancer-specific | hypermethylation |
| 13 | 112723373 | 112723520 | Cancer-specific | hypermethylation |
| 13 | 112723520 | 112723588 | Cancer-specific | hypermethylation |
| 13 | 112758283 | 112758521 | Cancer-specific | hypermethylation |
| 13 | 112759244 | 112759343 | Cancer-specific | hypermethylation |
| 13 | 112759348 | 112759473 | Cancer-specific | hypermethylation |
| 13 | 112759601 | 112759913 | Cancer-specific | hypermethylation |
| 13 | 20767584 | 20767633 | Cancer-specific | hypermethylation |
| 13 | 20767643 | 20767785 | Cancer-specific | hypermethylation |
| 13 | 21649632 | 21649751 | Cancer-specific | hypermethylation |
| 13 | 21649743 | 21649837 | Cancer-specific | hypermethylation |
| 13 | 25745642 | 25745745 | Cancer-specific | hypermethylation |
| 13 | 26625185 | 26625332 | Cancer-specific | hypermethylation |
| 13 | 26625645 | 26625805 | Cancer-specific | hypermethylation |
| 13 | 26626013 | 26626141 | Cancer-specific | hypermethylation |
| 13 | 27131657 | 27131763 | Cancer-specific | hypermethylation |
| 13 | 28491482 | 28491604 | Cancer-specific | hypermethylation |
| 13 | 28491605 | 28491711 | Cancer-specific | hypermethylation |
| 13 | 28501246 | 28501305 | Cancer-specific | hypermethylation |
| 13 | 28501332 | 28501453 | Both | hypermethylation |
| 13 | 36705324 | 36705482 | Cancer-specific | hypermethylation |
| 13 | 36920251 | 36920524 | Cancer-specific | hypermethylation |
| 13 | 36920649 | 36920778 | Cancer-specific | hypermethylation |
| 13 | 37006192 | 37006399 | Cancer-specific | hypermethylation |
| 13 | 37006396 | 37006562 | Cancer-specific | hypermethylation |
| 13 | 37248171 | 37248430 | Cancer-specific | hypermethylation |
| 13 | 46189786 | 46189874 | Cancer-specific | hypermethylation |
| 13 | 46189876 | 46190014 | Cancer-specific | hypermethylation |
| 13 | 50570135 | 50570392 | Both | hypermethylation |
| 13 | 50570387 | 50570468 | Cancer-specific | hypermethylation |
| 13 | 50706539 | 50706837 | Cancer-specific | hypermethylation |
| 13 | 50706867 | 50707275 | Cancer-specific | hypermethylation |
| 13 | 53420260 | 53420398 | Cancer-specific | hypermethylation |
| 13 | 53420405 | 53420499 | Cancer-specific | hypermethylation |
| 13 | 53422651 | 53422818 | Cancer-specific | hypermethylation |
| 13 | 78493059 | 78493135 | Cancer-specific | hypermethylation |
| 13 | 79161060 | 79161134 | Cancer-specific | hypermethylation |
| 13 | 79176460 | 79176555 | Cancer-specific | hypermethylation |
| 13 | 79176551 | 79176675 | Cancer-specific | hypermethylation |
| 13 | 79177455 | 79177704 | Cancer-specific | hypermethylation |
| 13 | 79177693 | 79177829 | Cancer-specific | hypermethylation |
| 13 | 79177827 | 79177902 | Cancer-specific | hypermethylation |
| 13 | 79179729 | 79179861 | Cancer-specific | hypermethylation |
| 13 | 84453273 | 84453407 | Cancer-specific | hypermethylation |
| 13 | 88324817 | 88324886 | Cancer-specific | hypermethylation |
| 13 | 88324874 | 88324977 | Cancer-specific | hypermethylation |
| 13 | 92051488 | 92051688 | Cancer-specific | hypermethylation |
| 13 | 95655203 | 95655350 | Cancer-specific | hypermethylation |
| 13 | 95655354 | 95655450 | Cancer-specific | hypermethylation |
| 13 | 96743768 | 96743908 | Cancer-specific | hypermethylation |
| 13 | 96743896 | 96744012 | Cancer-specific | hypermethylation |
| 14 | 101341247 | 101341556 | Both | hypomethylation |
| 14 | 101495737 | 101496154 | Cancer-specific | hypomethylation |
| 14 | 101527030 | 101527321 | Both | hypomethylation |
| 14 | 102026577 | 102026680 | Cancer-specific | hypermethylation |
| 14 | 103021869 | 103021959 | Cancer-specific | hypermethylation |
| 14 | 103021948 | 103022112 | Cancer-specific | hypermethylation |
| 14 | 103394779 | 103394924 | Cancer-specific | hypermethylation |
| 14 | 103394912 | 103394968 | Cancer-specific | hypermethylation |
| 14 | 103394971 | 103395026 | Cancer-specific | hypermethylation |
| 14 | 103655299 | 103655431 | Cancer-specific | hypermethylation |
| 14 | 103655421 | 103655550 | Cancer-specific | hypermethylation |
| 14 | 103739927 | 103740040 | Cancer-specific | hypermethylation |
| 14 | 103740058 | 103740143 | Cancer-specific | hypermethylation |
| 14 | 105165062 | 105165230 | Tissue-specific | hypermethylation |
| 14 | 21093050 | 21093114 | Cancer-specific | hypermethylation |
| 14 | 21121439 | 21121503 | Cancer-specific | hypermethylation |
| 14 | 22004872 | 22005021 | Cancer-specific | hypermethylation |
| 14 | 22005029 | 22005174 | Cancer-specific | hypermethylation |
| 14 | 24803800 | 24803910 | Cancer-specific | hypermethylation |
| 14 | 24803910 | 24804055 | Cancer-specific | hypermethylation |
| 14 | 25103723 | 25103882 | Both | hypomethylation |
| 14 | 27065967 | 27066083 | Cancer-specific | hypermethylation |
| 14 | 29235155 | 29235249 | Cancer-specific | hypermethylation |
| 14 | 29237363 | 29237538 | Cancer-specific | hypermethylation |
| 14 | 29237552 | 29237724 | Cancer-specific | hypermethylation |
| 14 | 31344418 | 31344480 | Cancer-specific | hypermethylation |
| 14 | 31344528 | 31344659 | Cancer-specific | hypermethylation |
| 14 | 31464277 | 31464291 | Both | hypermethylation |
| 14 | 31464354 | 31464396 | Cancer-specific | hypermethylation |
| 14 | 36003620 | 36003726 | Cancer-specific | hypermethylation |
| 14 | 36003716 | 36003861 | Cancer-specific | hypermethylation |
| 14 | 36003853 | 36004019 | Cancer-specific | hypermethylation |
| 14 | 36004009 | 36004216 | Cancer-specific | hypermethylation |
| 14 | 36004720 | 36004809 | Cancer-specific | hypermethylation |
| 14 | 36973263 | 36973394 | Cancer-specific | hypermethylation |
| 14 | 36973388 | 36973490 | Cancer-specific | hypermethylation |
| 14 | 36973565 | 36973680 | Cancer-specific | hypermethylation |
| 14 | 36973669 | 36973801 | Cancer-specific | hypermethylation |
| 14 | 36987177 | 36987312 | Cancer-specific | hypermethylation |
| 14 | 36987303 | 36987418 | Cancer-specific | hypermethylation |
| 14 | 37123898 | 37124070 | Cancer-specific | hypermethylation |
| 14 | 37132539 | 37132688 | Cancer-specific | hypermethylation |
| 14 | 37132681 | 37132791 | Cancer-specific | hypermethylation |
| 14 | 38069526 | 38069568 | Both | hypermethylation |
| 14 | 38069632 | 38069735 | Cancer-specific | hypermethylation |
| 14 | 51027788 | 51027888 | Cancer-specific | hypermethylation |
| 14 | 51027879 | 51027982 | Cancer-specific | hypermethylation |
| 14 | 51560371 | 51560561 | Cancer-specific | hypermethylation |
| 14 | 51560558 | 51560807 | Cancer-specific | hypermethylation |
| 14 | 52734149 | 52734401 | Cancer-specific | hypermethylation |
| 14 | 52734390 | 52734504 | Cancer-specific | hypermethylation |
| 14 | 52734518 | 52734626 | Cancer-specific | hypermethylation |
| 14 | 54422805 | 54422898 | Cancer-specific | hypermethylation |
| 14 | 54422905 | 54423040 | Cancer-specific | hypermethylation |
| 14 | 56586502 | 56586547 | Cancer-specific | hypermethylation |
| 14 | 56586562 | 56586649 | Cancer-specific | hypermethylation |
| 14 | 57261146 | 57261263 | Cancer-specific | hypermethylation |
| 14 | 57261286 | 57261380 | Cancer-specific | hypermethylation |
| 14 | 57261480 | 57261709 | Cancer-specific | hypermethylation |
| 14 | 57261776 | 57261893 | Cancer-specific | hypermethylation |
| 14 | 57261926 | 57262245 | Cancer-specific | hypermethylation |
| 14 | 57264801 | 57264930 | Cancer-specific | hypermethylation |
| 14 | 57265261 | 57265387 | Cancer-specific | hypermethylation |
| 14 | 57265454 | 57265524 | Cancer-specific | hypermethylation |
| 14 | 57265526 | 57265670 | Cancer-specific | hypermethylation |
| 14 | 57274563 | 57274703 | Cancer-specific | hypermethylation |
| 14 | 57274697 | 57274836 | Cancer-specific | hypermethylation |
| 14 | 57275159 | 57275515 | Cancer-specific | hypermethylation |
| 14 | 57275508 | 57275713 | Cancer-specific | hypermethylation |
| 14 | 57276177 | 57276305 | Cancer-specific | hypermethylation |
| 14 | 57276305 | 57276463 | Cancer-specific | hypermethylation |
| 14 | 57278173 | 57278389 | Cancer-specific | hypermethylation |
| 14 | 57278630 | 57278766 | Cancer-specific | hypermethylation |
| 14 | 57278757 | 57278853 | Cancer-specific | hypermethylation |
| 14 | 57283473 | 57283554 | Cancer-specific | hypermethylation |
| 14 | 60952475 | 60952561 | Cancer-specific | hypermethylation |
| 14 | 61104474 | 61104594 | Cancer-specific | hypermethylation |
| 14 | 61104600 | 61104702 | Cancer-specific | hypermethylation |
| 14 | 61109200 | 61109463 | Cancer-specific | hypermethylation |
| 14 | 61109483 | 61109792 | Cancer-specific | hypermethylation |
| 14 | 61119153 | 61119221 | Cancer-specific | hypermethylation |
| 14 | 61119211 | 61119337 | Cancer-specific | hypermethylation |
| 14 | 64760725 | 64760898 | Cancer-specific | hypermethylation |
| 14 | 69726146 | 69726258 | Cancer-specific | hypermethylation |
| 14 | 69726247 | 69726378 | Cancer-specific | hypermethylation |
| 14 | 70654482 | 70654586 | Cancer-specific | hypermethylation |
| 14 | 74707240 | 74707350 | Cancer-specific | hypermethylation |
| 14 | 74707353 | 74707474 | Cancer-specific | hypermethylation |
| 14 | 74893075 | 74893190 | Cancer-specific | hypermethylation |
| 14 | 77227513 | 77227598 | Cancer-specific | hypermethylation |
| 14 | 77227608 | 77227676 | Cancer-specific | hypermethylation |
| 14 | 77228628 | 77228697 | Cancer-specific | hypermethylation |
| 14 | 77591546 | 77591632 | Cancer-specific | hypermethylation |
| 14 | 77591624 | 77591780 | Cancer-specific | hypermethylation |
| 14 | 92413579 | 92413775 | Cancer-specific | hypermethylation |
| 14 | 94392650 | 94392911 | Cancer-specific | hypomethylation |
| 14 | 94392904 | 94393120 | Both | hypomethylation |
| 14 | 95234705 | 95234814 | Cancer-specific | hypermethylation |
| 14 | 97059069 | 97059165 | Cancer-specific | hypermethylation |
| 14 | 97059165 | 97059312 | Cancer-specific | hypermethylation |
| 15 | 101591928 | 101592241 | Cancer-specific | hypomethylation |
| 15 | 101593234 | 101593591 | Cancer-specific | hypomethylation |
| 15 | 101595255 | 101595524 | Cancer-specific | hypomethylation |
| 15 | 101601061 | 101601391 | Both | hypomethylation |
| 15 | 33010274 | 33010387 | Cancer-specific | hypermethylation |
| 15 | 34728990 | 34729033 | Cancer-specific | hypermethylation |
| 15 | 41952320 | 41952384 | Cancer-specific | hypermethylation |
| 15 | 41952422 | 41952545 | Cancer-specific | hypermethylation |
| 15 | 45408747 | 45408917 | Cancer-specific | hypermethylation |
| 15 | 45408908 | 45409111 | Cancer-specific | hypermethylation |
| 15 | 45421944 | 45422073 | Cancer-specific | hypermethylation |
| 15 | 45422074 | 45422186 | Cancer-specific | hypermethylation |
| 15 | 58357776 | 58357886 | Cancer-specific | hypermethylation |
| 15 | 58357884 | 58357996 | Cancer-specific | hypermethylation |
| 15 | 60287217 | 60287302 | Cancer-specific | hypermethylation |
| 15 | 60296344 | 60296476 | Cancer-specific | hypermethylation |
| 15 | 60296492 | 60296592 | Cancer-specific | hypermethylation |
| 15 | 60296682 | 60296932 | Cancer-specific | hypermethylation |
| 15 | 63889559 | 63889638 | Both | hypermethylation |
| 15 | 63889704 | 63889844 | Tissue-specific | hypermethylation |
| 15 | 63891694 | 63891865 | Tissue-specific | hypomethylation |
| 15 | 66998859 | 66999124 | Tissue-specific | hypermethylation |
| 15 | 68120906 | 68121016 | Cancer-specific | hypermethylation |
| 15 | 68121436 | 68121689 | Cancer-specific | hypermethylation |
| 15 | 68122023 | 68122173 | Cancer-specific | hypermethylation |
| 15 | 68122171 | 68122257 | Cancer-specific | hypermethylation |
| 15 | 68122447 | 68122581 | Cancer-specific | hypermethylation |
| 15 | 68122652 | 68122794 | Cancer-specific | hypermethylation |
| 15 | 68128644 | 68128729 | Cancer-specific | hypermethylation |
| 15 | 68851857 | 68851905 | Cancer-specific | hypermethylation |
| 15 | 76628048 | 76628073 | Both | hypermethylation |
| 15 | 76628141 | 76628221 | Cancer-specific | hypermethylation |
| 15 | 76629994 | 76630120 | Cancer-specific | hypermethylation |
| 15 | 76630119 | 76630216 | Cancer-specific | hypermethylation |
| 15 | 76630221 | 76630381 | Cancer-specific | hypermethylation |
| 15 | 78912705 | 78912777 | Cancer-specific | hypermethylation |
| 15 | 83316513 | 83317032 | Cancer-specific | hypermethylation |
| 15 | 83776143 | 83776250 | Cancer-specific | hypermethylation |
| 15 | 83776243 | 83776340 | Cancer-specific | hypermethylation |
| 15 | 83952941 | 83953057 | Cancer-specific | hypermethylation |
| 15 | 83953048 | 83953182 | Cancer-specific | hypermethylation |
| 15 | 84116067 | 84116252 | Cancer-specific | hypermethylation |
| 15 | 89284621 | 89284791 | Cancer-specific | hypomethylation |
| 15 | 89284819 | 89284923 | Cancer-specific | hypomethylation |
| 15 | 89911340 | 89911425 | Cancer-specific | hypermethylation |
| 15 | 89914341 | 89914492 | Cancer-specific | hypermethylation |
| 15 | 89914481 | 89914577 | Cancer-specific | hypermethylation |
| 15 | 89921030 | 89921120 | Cancer-specific | hypermethylation |
| 15 | 89921149 | 89921265 | Cancer-specific | hypermethylation |
| 15 | 89951849 | 89951885 | Cancer-specific | hypermethylation |
| 15 | 90039733 | 90039855 | Cancer-specific | hypermethylation |
| 15 | 91499887 | 91499988 | Cancer-specific | hypermethylation |
| 15 | 99193907 | 99194008 | Cancer-specific | hypermethylation |
| 15 | 99194009 | 99194146 | Cancer-specific | hypermethylation |
| 15 | 99250385 | 99250584 | Cancer-specific | hypomethylation |
| 15 | 99391570 | 99391717 | Cancer-specific | hypomethylation |
| 15 | 99512659 | 99512811 | Cancer-specific | hypomethylation |
| 15 | 99512835 | 99512980 | Both | hypomethylation |
| 16 | 10133481 | 10133559 | Cancer-specific | hypermethylation |
| 16 | 19567227 | 19567311 | Cancer-specific | hypermethylation |
| 16 | 19567347 | 19567442 | Cancer-specific | hypermethylation |
| 16 | 19895204 | 19895271 | Cancer-specific | hypermethylation |
| 16 | 21295142 | 21295282 | Cancer-specific | hypermethylation |
| 16 | 22824888 | 22824991 | Cancer-specific | hypermethylation |
| 16 | 22825248 | 22825397 | Cancer-specific | hypermethylation |
| 16 | 22825990 | 22826124 | Cancer-specific | hypermethylation |
| 16 | 230218 | 230372 | Cancer-specific | hypermethylation |
| 16 | 23846777 | 23846975 | Cancer-specific | hypermethylation |
| 16 | 23846979 | 23847099 | Cancer-specific | hypermethylation |
| 16 | 23847290 | 23847399 | Cancer-specific | hypermethylation |
| 16 | 23847397 | 23847514 | Cancer-specific | hypermethylation |
| 16 | 23847506 | 23847682 | Cancer-specific | hypermethylation |
| 16 | 29796567 | 29796717 | Cancer-specific | hypermethylation |
| 16 | 29796736 | 29796805 | Tissue-specific | hypermethylation |
| 16 | 30032458 | 30032550 | Cancer-specific | hypermethylation |
| 16 | 30133076 | 30133129 | Both | hypermethylation |
| 16 | 30133164 | 30133385 | Cancer-specific | hypermethylation |
| 16 | 3068214 | 3068292 | Cancer-specific | hypermethylation |
| 16 | 3068282 | 3068423 | Cancer-specific | hypermethylation |
| 16 | 31488136 | 31488460 | Tissue-specific | hypomethylation |
| 16 | 31549014 | 31549103 | Cancer-specific | hypermethylation |
| 16 | 31549096 | 31549256 | Cancer-specific | hypermethylation |
| 16 | 3239342 | 3239408 | Cancer-specific | hypermethylation |
| 16 | 3239426 | 3239578 | Cancer-specific | hypermethylation |
| 16 | 49315442 | 49315572 | Cancer-specific | hypermethylation |
| 16 | 53407363 | 53407450 | Cancer-specific | hypermethylation |
| 16 | 54325201 | 54325409 | Cancer-specific | hypermethylation |
| 16 | 54325407 | 54325563 | Cancer-specific | hypermethylation |
| 16 | 55090736 | 55090834 | Cancer-specific | hypermethylation |
| 16 | 57653606 | 57653924 | Both | hypomethylation |
| 16 | 58019162 | 58019248 | Cancer-specific | hypermethylation |
| 16 | 58463598 | 58463612 | Cancer-specific | hypomethylation |
| 16 | 58497113 | 58497176 | Cancer-specific | hypermethylation |
| 16 | 58497223 | 58497334 | Cancer-specific | hypermethylation |
| 16 | 58497322 | 58497425 | Cancer-specific | hypermethylation |
| 16 | 58764874 | 58764893 | Both | hypomethylation |
| 16 | 6068982 | 6069141 | Cancer-specific | hypermethylation |
| 16 | 6069811 | 6069895 | Cancer-specific | hypermethylation |
| 16 | 6623638 | 6623760 | Cancer-specific | hypermethylation |
| 16 | 6623770 | 6623863 | Cancer-specific | hypermethylation |
| 16 | 66612843 | 66612923 | Cancer-specific | hypermethylation |
| 16 | 66612925 | 66613081 | Cancer-specific | hypermethylation |
| 16 | 67681058 | 67681192 | Both | hypomethylation |
| 16 | 79623624 | 79623781 | Cancer-specific | hypermethylation |
| 16 | 79623779 | 79623867 | Cancer-specific | hypermethylation |
| 16 | 83981550 | 83981564 | Both | hypomethylation |
| 16 | 83981671 | 83981730 | Both | hypomethylation |
| 16 | 86600259 | 86600348 | Cancer-specific | hypermethylation |
| 16 | 89035541 | 89035617 | Both | hypomethylation |
| 16 | 89044983 | 89045240 | Both | hypomethylation |
| 17 | 12893298 | 12893655 | Both | hypomethylation |
| 17 | 17627462 | 17627563 | Both | hypermethylation |
| 17 | 17627561 | 17627697 | Both | hypermethylation |
| 17 | 17687373 | 17687429 | Cancer-specific | hypermethylation |
| 17 | 17687448 | 17687547 | Both | hypermethylation |
| 17 | 1960868 | 1960993 | Both | hypermethylation |
| 17 | 1960983 | 1961116 | Both | hypermethylation |
| 17 | 1975099 | 1975426 | Tissue-specific | hypermethylation |
| 17 | 26578396 | 26578457 | Cancer-specific | hypermethylation |
| 17 | 26578463 | 26578628 | Cancer-specific | hypermethylation |
| 17 | 26692692 | 26692851 | Both | hypomethylation |
| 17 | 27332623 | 27332688 | Cancer-specific | hypermethylation |
| 17 | 27332683 | 27332769 | Cancer-specific | hypermethylation |
| 17 | 29298058 | 29298155 | Cancer-specific | hypermethylation |
| 17 | 29298177 | 29298307 | Cancer-specific | hypermethylation |
| 17 | 3438807 | 3438943 | Cancer-specific | hypermethylation |
| 17 | 3438932 | 3439018 | Cancer-specific | hypermethylation |
| 17 | 3439009 | 3439052 | Cancer-specific | hypermethylation |
| 17 | 35078541 | 35078675 | Cancer-specific | hypermethylation |
| 17 | 35078677 | 35078777 | Cancer-specific | hypermethylation |
| 17 | 35292403 | 35292502 | Cancer-specific | hypermethylation |
| 17 | 35293806 | 35293937 | Cancer-specific | hypermethylation |
| 17 | 36666091 | 36666220 | Both | hypomethylation |
| 17 | 36666219 | 36666344 | Both | hypermethylation |
| 17 | 37762468 | 37762577 | Cancer-specific | hypermethylation |
| 17 | 37764132 | 37764187 | Cancer-specific | hypermethylation |
| 17 | 39645753 | 39645868 | Cancer-specific | hypomethylation |
| 17 | 39645869 | 39646054 | Cancer-specific | hypomethylation |
| 17 | 41557461 | 41557582 | Both | hypomethylation |
| 17 | 41832741 | 41832880 | Cancer-specific | hypermethylation |
| 17 | 42015362 | 42015433 | Cancer-specific | hypermethylation |
| 17 | 42402757 | 42402859 | Cancer-specific | hypermethylation |
| 17 | 42402866 | 42402981 | Cancer-specific | hypermethylation |
| 17 | 45332048 | 45332135 | Cancer-specific | hypermethylation |
| 17 | 45811303 | 45811438 | Cancer-specific | hypermethylation |
| 17 | 46688882 | 46688969 | Cancer-specific | hypermethylation |
| 17 | 46724146 | 46724210 | Cancer-specific | hypermethylation |
| 17 | 46796793 | 46796872 | Cancer-specific | hypermethylation |
| 17 | 46796868 | 46796987 | Cancer-specific | hypermethylation |
| 17 | 46799919 | 46800045 | Cancer-specific | hypermethylation |
| 17 | 46800567 | 46800687 | Cancer-specific | hypermethylation |
| 17 | 46800692 | 46800752 | Cancer-specific | hypermethylation |
| 17 | 46800970 | 46801068 | Cancer-specific | hypermethylation |
| 17 | 46801075 | 46801176 | Cancer-specific | hypermethylation |
| 17 | 46824757 | 46824834 | Cancer-specific | hypermethylation |
| 17 | 47075168 | 47075288 | Cancer-specific | hypermethylation |
| 17 | 47075296 | 47075395 | Cancer-specific | hypermethylation |
| 17 | 47271096 | 47271175 | Cancer-specific | hypermethylation |
| 17 | 47271178 | 47271237 | Cancer-specific | hypermethylation |
| 17 | 48050433 | 48050654 | Cancer-specific | hypermethylation |
| 17 | 48167089 | 48167196 | Both | hypomethylation |
| 17 | 48167228 | 48167341 | Cancer-specific | hypomethylation |
| 17 | 48284287 | 48284402 | Cancer-specific | hypermethylation |
| 17 | 55970750 | 55970851 | Both | hypermethylation |
| 17 | 55970866 | 55971027 | Tissue-specific | hypomethylation |
| 17 | 58499058 | 58499136 | Cancer-specific | hypermethylation |
| 17 | 59478879 | 59478960 | Cancer-specific | hypermethylation |
| 17 | 59478951 | 59479053 | Cancer-specific | hypermethylation |
| 17 | 59482568 | 59482691 | Both | hypermethylation |
| 17 | 59482683 | 59482806 | Both | hypermethylation |
| 17 | 60784328 | 60784626 | Cancer-specific | hypomethylation |
| 17 | 60816977 | 60817228 | Cancer-specific | hypomethylation |
| 17 | 64830988 | 64831149 | Cancer-specific | hypermethylation |
| 17 | 66195679 | 66195773 | Cancer-specific | hypermethylation |
| 17 | 6941688 | 6941941 | Both | hypomethylation |
| 17 | 70112785 | 70112954 | Cancer-specific | hypermethylation |
| 17 | 70112994 | 70113116 | Cancer-specific | hypermethylation |
| 17 | 72755455 | 72755654 | Both | hypomethylation |
| 17 | 7343964 | 7344057 | Cancer-specific | hypermethylation |
| 17 | 74964969 | 74965031 | Cancer-specific | hypermethylation |
| 17 | 74965588 | 74965688 | Cancer-specific | hypermethylation |
| 17 | 75368793 | 75369098 | Cancer-specific | hypermethylation |
| 17 | 75369093 | 75369202 | Cancer-specific | hypermethylation |
| 17 | 75369197 | 75369344 | Cancer-specific | hypermethylation |
| 17 | 75369475 | 75369598 | Cancer-specific | hypermethylation |
| 17 | 75369586 | 75369755 | Cancer-specific | hypermethylation |
| 17 | 75370162 | 75370398 | Cancer-specific | hypermethylation |
| 17 | 75370414 | 75370669 | Cancer-specific | hypermethylation |
| 17 | 76502951 | 76503091 | Both | hypomethylation |
| 17 | 76503141 | 76503300 | Both | hypomethylation |
| 17 | 76507837 | 76508154 | Cancer-specific | hypomethylation |
| 17 | 78451438 | 78451560 | Cancer-specific | hypermethylation |
| 17 | 78451940 | 78452055 | Cancer-specific | hypermethylation |
| 17 | 78452049 | 78452168 | Cancer-specific | hypermethylation |
| 17 | 79228745 | 79229034 | Both | hypermethylation |
| 17 | 80329846 | 80329971 | Cancer-specific | hypermethylation |
| 17 | 80329972 | 80330089 | Cancer-specific | hypermethylation |
| 17 | 80944045 | 80944241 | Both | hypermethylation |
| 17 | 80944294 | 80944343 | Both | hypermethylation |
| 17 | 8926036 | 8926165 | Cancer-specific | hypermethylation |
| 17 | 8926184 | 8926269 | Cancer-specific | hypermethylation |
| 17 | 915673 | 915856 | Cancer-specific | hypomethylation |
| 17 | 915861 | 915985 | Cancer-specific | hypomethylation |
| 17 | 923721 | 924062 | Both | hypomethylation |
| 17 | 951978 | 952477 | Cancer-specific | hypomethylation |
| 17 | 9929748 | 9929894 | Both | hypomethylation |
| 17 | 9930026 | 9930310 | Both | hypomethylation |
| 18 | 19756766 | 19756886 | Both | hypermethylation |
| 18 | 19756882 | 19757005 | Both | hypermethylation |
| 18 | 22930767 | 22930891 | Cancer-specific | hypermethylation |
| 18 | 22930883 | 22931010 | Cancer-specific | hypermethylation |
| 18 | 2847404 | 2847523 | Cancer-specific | hypermethylation |
| 18 | 28621526 | 28621612 | Cancer-specific | hypermethylation |
| 18 | 28621629 | 28621760 | Cancer-specific | hypermethylation |
| 18 | 28622087 | 28622317 | Cancer-specific | hypermethylation |
| 18 | 2906156 | 2906273 | Cancer-specific | hypermethylation |
| 18 | 2906265 | 2906389 | Cancer-specific | hypermethylation |
| 18 | 31803447 | 31803489 | Cancer-specific | hypermethylation |
| 18 | 35147140 | 35147213 | Cancer-specific | hypermethylation |
| 18 | 3845660 | 3845710 | Both | hypomethylation |
| 18 | 3880450 | 3880687 | Cancer-specific | hypomethylation |
| 18 | 53257205 | 53257347 | Cancer-specific | hypermethylation |
| 18 | 55106177 | 55106317 | Cancer-specific | hypermethylation |
| 18 | 55106330 | 55106403 | Cancer-specific | hypermethylation |
| 18 | 56117208 | 56117368 | Both | hypomethylation |
| 18 | 56935078 | 56935170 | Cancer-specific | hypermethylation |
| 18 | 59559968 | 59560070 | Cancer-specific | hypermethylation |
| 18 | 61616444 | 61616523 | Cancer-specific | hypermethylation |
| 18 | 61616515 | 61616685 | Cancer-specific | hypermethylation |
| 18 | 7117000 | 7117092 | Cancer-specific | hypermethylation |
| 18 | 7118062 | 7118197 | Cancer-specific | hypermethylation |
| 18 | 74962245 | 74962381 | Cancer-specific | hypermethylation |
| 18 | 76740580 | 76740698 | Cancer-specific | hypermethylation |
| 18 | 76740702 | 76740799 | Cancer-specific | hypermethylation |
| 18 | 77336143 | 77336331 | Cancer-specific | hypomethylation |
| 18 | 77336341 | 77336528 | Cancer-specific | hypomethylation |
| 18 | 909079 | 909225 | Cancer-specific | hypermethylation |
| 18 | 9333923 | 9333981 | Cancer-specific | hypermethylation |
| 19 | 10047642 | 10047766 | Cancer-specific | hypermethylation |
| 19 | 10047767 | 10047831 | Cancer-specific | hypermethylation |
| 19 | 10398235 | 10398341 | Cancer-specific | hypermethylation |
| 19 | 11347352 | 11347625 | Both | hypomethylation |
| 19 | 12203298 | 12203393 | Cancer-specific | hypermethylation |
| 19 | 12203399 | 12203473 | Cancer-specific | hypermethylation |
| 19 | 12306176 | 12306237 | Cancer-specific | hypermethylation |
| 19 | 12306242 | 12306310 | Cancer-specific | hypermethylation |
| 19 | 12758592 | 12758688 | Cancer-specific | hypermethylation |
| 19 | 12758676 | 12758760 | Cancer-specific | hypermethylation |
| 19 | 12952085 | 12952254 | Cancer-specific | hypermethylation |
| 19 | 12978306 | 12978426 | Both | hypermethylation |
| 19 | 12978425 | 12978509 | Cancer-specific | hypermethylation |
| 19 | 12978597 | 12978671 | Cancer-specific | hypermethylation |
| 19 | 12978670 | 12978812 | Cancer-specific | hypermethylation |
| 19 | 13208536 | 13208866 | Cancer-specific | hypermethylation |
| 19 | 13983496 | 13983630 | Cancer-specific | hypermethylation |
| 19 | 15342628 | 15342674 | Cancer-specific | hypermethylation |
| 19 | 15342709 | 15342776 | Cancer-specific | hypermethylation |
| 19 | 15342766 | 15342875 | Cancer-specific | hypermethylation |
| 19 | 15343938 | 15344105 | Cancer-specific | hypermethylation |
| 19 | 15344100 | 15344275 | Cancer-specific | hypermethylation |
| 19 | 15344286 | 15344610 | Cancer-specific | hypermethylation |
| 19 | 15662168 | 15662297 | Both | hypermethylation |
| 19 | 15662285 | 15662386 | Cancer-specific | hypermethylation |
| 19 | 19072768 | 19072906 | Cancer-specific | hypermethylation |
| 19 | 20277990 | 20278066 | Cancer-specific | hypermethylation |
| 19 | 20348904 | 20349032 | Cancer-specific | hypermethylation |
| 19 | 22034291 | 22034454 | Cancer-specific | hypermethylation |
| 19 | 22034470 | 22034545 | Cancer-specific | hypermethylation |
| 19 | 22610593 | 22610680 | Cancer-specific | hypermethylation |
| 19 | 22610695 | 22610832 | Cancer-specific | hypermethylation |
| 19 | 30017716 | 30017817 | Cancer-specific | hypermethylation |
| 19 | 31839555 | 31839686 | Cancer-specific | hypermethylation |
| 19 | 3369471 | 3369536 | Cancer-specific | hypermethylation |
| 19 | 3369533 | 3369647 | Cancer-specific | hypermethylation |
| 19 | 35395932 | 35396036 | Cancer-specific | hypermethylation |
| 19 | 35629975 | 35630164 | Both | hypermethylation |
| 19 | 36347908 | 36348161 | Cancer-specific | hypermethylation |
| 19 | 36523808 | 36523893 | Cancer-specific | hypermethylation |
| 19 | 36523900 | 36524042 | Cancer-specific | hypermethylation |
| 19 | 36736166 | 36736325 | Cancer-specific | hypermethylation |
| 19 | 36736328 | 36736523 | Cancer-specific | hypermethylation |
| 19 | 36909286 | 36909425 | Cancer-specific | hypermethylation |
| 19 | 36909436 | 36909567 | Cancer-specific | hypermethylation |
| 19 | 37288167 | 37288234 | Cancer-specific | hypermethylation |
| 19 | 37464295 | 37464554 | Cancer-specific | hypermethylation |
| 19 | 37464562 | 37464758 | Cancer-specific | hypermethylation |
| 19 | 3786124 | 3786253 | Cancer-specific | hypermethylation |
| 19 | 3786245 | 3786364 | Cancer-specific | hypermethylation |
| 19 | 38182824 | 38183152 | Cancer-specific | hypermethylation |
| 19 | 38183157 | 38183425 | Cancer-specific | hypermethylation |
| 19 | 38308256 | 38308345 | Cancer-specific | hypermethylation |
| 19 | 3869050 | 3869181 | Cancer-specific | hypermethylation |
| 19 | 38700361 | 38700514 | Cancer-specific | hypermethylation |
| 19 | 38700523 | 38700698 | Cancer-specific | hypermethylation |
| 19 | 38747147 | 38747208 | Cancer-specific | hypermethylation |
| 19 | 38747210 | 38747326 | Cancer-specific | hypermethylation |
| 19 | 4304439 | 4304612 | Cancer-specific | hypermethylation |
| 19 | 4369535 | 4369598 | Both | hypermethylation |
| 19 | 4369683 | 4369824 | Both | hypermethylation |
| 19 | 44203464 | 44203712 | Cancer-specific | hypermethylation |
| 19 | 44203703 | 44204032 | Cancer-specific | hypermethylation |
| 19 | 48918005 | 48918224 | Tissue-specific | hypomethylation |
| 19 | 49127388 | 49127482 | Cancer-specific | hypermethylation |
| 19 | 49127476 | 49127610 | Cancer-specific | hypermethylation |
| 19 | 49646046 | 49646206 | Cancer-specific | hypermethylation |
| 19 | 49646197 | 49646367 | Cancer-specific | hypermethylation |
| 19 | 50004379 | 50004487 | Both | hypermethylation |
| 19 | 50004481 | 50004632 | Both | hypermethylation |
| 19 | 50194655 | 50194784 | Cancer-specific | hypermethylation |
| 19 | 50316216 | 50316285 | Cancer-specific | hypermethylation |
| 19 | 50316299 | 50316430 | Cancer-specific | hypermethylation |
| 19 | 50836268 | 50836379 | Cancer-specific | hypermethylation |
| 19 | 511188 | 511359 | Both | hypermethylation |
| 19 | 51228179 | 51228333 | Cancer-specific | hypermethylation |
| 19 | 51228321 | 51228482 | Cancer-specific | hypermethylation |
| 19 | 51228474 | 51228783 | Cancer-specific | hypermethylation |
| 19 | 51321182 | 51321299 | Cancer-specific | hypermethylation |
| 19 | 52391071 | 52391173 | Cancer-specific | hypermethylation |
| 19 | 52391179 | 52391274 | Cancer-specific | hypermethylation |
| 19 | 52873144 | 52873235 | Cancer-specific | hypermethylation |
| 19 | 54483334 | 54483437 | Cancer-specific | hypermethylation |
| 19 | 54483440 | 54483665 | Cancer-specific | hypermethylation |
| 19 | 54485370 | 54485637 | Cancer-specific | hypermethylation |
| 19 | 54485629 | 54485755 | Cancer-specific | hypermethylation |
| 19 | 54485749 | 54485852 | Cancer-specific | hypermethylation |
| 19 | 54668449 | 54668572 | Cancer-specific | hypermethylation |
| 19 | 54668583 | 54668723 | Cancer-specific | hypermethylation |
| 19 | 55866298 | 55866535 | Cancer-specific | hypermethylation |
| 19 | 56160107 | 56160138 | Both | hypermethylation |
| 19 | 57018821 | 57019106 | Cancer-specific | hypermethylation |
| 19 | 57049669 | 57049784 | Cancer-specific | hypermethylation |
| 19 | 57049779 | 57049896 | Cancer-specific | hypermethylation |
| 19 | 57683764 | 57683813 | Cancer-specific | hypermethylation |
| 19 | 57683826 | 57683892 | Cancer-specific | hypermethylation |
| 19 | 58220387 | 58220579 | Cancer-specific | hypermethylation |
| 19 | 58220588 | 58220844 | Cancer-specific | hypermethylation |
| 19 | 58399858 | 58399995 | Cancer-specific | hypermethylation |
| 19 | 58400006 | 58400086 | Cancer-specific | hypermethylation |
| 19 | 58545023 | 58545260 | Cancer-specific | hypermethylation |
| 19 | 58545251 | 58545409 | Cancer-specific | hypermethylation |
| 19 | 58629847 | 58629944 | Cancer-specific | hypermethylation |
| 19 | 58629960 | 58630096 | Cancer-specific | hypermethylation |
| 19 | 58951308 | 58951435 | Cancer-specific | hypermethylation |
| 19 | 59074360 | 59074460 | Cancer-specific | hypermethylation |
| 19 | 59074453 | 59074600 | Cancer-specific | hypermethylation |
| 19 | 6463985 | 6464110 | Cancer-specific | hypermethylation |
| 19 | 7794850 | 7794959 | Cancer-specific | hypermethylation |
| 19 | 8115244 | 8115369 | Cancer-specific | hypermethylation |
| 19 | 8115391 | 8115487 | Cancer-specific | hypermethylation |
| 1 | 107683448 | 107683615 | Cancer-specific | hypermethylation |
| 1 | 108508427 | 108508655 | Cancer-specific | hypermethylation |
| 1 | 109203525 | 109203661 | Cancer-specific | hypermethylation |
| 1 | 109203662 | 109203732 | Cancer-specific | hypermethylation |
| 1 | 109656173 | 109656284 | Cancer-specific | hypermethylation |
| 1 | 109656831 | 109656943 | Cancer-specific | hypermethylation |
| 1 | 109656959 | 109657042 | Cancer-specific | hypermethylation |
| 1 | 110610774 | 110610931 | Cancer-specific | hypermethylation |
| 1 | 110610921 | 110611026 | Cancer-specific | hypermethylation |
| 1 | 110611705 | 110611865 | Cancer-specific | hypermethylation |
| 1 | 110611874 | 110611944 | Cancer-specific | hypermethylation |
| 1 | 110672956 | 110673097 | Cancer-specific | hypermethylation |
| 1 | 111217586 | 111217690 | Cancer-specific | hypermethylation |
| 1 | 111217680 | 111217822 | Cancer-specific | hypermethylation |
| 1 | 115632478 | 115632590 | Cancer-specific | hypermethylation |
| 1 | 115632616 | 115632700 | Cancer-specific | hypermethylation |
| 1 | 115880224 | 115880324 | Cancer-specific | hypermethylation |
| 1 | 115880744 | 115880847 | Cancer-specific | hypermethylation |
| 1 | 115880843 | 115880988 | Cancer-specific | hypermethylation |
| 1 | 119526776 | 119527015 | Cancer-specific | hypermethylation |
| 1 | 119527027 | 119527483 | Cancer-specific | hypermethylation |
| 1 | 119527782 | 119527967 | Cancer-specific | hypermethylation |
| 1 | 119528009 | 119528148 | Both | hypermethylation |
| 1 | 119528341 | 119528686 | Cancer-specific | hypermethylation |
| 1 | 119528681 | 119528955 | Cancer-specific | hypermethylation |
| 1 | 119530484 | 119530607 | Cancer-specific | hypermethylation |
| 1 | 119530599 | 119530718 | Cancer-specific | hypermethylation |
| 1 | 119530842 | 119531052 | Cancer-specific | hypermethylation |
| 1 | 119531071 | 119531247 | Cancer-specific | hypermethylation |
| 1 | 119531825 | 119532123 | Cancer-specific | hypermethylation |
| 1 | 119532135 | 119532415 | Cancer-specific | hypermethylation |
| 1 | 119543209 | 119543338 | Cancer-specific | hypermethylation |
| 1 | 119543329 | 119543460 | Cancer-specific | hypermethylation |
| 1 | 119550679 | 119550897 | Cancer-specific | hypermethylation |
| 1 | 119550910 | 119551095 | Cancer-specific | hypermethylation |
| 1 | 14027044 | 14027120 | Cancer-specific | hypermethylation |
| 1 | 145075431 | 145075561 | Cancer-specific | hypermethylation |
| 1 | 145075552 | 145075657 | Cancer-specific | hypermethylation |
| 1 | 147752709 | 147752830 | Cancer-specific | hypermethylation |
| 1 | 147752853 | 147752944 | Cancer-specific | hypermethylation |
| 1 | 147775459 | 147775578 | Cancer-specific | hypermethylation |
| 1 | 147775568 | 147775728 | Cancer-specific | hypermethylation |
| 1 | 148193250 | 148193322 | Cancer-specific | hypermethylation |
| 1 | 148193320 | 148193501 | Cancer-specific | hypermethylation |
| 1 | 14925071 | 14925142 | Cancer-specific | hypermethylation |
| 1 | 151694170 | 151694399 | Cancer-specific | hypermethylation |
| 1 | 156391183 | 156391313 | Cancer-specific | hypermethylation |
| 1 | 156391303 | 156391420 | Cancer-specific | hypermethylation |
| 1 | 156406127 | 156406228 | Cancer-specific | hypermethylation |
| 1 | 156815180 | 156815281 | Both | hypermethylation |
| 1 | 157895628 | 157895961 | Cancer-specific | hypomethylation |
| 1 | 158151241 | 158151375 | Both | hypermethylation |
| 1 | 158151368 | 158151484 | Both | hypermethylation |
| 1 | 160040427 | 160040551 | Cancer-specific | hypermethylation |
| 1 | 160040549 | 160040667 | Cancer-specific | hypermethylation |
| 1 | 161275464 | 161275587 | Cancer-specific | hypermethylation |
| 1 | 161275586 | 161275671 | Cancer-specific | hypermethylation |
| 1 | 161276102 | 161276211 | Cancer-specific | hypermethylation |
| 1 | 161276209 | 161276324 | Both | hypermethylation |
| 1 | 162255004 | 162255046 | Both | hypomethylation |
| 1 | 162255157 | 162255191 | Both | hypomethylation |
| 1 | 165323590 | 165323712 | Cancer-specific | hypermethylation |
| 1 | 165323702 | 165323817 | Cancer-specific | hypermethylation |
| 1 | 166134175 | 166134441 | Cancer-specific | hypermethylation |
| 1 | 166134506 | 166134648 | Cancer-specific | hypermethylation |
| 1 | 166135186 | 166135274 | Cancer-specific | hypermethylation |
| 1 | 166853472 | 166853597 | Cancer-specific | hypermethylation |
| 1 | 166853603 | 166853703 | Cancer-specific | hypermethylation |
| 1 | 166916640 | 166917029 | Cancer-specific | hypermethylation |
| 1 | 167884588 | 167884602 | Cancer-specific | hypomethylation |
| 1 | 169396531 | 169396738 | Cancer-specific | hypermethylation |
| 1 | 17026707 | 17026824 | Cancer-specific | hypermethylation |
| 1 | 17026864 | 17026931 | Cancer-specific | hypermethylation |
| 1 | 170629382 | 170629622 | Cancer-specific | hypermethylation |
| 1 | 170629650 | 170629754 | Cancer-specific | hypermethylation |
| 1 | 170629945 | 170630196 | Cancer-specific | hypermethylation |
| 1 | 170630190 | 170630404 | Cancer-specific | hypermethylation |
| 1 | 170630450 | 170630725 | Cancer-specific | hypermethylation |
| 1 | 171059928 | 171060110 | Both | hypomethylation |
| 1 | 171810651 | 171811114 | Cancer-specific | hypermethylation |
| 1 | 171811192 | 171811331 | Cancer-specific | hypermethylation |
| 1 | 171811378 | 171811636 | Cancer-specific | hypermethylation |
| 1 | 177140114 | 177140279 | Cancer-specific | hypermethylation |
| 1 | 179544949 | 179545080 | Cancer-specific | hypermethylation |
| 1 | 179545159 | 179545407 | Cancer-specific | hypermethylation |
| 1 | 179560979 | 179561231 | Cancer-specific | hypermethylation |
| 1 | 180205246 | 180205287 | Cancer-specific | hypermethylation |
| 1 | 183386717 | 183386849 | Cancer-specific | hypermethylation |
| 1 | 184006008 | 184006102 | Cancer-specific | hypermethylation |
| 1 | 186649212 | 186649391 | Cancer-specific | hypermethylation |
| 1 | 186649386 | 186649454 | Cancer-specific | hypermethylation |
| 1 | 190447225 | 190447340 | Cancer-specific | hypermethylation |
| 1 | 1935167 | 1935283 | Cancer-specific | hypermethylation |
| 1 | 1935365 | 1935568 | Cancer-specific | hypermethylation |
| 1 | 197879655 | 197879748 | Cancer-specific | hypermethylation |
| 1 | 197879759 | 197879853 | Cancer-specific | hypermethylation |
| 1 | 197882720 | 197882805 | Both | hypermethylation |
| 1 | 197882829 | 197882955 | Both | hypermethylation |
| 1 | 197887896 | 197887933 | Cancer-specific | hypermethylation |
| 1 | 197887948 | 197888075 | Cancer-specific | hypermethylation |
| 1 | 197888783 | 197888854 | Cancer-specific | hypermethylation |
| 1 | 197888873 | 197889005 | Both | hypermethylation |
| 1 | 203598465 | 203598580 | Cancer-specific | hypermethylation |
| 1 | 203598581 | 203598681 | Both | hypermethylation |
| 1 | 204797604 | 204797685 | Cancer-specific | hypermethylation |
| 1 | 205399688 | 205399757 | Cancer-specific | hypermethylation |
| 1 | 205819172 | 205819270 | Cancer-specific | hypermethylation |
| 1 | 205819286 | 205819430 | Cancer-specific | hypermethylation |
| 1 | 206223925 | 206224103 | Cancer-specific | hypermethylation |
| 1 | 20669760 | 20669912 | Cancer-specific | hypermethylation |
| 1 | 20669907 | 20670002 | Cancer-specific | hypermethylation |
| 1 | 208084298 | 208084391 | Cancer-specific | hypermethylation |
| 1 | 209605627 | 209605774 | Both | hypomethylation |
| 1 | 209605788 | 209605944 | Both | hypomethylation |
| 1 | 209979234 | 209979352 | Cancer-specific | hypermethylation |
| 1 | 213123583 | 213123758 | Cancer-specific | hypermethylation |
| 1 | 213123774 | 213123972 | Cancer-specific | hypermethylation |
| 1 | 213124418 | 213124811 | Cancer-specific | hypermethylation |
| 1 | 216897191 | 216897239 | Cancer-specific | hypermethylation |
| 1 | 217312919 | 217313168 | Cancer-specific | hypermethylation |
| 1 | 217313159 | 217313302 | Cancer-specific | hypermethylation |
| 1 | 218518461 | 218518572 | Cancer-specific | hypermethylation |
| 1 | 220700762 | 220700873 | Cancer-specific | hypermethylation |
| 1 | 22140893 | 22141021 | Cancer-specific | hypermethylation |
| 1 | 22141017 | 22141139 | Cancer-specific | hypermethylation |
| 1 | 224804389 | 224804523 | Cancer-specific | hypermethylation |
| 1 | 226187491 | 226187585 | Cancer-specific | hypermethylation |
| 1 | 228645190 | 228645324 | Cancer-specific | hypermethylation |
| 1 | 228645328 | 228645444 | Cancer-specific | hypermethylation |
| 1 | 228645446 | 228645564 | Cancer-specific | hypermethylation |
| 1 | 228645583 | 228645810 | Cancer-specific | hypermethylation |
| 1 | 228646834 | 228646960 | Cancer-specific | hypermethylation |
| 1 | 228651581 | 228651626 | Cancer-specific | hypermethylation |
| 1 | 228651649 | 228651791 | Cancer-specific | hypermethylation |
| 1 | 229543002 | 229543080 | Cancer-specific | hypermethylation |
| 1 | 229543072 | 229543228 | Cancer-specific | hypermethylation |
| 1 | 229543437 | 229543493 | Cancer-specific | hypermethylation |
| 1 | 229543499 | 229543610 | Cancer-specific | hypermethylation |
| 1 | 231298644 | 231298765 | Cancer-specific | hypermethylation |
| 1 | 234348629 | 234348659 | Cancer-specific | hypermethylation |
| 1 | 234658419 | 234658539 | Both | hypomethylation |
| 1 | 2350029 | 2350356 | Both | hypomethylation |
| 1 | 235813962 | 235814072 | Cancer-specific | hypermethylation |
| 1 | 237206029 | 237206122 | Cancer-specific | hypermethylation |
| 1 | 237206439 | 237206541 | Cancer-specific | hypermethylation |
| 1 | 237206544 | 237206650 | Cancer-specific | hypermethylation |
| 1 | 240161139 | 240161256 | Cancer-specific | hypermethylation |
| 1 | 240161253 | 240161372 | Cancer-specific | hypermethylation |
| 1 | 241520172 | 241520320 | Cancer-specific | hypermethylation |
| 1 | 241520309 | 241520408 | Cancer-specific | hypermethylation |
| 1 | 241520679 | 241520728 | Cancer-specific | hypermethylation |
| 1 | 243646279 | 243646487 | Cancer-specific | hypermethylation |
| 1 | 243646478 | 243646666 | Cancer-specific | hypermethylation |
| 1 | 247921477 | 247921679 | Both | hypomethylation |
| 1 | 25257035 | 25257138 | Cancer-specific | hypermethylation |
| 1 | 2805030 | 2805159 | Both | hypomethylation |
| 1 | 2805213 | 2805392 | Both | hypomethylation |
| 1 | 2918601 | 2918823 | Both | hypomethylation |
| 1 | 29448916 | 29449049 | Cancer-specific | hypermethylation |
| 1 | 29449041 | 29449162 | Cancer-specific | hypermethylation |
| 1 | 3159445 | 3159683 | Both | hypomethylation |
| 1 | 3159700 | 3159824 | Both | hypomethylation |
| 1 | 3184493 | 3184686 | Both | hypomethylation |
| 1 | 3184676 | 3184945 | Both | hypomethylation |
| 1 | 32226299 | 32226412 | Cancer-specific | hypermethylation |
| 1 | 32237990 | 32238066 | Cancer-specific | hypermethylation |
| 1 | 3228859 | 3229082 | Cancer-specific | hypomethylation |
| 1 | 3229107 | 3229328 | Cancer-specific | hypomethylation |
| 1 | 32930188 | 32930264 | Cancer-specific | hypermethylation |
| 1 | 35258341 | 35258562 | Cancer-specific | hypermethylation |
| 1 | 35351062 | 35351206 | Cancer-specific | hypermethylation |
| 1 | 35351208 | 35351307 | Cancer-specific | hypermethylation |
| 1 | 38100689 | 38100739 | Cancer-specific | hypermethylation |
| 1 | 38219663 | 38219866 | Cancer-specific | hypermethylation |
| 1 | 38219879 | 38220097 | Cancer-specific | hypermethylation |
| 1 | 38510808 | 38511060 | Cancer-specific | hypermethylation |
| 1 | 42846816 | 42846860 | Cancer-specific | hypermethylation |
| 1 | 42846863 | 42846994 | Cancer-specific | hypermethylation |
| 1 | 43251158 | 43251242 | Both | hypermethylation |
| 1 | 43251235 | 43251338 | Cancer-specific | hypermethylation |
| 1 | 44873108 | 44873242 | Cancer-specific | hypermethylation |
| 1 | 44873233 | 44873355 | Cancer-specific | hypermethylation |
| 1 | 44883585 | 44883824 | Cancer-specific | hypermethylation |
| 1 | 45251935 | 45252026 | Cancer-specific | hypermethylation |
| 1 | 45252021 | 45252145 | Cancer-specific | hypermethylation |
| 1 | 46632574 | 46632708 | Cancer-specific | hypermethylation |
| 1 | 46632715 | 46632818 | Cancer-specific | hypermethylation |
| 1 | 46913947 | 46914040 | Cancer-specific | hypermethylation |
| 1 | 46951195 | 46951427 | Cancer-specific | hypermethylation |
| 1 | 46951418 | 46951503 | Cancer-specific | hypermethylation |
| 1 | 46951506 | 46951598 | Cancer-specific | hypermethylation |
| 1 | 46956369 | 46956517 | Cancer-specific | hypermethylation |
| 1 | 46956513 | 46956596 | Cancer-specific | hypermethylation |
| 1 | 47696666 | 47696795 | Cancer-specific | hypermethylation |
| 1 | 47696784 | 47696910 | Cancer-specific | hypermethylation |
| 1 | 47697831 | 47698203 | Cancer-specific | hypermethylation |
| 1 | 47882141 | 47882252 | Cancer-specific | hypermethylation |
| 1 | 47882243 | 47882431 | Cancer-specific | hypermethylation |
| 1 | 47904952 | 47905140 | Both | hypermethylation |
| 1 | 47905128 | 47905331 | Both | hypermethylation |
| 1 | 47909818 | 47910153 | Cancer-specific | hypermethylation |
| 1 | 47910174 | 47910497 | Cancer-specific | hypermethylation |
| 1 | 47911329 | 47911370 | Cancer-specific | hypermethylation |
| 1 | 47911389 | 47911518 | Cancer-specific | hypermethylation |
| 1 | 47999042 | 47999283 | Cancer-specific | hypermethylation |
| 1 | 48937298 | 48937430 | Cancer-specific | hypermethylation |
| 1 | 50881951 | 50882206 | Cancer-specific | hypermethylation |
| 1 | 50886822 | 50886956 | Cancer-specific | hypermethylation |
| 1 | 50886962 | 50887110 | Cancer-specific | hypermethylation |
| 1 | 50893060 | 50893137 | Cancer-specific | hypermethylation |
| 1 | 50893640 | 50893751 | Cancer-specific | hypermethylation |
| 1 | 57888138 | 57888303 | Cancer-specific | hypermethylation |
| 1 | 62660614 | 62660758 | Cancer-specific | hypermethylation |
| 1 | 62660763 | 62660868 | Cancer-specific | hypermethylation |
| 1 | 63785500 | 63785625 | Cancer-specific | hypermethylation |
| 1 | 63785747 | 63785981 | Cancer-specific | hypermethylation |
| 1 | 63785973 | 63786203 | Cancer-specific | hypermethylation |
| 1 | 63787063 | 63787161 | Cancer-specific | hypermethylation |
| 1 | 63787163 | 63787295 | Cancer-specific | hypermethylation |
| 1 | 63791874 | 63791938 | Cancer-specific | hypermethylation |
| 1 | 63792570 | 63792690 | Cancer-specific | hypermethylation |
| 1 | 63795821 | 63795941 | Cancer-specific | hypermethylation |
| 1 | 6514774 | 6515019 | Both | hypermethylation |
| 1 | 6515286 | 6515416 | Both | hypermethylation |
| 1 | 6515408 | 6515526 | Both | hypermethylation |
| 1 | 65731518 | 65731644 | Cancer-specific | hypermethylation |
| 1 | 65731635 | 65731855 | Cancer-specific | hypermethylation |
| 1 | 66258039 | 66258141 | Cancer-specific | hypermethylation |
| 1 | 67217974 | 67218111 | Cancer-specific | hypermethylation |
| 1 | 67218128 | 67218217 | Cancer-specific | hypermethylation |
| 1 | 76082610 | 76082697 | Cancer-specific | hypermethylation |
| 1 | 76082717 | 76082798 | Cancer-specific | hypermethylation |
| 1 | 77334262 | 77334386 | Cancer-specific | hypermethylation |
| 1 | 85463250 | 85463350 | Cancer-specific | hypermethylation |
| 1 | 85463353 | 85463487 | Cancer-specific | hypermethylation |
| 1 | 85913343 | 85913573 | Both | hypermethylation |
| 1 | 91172211 | 91172295 | Cancer-specific | hypermethylation |
| 1 | 91172590 | 91172670 | Cancer-specific | hypermethylation |
| 1 | 91176619 | 91176814 | Cancer-specific | hypermethylation |
| 1 | 91182975 | 91183058 | Cancer-specific | hypermethylation |
| 1 | 91183049 | 91183134 | Cancer-specific | hypermethylation |
| 1 | 91183405 | 91183646 | Cancer-specific | hypermethylation |
| 1 | 91184954 | 91185133 | Cancer-specific | hypermethylation |
| 1 | 91185303 | 91185448 | Cancer-specific | hypermethylation |
| 1 | 91185446 | 91185526 | Cancer-specific | hypermethylation |
| 1 | 91190564 | 91190676 | Cancer-specific | hypermethylation |
| 1 | 91192353 | 91192479 | Cancer-specific | hypermethylation |
| 1 | 91192473 | 91192564 | Cancer-specific | hypermethylation |
| 1 | 91194548 | 91194603 | Cancer-specific | hypermethylation |
| 1 | 91194667 | 91194840 | Cancer-specific | hypermethylation |
| 1 | 91195041 | 91195188 | Cancer-specific | hypermethylation |
| 1 | 91195176 | 91195279 | Cancer-specific | hypermethylation |
| 1 | 9258713 | 9258874 | Cancer-specific | hypermethylation |
| 1 | 9258874 | 9258962 | Cancer-specific | hypermethylation |
| 1 | 98510741 | 98510793 | Both | hypermethylation |
| 1 | 99729750 | 99729822 | Cancer-specific | hypermethylation |
| 20 | 17207269 | 17207350 | Cancer-specific | hypermethylation |
| 20 | 17209490 | 17209557 | Cancer-specific | hypermethylation |
| 20 | 1784160 | 1784251 | Cancer-specific | hypermethylation |
| 20 | 1784259 | 1784392 | Cancer-specific | hypermethylation |
| 20 | 20347581 | 20347718 | Cancer-specific | hypermethylation |
| 20 | 20349041 | 20349136 | Cancer-specific | hypermethylation |
| 20 | 20349131 | 20349211 | Cancer-specific | hypermethylation |
| 20 | 21081650 | 21081879 | Cancer-specific | hypermethylation |
| 20 | 21081875 | 21082019 | Cancer-specific | hypermethylation |
| 20 | 21082179 | 21082269 | Cancer-specific | hypermethylation |
| 20 | 21082265 | 21082384 | Cancer-specific | hypermethylation |
| 20 | 21377922 | 21378127 | Cancer-specific | hypermethylation |
| 20 | 21378131 | 21378300 | Cancer-specific | hypermethylation |
| 20 | 21496381 | 21496486 | Cancer-specific | hypermethylation |
| 20 | 21501684 | 21501778 | Cancer-specific | hypermethylation |
| 20 | 23029165 | 23029305 | Cancer-specific | hypermethylation |
| 20 | 23029302 | 23029410 | Cancer-specific | hypermethylation |
| 20 | 25062320 | 25062574 | Cancer-specific | hypermethylation |
| 20 | 25062665 | 25062784 | Cancer-specific | hypermethylation |
| 20 | 25062812 | 25062934 | Cancer-specific | hypermethylation |
| 20 | 25065087 | 25065230 | Cancer-specific | hypermethylation |
| 20 | 2781161 | 2781294 | Cancer-specific | hypermethylation |
| 20 | 2781283 | 2781564 | Cancer-specific | hypermethylation |
| 20 | 3052571 | 3052819 | Cancer-specific | hypermethylation |
| 20 | 34189298 | 34189454 | Cancer-specific | hypermethylation |
| 20 | 37434138 | 37434269 | Cancer-specific | hypermethylation |
| 20 | 37434285 | 37434383 | Cancer-specific | hypermethylation |
| 20 | 39995530 | 39995601 | Cancer-specific | hypermethylation |
| 20 | 39995593 | 39995772 | Cancer-specific | hypermethylation |
| 20 | 44452830 | 44452922 | Cancer-specific | hypermethylation |
| 20 | 44540416 | 44540520 | Cancer-specific | hypermethylation |
| 20 | 47299144 | 47299430 | Both | hypomethylation |
| 20 | 47303185 | 47303204 | Cancer-specific | hypomethylation |
| 20 | 50721674 | 50721808 | Cancer-specific | hypermethylation |
| 20 | 50721823 | 50721918 | Cancer-specific | hypermethylation |
| 20 | 52276823 | 52276907 | Cancer-specific | hypermethylation |
| 20 | 52276928 | 52277057 | Cancer-specific | hypermethylation |
| 20 | 52790117 | 52790259 | Cancer-specific | hypermethylation |
| 20 | 56803380 | 56803477 | Cancer-specific | hypermethylation |
| 20 | 57465548 | 57465698 | Cancer-specific | hypermethylation |
| 20 | 57465688 | 57465789 | Cancer-specific | hypermethylation |
| 20 | 57766261 | 57766339 | Cancer-specific | hypomethylation |
| 20 | 57766390 | 57766599 | Cancer-specific | hypomethylation |
| 20 | 57875311 | 57875463 | Cancer-specific | hypermethylation |
| 20 | 6033206 | 6033326 | Cancer-specific | hypermethylation |
| 20 | 60753888 | 60754185 | Both | hypomethylation |
| 20 | 61560331 | 61560514 | Cancer-specific | hypermethylation |
| 20 | 61560513 | 61560646 | Cancer-specific | hypermethylation |
| 20 | 61560639 | 61560735 | Cancer-specific | hypermethylation |
| 20 | 61636246 | 61636297 | Cancer-specific | hypermethylation |
| 20 | 61638136 | 61638262 | Cancer-specific | hypermethylation |
| 20 | 61809440 | 61810094 | Cancer-specific | hypermethylation |
| 20 | 62150939 | 62151076 | Cancer-specific | hypermethylation |
| 20 | 62185275 | 62185424 | Cancer-specific | hypermethylation |
| 20 | 62461416 | 62461492 | Cancer-specific | hypermethylation |
| 20 | 62461507 | 62461663 | Cancer-specific | hypermethylation |
| 20 | 644179 | 644297 | Cancer-specific | hypermethylation |
| 20 | 644287 | 644426 | Cancer-specific | hypermethylation |
| 20 | 9496501 | 9496588 | Cancer-specific | hypermethylation |
| 20 | 983231 | 983258 | Cancer-specific | hypermethylation |
| 20 | 983336 | 983429 | Cancer-specific | hypermethylation |
| 21 | 15588718 | 15588837 | Cancer-specific | hypermethylation |
| 21 | 38076756 | 38076836 | Cancer-specific | hypermethylation |
| 21 | 38079495 | 38079678 | Cancer-specific | hypermethylation |
| 21 | 38079675 | 38079734 | Cancer-specific | hypermethylation |
| 21 | 38378507 | 38378641 | Cancer-specific | hypermethylation |
| 21 | 40984770 | 40984899 | Cancer-specific | hypermethylation |
| 21 | 43786525 | 43786734 | Both | hypomethylation |
| 21 | 43786817 | 43787050 | Cancer-specific | hypomethylation |
| 21 | 45877607 | 45877951 | Both | hypomethylation |
| 21 | 46823951 | 46824069 | Both | hypermethylation |
| 21 | 46824119 | 46824254 | Both | hypermethylation |
| 22 | 19138085 | 19138198 | Cancer-specific | hypermethylation |
| 22 | 19138224 | 19138334 | Cancer-specific | hypermethylation |
| 22 | 19753383 | 19753509 | Cancer-specific | hypermethylation |
| 22 | 19753505 | 19753625 | Cancer-specific | hypermethylation |
| 22 | 20792357 | 20792489 | Cancer-specific | hypermethylation |
| 22 | 21386773 | 21386901 | Cancer-specific | hypermethylation |
| 22 | 22862695 | 22862809 | Cancer-specific | hypermethylation |
| 22 | 22862834 | 22862919 | Cancer-specific | hypermethylation |
| 22 | 25678140 | 25678220 | Cancer-specific | hypermethylation |
| 22 | 27053202 | 27053409 | Cancer-specific | hypermethylation |
| 22 | 28838518 | 28838544 | Cancer-specific | hypermethylation |
| 22 | 28838580 | 28838638 | Cancer-specific | hypermethylation |
| 22 | 28839018 | 28839180 | Cancer-specific | hypermethylation |
| 22 | 28839242 | 28839399 | Cancer-specific | hypermethylation |
| 22 | 28839660 | 28839786 | Cancer-specific | hypermethylation |
| 22 | 29876158 | 29876321 | Cancer-specific | hypermethylation |
| 22 | 37808741 | 37808819 | Tissue-specific | hypomethylation |
| 22 | 37808862 | 37809013 | Both | hypomethylation |
| 22 | 38478366 | 38478500 | Cancer-specific | hypermethylation |
| 22 | 38478500 | 38478581 | Both | hypermethylation |
| 22 | 38484770 | 38484958 | Cancer-specific | hypermethylation |
| 22 | 38484950 | 38485139 | Cancer-specific | hypermethylation |
| 22 | 42095340 | 42095422 | Cancer-specific | hypermethylation |
| 22 | 42095423 | 42095492 | Cancer-specific | hypermethylation |
| 22 | 42679327 | 42679451 | Cancer-specific | hypermethylation |
| 22 | 42679562 | 42679676 | Cancer-specific | hypermethylation |
| 22 | 46262729 | 46262836 | Cancer-specific | hypermethylation |
| 22 | 46262840 | 46262970 | Cancer-specific | hypermethylation |
| 22 | 46402951 | 46403054 | Both | hypermethylation |
| 22 | 46403072 | 46403253 | Both | hypermethylation |
| 22 | 46658782 | 46658896 | Cancer-specific | hypermethylation |
| 22 | 46658886 | 46659029 | Cancer-specific | hypermethylation |
| 2 | 101034199 | 101034330 | Cancer-specific | hypermethylation |
| 2 | 10219537 | 10219712 | Cancer-specific | hypermethylation |
| 2 | 10219701 | 10219785 | Cancer-specific | hypermethylation |
| 2 | 105362883 | 105362990 | Both | hypomethylation |
| 2 | 105363055 | 105363247 | Both | hypomethylation |
| 2 | 105458983 | 105459270 | Cancer-specific | hypermethylation |
| 2 | 105459281 | 105459442 | Cancer-specific | hypermethylation |
| 2 | 105459654 | 105459730 | Cancer-specific | hypermethylation |
| 2 | 105459729 | 105459889 | Cancer-specific | hypermethylation |
| 2 | 105460256 | 105460385 | Cancer-specific | hypermethylation |
| 2 | 105470434 | 105470559 | Cancer-specific | hypermethylation |
| 2 | 105470549 | 105470650 | Cancer-specific | hypermethylation |
| 2 | 105478568 | 105478623 | Cancer-specific | hypermethylation |
| 2 | 105478635 | 105478707 | Cancer-specific | hypermethylation |
| 2 | 105480265 | 105480397 | Cancer-specific | hypermethylation |
| 2 | 105480400 | 105480510 | Cancer-specific | hypermethylation |
| 2 | 106388857 | 106389014 | Both | hypomethylation |
| 2 | 106681864 | 106681952 | Cancer-specific | hypermethylation |
| 2 | 106681976 | 106682106 | Cancer-specific | hypermethylation |
| 2 | 108602779 | 108602889 | Cancer-specific | hypermethylation |
| 2 | 112456804 | 112456903 | Tissue-specific | hypomethylation |
| 2 | 113931391 | 113931572 | Both | hypermethylation |
| 2 | 114034471 | 114034653 | Cancer-specific | hypermethylation |
| 2 | 114034661 | 114034860 | Cancer-specific | hypermethylation |
| 2 | 115920649 | 115920739 | Cancer-specific | hypermethylation |
| 2 | 11810056 | 11810154 | Cancer-specific | hypermethylation |
| 2 | 118982240 | 118982376 | Cancer-specific | hypermethylation |
| 2 | 118982371 | 118982587 | Cancer-specific | hypermethylation |
| 2 | 1204250 | 1204601 | Both | hypomethylation |
| 2 | 121494288 | 121494365 | Cancer-specific | hypermethylation |
| 2 | 121982034 | 121982291 | Cancer-specific | hypomethylation |
| 2 | 127782989 | 127783214 | Cancer-specific | hypermethylation |
| 2 | 12858522 | 12858689 | Cancer-specific | hypermethylation |
| 2 | 131485359 | 131485441 | Cancer-specific | hypermethylation |
| 2 | 135475597 | 135475862 | Cancer-specific | hypermethylation |
| 2 | 144694300 | 144694439 | Cancer-specific | hypermethylation |
| 2 | 144694432 | 144694531 | Cancer-specific | hypermethylation |
| 2 | 144694964 | 144695076 | Cancer-specific | hypermethylation |
| 2 | 14773858 | 14773980 | Cancer-specific | hypermethylation |
| 2 | 148601437 | 148601523 | Cancer-specific | hypermethylation |
| 2 | 154728188 | 154728315 | Cancer-specific | hypermethylation |
| 2 | 157177316 | 157177440 | Cancer-specific | hypermethylation |
| 2 | 157178088 | 157178256 | Cancer-specific | hypermethylation |
| 2 | 157178246 | 157178351 | Cancer-specific | hypermethylation |
| 2 | 162273312 | 162273390 | Cancer-specific | hypermethylation |
| 2 | 162280395 | 162280526 | Cancer-specific | hypermethylation |
| 2 | 162280527 | 162280640 | Cancer-specific | hypermethylation |
| 2 | 162283374 | 162283728 | Cancer-specific | hypermethylation |
| 2 | 162283721 | 162284096 | Cancer-specific | hypermethylation |
| 2 | 169887812 | 169888039 | Both | hypomethylation |
| 2 | 171674736 | 171674818 | Cancer-specific | hypermethylation |
| 2 | 171674817 | 171674975 | Cancer-specific | hypermethylation |
| 2 | 173099476 | 173099598 | Cancer-specific | hypermethylation |
| 2 | 173099590 | 173099721 | Cancer-specific | hypermethylation |
| 2 | 1748009 | 1748728 | Cancer-specific | hypermethylation |
| 2 | 175190920 | 175191073 | Cancer-specific | hypermethylation |
| 2 | 175191062 | 175191151 | Cancer-specific | hypermethylation |
| 2 | 175191651 | 175191871 | Cancer-specific | hypermethylation |
| 2 | 175199549 | 175199677 | Cancer-specific | hypermethylation |
| 2 | 175199684 | 175199750 | Cancer-specific | hypermethylation |
| 2 | 175199756 | 175199928 | Cancer-specific | hypermethylation |
| 2 | 175200835 | 175200955 | Cancer-specific | hypermethylation |
| 2 | 175205739 | 175205792 | Cancer-specific | hypermethylation |
| 2 | 175205814 | 175205901 | Cancer-specific | hypermethylation |
| 2 | 175548013 | 175548061 | Cancer-specific | hypermethylation |
| 2 | 176333698 | 176333901 | Both | hypomethylation |
| 2 | 176947822 | 176947928 | Cancer-specific | hypermethylation |
| 2 | 176947928 | 176948064 | Cancer-specific | hypermethylation |
| 2 | 176969108 | 176969232 | Cancer-specific | hypermethylation |
| 2 | 176969226 | 176969323 | Cancer-specific | hypermethylation |
| 2 | 176987360 | 176987465 | Cancer-specific | hypermethylation |
| 2 | 176987455 | 176987596 | Cancer-specific | hypermethylation |
| 2 | 176994630 | 176994790 | Cancer-specific | hypermethylation |
| 2 | 177001215 | 177001270 | Cancer-specific | hypermethylation |
| 2 | 177001264 | 177001387 | Cancer-specific | hypermethylation |
| 2 | 177017166 | 177017260 | Cancer-specific | hypermethylation |
| 2 | 177017260 | 177017396 | Cancer-specific | hypermethylation |
| 2 | 182322342 | 182322581 | Cancer-specific | hypermethylation |
| 2 | 186603434 | 186603688 | Cancer-specific | hypermethylation |
| 2 | 1879340 | 1879613 | Both | hypomethylation |
| 2 | 190044480 | 190044511 | Cancer-specific | hypermethylation |
| 2 | 1926636 | 1926842 | Both | hypomethylation |
| 2 | 1926881 | 1927149 | Both | hypomethylation |
| 2 | 198650873 | 198651176 | Cancer-specific | hypermethylation |
| 2 | 198651261 | 198651605 | Cancer-specific | hypermethylation |
| 2 | 200326584 | 200326813 | Cancer-specific | hypermethylation |
| 2 | 200327229 | 200327403 | Cancer-specific | hypermethylation |
| 2 | 200327406 | 200327583 | Cancer-specific | hypermethylation |
| 2 | 202897322 | 202897437 | Cancer-specific | hypermethylation |
| 2 | 202899756 | 202899878 | Cancer-specific | hypermethylation |
| 2 | 202899870 | 202899989 | Cancer-specific | hypermethylation |
| 2 | 206546533 | 206546683 | Cancer-specific | hypermethylation |
| 2 | 206546673 | 206546785 | Cancer-specific | hypermethylation |
| 2 | 207138956 | 207139054 | Cancer-specific | hypermethylation |
| 2 | 207139313 | 207139420 | Cancer-specific | hypermethylation |
| 2 | 207139424 | 207139555 | Cancer-specific | hypermethylation |
| 2 | 207507156 | 207507303 | Cancer-specific | hypermethylation |
| 2 | 20865545 | 20865658 | Cancer-specific | hypermethylation |
| 2 | 20865659 | 20865776 | Cancer-specific | hypermethylation |
| 2 | 209271715 | 209271823 | Cancer-specific | hypermethylation |
| 2 | 209271853 | 209271896 | Cancer-specific | hypermethylation |
| 2 | 217497767 | 217497830 | Cancer-specific | hypermethylation |
| 2 | 220361510 | 220361595 | Cancer-specific | hypermethylation |
| 2 | 220361593 | 220361733 | Both | hypermethylation |
| 2 | 220416905 | 220417153 | Cancer-specific | hypermethylation |
| 2 | 221813829 | 221814076 | Both | hypomethylation |
| 2 | 223162685 | 223162882 | Cancer-specific | hypermethylation |
| 2 | 223162874 | 223162987 | Cancer-specific | hypermethylation |
| 2 | 223163219 | 223163333 | Cancer-specific | hypermethylation |
| 2 | 223177165 | 223177276 | Cancer-specific | hypermethylation |
| 2 | 228736231 | 228736279 | Cancer-specific | hypermethylation |
| 2 | 228736267 | 228736378 | Cancer-specific | hypermethylation |
| 2 | 228736378 | 228736576 | Cancer-specific | hypermethylation |
| 2 | 230578098 | 230578248 | Cancer-specific | hypermethylation |
| 2 | 230578837 | 230578897 | Cancer-specific | hypermethylation |
| 2 | 233215975 | 233216096 | Cancer-specific | hypermethylation |
| 2 | 235704735 | 235705065 | Both | hypomethylation |
| 2 | 237069934 | 237070264 | Tissue-specific | hypomethylation |
| 2 | 237072874 | 237073015 | Cancer-specific | hypermethylation |
| 2 | 237073009 | 237073112 | Cancer-specific | hypermethylation |
| 2 | 237077136 | 237077272 | Cancer-specific | hypermethylation |
| 2 | 241497286 | 241497419 | Cancer-specific | hypermethylation |
| 2 | 241497408 | 241497522 | Cancer-specific | hypermethylation |
| 2 | 25391538 | 25391623 | Cancer-specific | hypermethylation |
| 2 | 25391643 | 25391780 | Cancer-specific | hypermethylation |
| 2 | 26395352 | 26395472 | Cancer-specific | hypermethylation |
| 2 | 26395471 | 26395571 | Cancer-specific | hypermethylation |
| 2 | 264041 | 264127 | Cancer-specific | hypermethylation |
| 2 | 264116 | 264185 | Cancer-specific | hypermethylation |
| 2 | 264186 | 264325 | Cancer-specific | hypermethylation |
| 2 | 27070468 | 27070542 | Cancer-specific | hypermethylation |
| 2 | 27530375 | 27530481 | Cancer-specific | hypermethylation |
| 2 | 29033192 | 29033326 | Cancer-specific | hypermethylation |
| 2 | 29033345 | 29033409 | Cancer-specific | hypermethylation |
| 2 | 29033654 | 29033857 | Cancer-specific | hypermethylation |
| 2 | 29033857 | 29034041 | Cancer-specific | hypermethylation |
| 2 | 30143787 | 30143848 | Cancer-specific | hypermethylation |
| 2 | 31360742 | 31360943 | Cancer-specific | hypermethylation |
| 2 | 31361750 | 31361827 | Cancer-specific | hypermethylation |
| 2 | 38302547 | 38302675 | Cancer-specific | hypermethylation |
| 2 | 38755200 | 38755347 | Cancer-specific | hypermethylation |
| 2 | 38755338 | 38755424 | Cancer-specific | hypermethylation |
| 2 | 39187416 | 39187546 | Cancer-specific | hypermethylation |
| 2 | 39187536 | 39187660 | Cancer-specific | hypermethylation |
| 2 | 40679171 | 40679194 | Cancer-specific | hypermethylation |
| 2 | 45028588 | 45028719 | Cancer-specific | hypermethylation |
| 2 | 45028757 | 45028972 | Cancer-specific | hypermethylation |
| 2 | 45029790 | 45029898 | Cancer-specific | hypermethylation |
| 2 | 45029901 | 45030032 | Cancer-specific | hypermethylation |
| 2 | 45155439 | 45155574 | Cancer-specific | hypermethylation |
| 2 | 45156015 | 45156117 | Cancer-specific | hypermethylation |
| 2 | 45159357 | 45159511 | Both | hypermethylation |
| 2 | 45159545 | 45159903 | Cancer-specific | hypermethylation |
| 2 | 45166766 | 45166957 | Cancer-specific | hypermethylation |
| 2 | 45166963 | 45167118 | Cancer-specific | hypermethylation |
| 2 | 45168140 | 45168258 | Cancer-specific | hypermethylation |
| 2 | 45168258 | 45168416 | Cancer-specific | hypermethylation |
| 2 | 45169746 | 45169902 | Cancer-specific | hypermethylation |
| 2 | 45169900 | 45170065 | Cancer-specific | hypermethylation |
| 2 | 45231104 | 45231343 | Cancer-specific | hypermethylation |
| 2 | 45231346 | 45231731 | Cancer-specific | hypermethylation |
| 2 | 45233404 | 45233641 | Cancer-specific | hypermethylation |
| 2 | 45233641 | 45234001 | Both | hypermethylation |
| 2 | 45237565 | 45237668 | Cancer-specific | hypermethylation |
| 2 | 45237671 | 45237798 | Cancer-specific | hypermethylation |
| 2 | 45240441 | 45240556 | Cancer-specific | hypermethylation |
| 2 | 45240556 | 45240685 | Cancer-specific | hypermethylation |
| 2 | 45879563 | 45879629 | Cancer-specific | hypermethylation |
| 2 | 45879629 | 45879774 | Cancer-specific | hypermethylation |
| 2 | 5833245 | 5833361 | Cancer-specific | hypermethylation |
| 2 | 5833365 | 5833490 | Cancer-specific | hypermethylation |
| 2 | 5836249 | 5836373 | Cancer-specific | hypermethylation |
| 2 | 5836362 | 5836491 | Cancer-specific | hypermethylation |
| 2 | 63278936 | 63279060 | Cancer-specific | hypermethylation |
| 2 | 63279054 | 63279241 | Both | hypermethylation |
| 2 | 63280320 | 63280632 | Both | hypermethylation |
| 2 | 63280961 | 63281306 | Cancer-specific | hypermethylation |
| 2 | 63281310 | 63281626 | Cancer-specific | hypermethylation |
| 2 | 63281920 | 63282075 | Both | hypermethylation |
| 2 | 63282124 | 63282302 | Both | hypermethylation |
| 2 | 63285943 | 63286082 | Cancer-specific | hypermethylation |
| 2 | 63286099 | 63286255 | Cancer-specific | hypermethylation |
| 2 | 66667032 | 66667114 | Both | hypermethylation |
| 2 | 66667163 | 66667231 | Both | hypermethylation |
| 2 | 69027032 | 69027096 | Both | hypomethylation |
| 2 | 7058369 | 7058552 | Cancer-specific | hypermethylation |
| 2 | 71115921 | 71116110 | Cancer-specific | hypermethylation |
| 2 | 71116100 | 71116314 | Cancer-specific | hypermethylation |
| 2 | 73146082 | 73146183 | Cancer-specific | hypermethylation |
| 2 | 73147357 | 73147651 | Cancer-specific | hypermethylation |
| 2 | 73147661 | 73147778 | Cancer-specific | hypermethylation |
| 2 | 73147782 | 73147865 | Cancer-specific | hypermethylation |
| 2 | 73429364 | 73429457 | Cancer-specific | hypermethylation |
| 2 | 73495945 | 73496268 | Both | hypermethylation |
| 2 | 73496829 | 73497056 | Both | hypermethylation |
| 2 | 73518589 | 73518723 | Cancer-specific | hypermethylation |
| 2 | 73518715 | 73518815 | Cancer-specific | hypermethylation |
| 2 | 74741027 | 74741154 | Cancer-specific | hypermethylation |
| 2 | 74741149 | 74741259 | Cancer-specific | hypermethylation |
| 2 | 74741816 | 74741929 | Cancer-specific | hypermethylation |
| 2 | 74742185 | 74742281 | Cancer-specific | hypermethylation |
| 2 | 74742295 | 74742385 | Cancer-specific | hypermethylation |
| 2 | 74742659 | 74742776 | Cancer-specific | hypermethylation |
| 2 | 74742777 | 74742898 | Cancer-specific | hypermethylation |
| 2 | 80530133 | 80530347 | Cancer-specific | hypermethylation |
| 2 | 80530335 | 80530551 | Cancer-specific | hypermethylation |
| 2 | 85640833 | 85641025 | Cancer-specific | hypermethylation |
| 2 | 86719495 | 86719509 | Both | hypomethylation |
| 2 | 9403681 | 9403843 | Both | hypermethylation |
| 2 | 95522882 | 95522964 | Cancer-specific | hypermethylation |
| 2 | 95692309 | 95692437 | Cancer-specific | hypermethylation |
| 2 | 95984268 | 95984419 | Both | hypomethylation |
| 2 | 96050680 | 96050733 | Cancer-specific | hypermethylation |
| 2 | 96990793 | 96991061 | Cancer-specific | hypermethylation |
| 2 | 96991051 | 96991144 | Cancer-specific | hypermethylation |
| 2 | 96991138 | 96991272 | Cancer-specific | hypermethylation |
| 2 | 98962793 | 98962909 | Cancer-specific | hypermethylation |
| 2 | 98962907 | 98962981 | Cancer-specific | hypermethylation |
| 2 | 99553303 | 99553487 | Cancer-specific | hypermethylation |
| 2 | 99553487 | 99553666 | Cancer-specific | hypermethylation |
| 2 | 99553662 | 99553905 | Cancer-specific | hypermethylation |
| 3 | 106959857 | 106959931 | Cancer-specific | hypermethylation |
| 3 | 10749876 | 10750024 | Cancer-specific | hypermethylation |
| 3 | 11178467 | 11178805 | Both | hypomethylation |
| 3 | 112358344 | 112358660 | Cancer-specific | hypomethylation |
| 3 | 12045385 | 12045501 | Cancer-specific | hypermethylation |
| 3 | 12045533 | 12045579 | Cancer-specific | hypermethylation |
| 3 | 123753122 | 123753205 | Cancer-specific | hypermethylation |
| 3 | 125075951 | 125076111 | Cancer-specific | hypermethylation |
| 3 | 125076105 | 125076182 | Both | hypermethylation |
| 3 | 125677156 | 125677294 | Cancer-specific | hypermethylation |
| 3 | 125677298 | 125677407 | Cancer-specific | hypermethylation |
| 3 | 125690934 | 125691037 | Cancer-specific | hypermethylation |
| 3 | 127634181 | 127634305 | Cancer-specific | hypermethylation |
| 3 | 127634338 | 127634418 | Cancer-specific | hypermethylation |
| 3 | 128720770 | 128720920 | Cancer-specific | hypermethylation |
| 3 | 129693463 | 129693614 | Cancer-specific | hypermethylation |
| 3 | 132757193 | 132757292 | Cancer-specific | hypermethylation |
| 3 | 134369720 | 134369953 | Cancer-specific | hypermethylation |
| 3 | 136538810 | 136538901 | Cancer-specific | hypermethylation |
| 3 | 136538894 | 136539035 | Cancer-specific | hypermethylation |
| 3 | 137484008 | 137484119 | Cancer-specific | hypermethylation |
| 3 | 137487074 | 137487134 | Cancer-specific | hypermethylation |
| 3 | 137487835 | 137487919 | Cancer-specific | hypermethylation |
| 3 | 138665868 | 138665976 | Cancer-specific | hypermethylation |
| 3 | 138665977 | 138666151 | Cancer-specific | hypermethylation |
| 3 | 138679238 | 138679384 | Cancer-specific | hypermethylation |
| 3 | 138679385 | 138679485 | Cancer-specific | hypermethylation |
| 3 | 139653424 | 139653514 | Cancer-specific | hypermethylation |
| 3 | 139653523 | 139653641 | Cancer-specific | hypermethylation |
| 3 | 142837879 | 142837982 | Cancer-specific | hypermethylation |
| 3 | 142837988 | 142838097 | Cancer-specific | hypermethylation |
| 3 | 142838363 | 142838447 | Cancer-specific | hypermethylation |
| 3 | 142839455 | 142839635 | Cancer-specific | hypermethylation |
| 3 | 142839624 | 142839780 | Cancer-specific | hypermethylation |
| 3 | 142839769 | 142840096 | Cancer-specific | hypermethylation |
| 3 | 147089283 | 147089437 | Cancer-specific | hypermethylation |
| 3 | 147106099 | 147106334 | Cancer-specific | hypermethylation |
| 3 | 147106363 | 147106615 | Cancer-specific | hypermethylation |
| 3 | 147124323 | 147124424 | Cancer-specific | hypermethylation |
| 3 | 147124516 | 147124536 | Cancer-specific | hypermethylation |
| 3 | 147140813 | 147140892 | Cancer-specific | hypermethylation |
| 3 | 147140907 | 147141010 | Cancer-specific | hypermethylation |
| 3 | 14852534 | 14852708 | Cancer-specific | hypermethylation |
| 3 | 14852702 | 14852864 | Cancer-specific | hypermethylation |
| 3 | 150802944 | 150803033 | Cancer-specific | hypermethylation |
| 3 | 150803039 | 150803124 | Cancer-specific | hypermethylation |
| 3 | 152553650 | 152553788 | Cancer-specific | hypermethylation |
| 3 | 157821226 | 157821414 | Cancer-specific | hypermethylation |
| 3 | 157821425 | 157821655 | Cancer-specific | hypermethylation |
| 3 | 157821795 | 157821935 | Cancer-specific | hypermethylation |
| 3 | 157821980 | 157822104 | Cancer-specific | hypermethylation |
| 3 | 157822967 | 157823128 | Cancer-specific | hypermethylation |
| 3 | 157823120 | 157823262 | Cancer-specific | hypermethylation |
| 3 | 157824095 | 157824224 | Cancer-specific | hypermethylation |
| 3 | 159944525 | 159944637 | Cancer-specific | hypermethylation |
| 3 | 160823169 | 160823325 | Cancer-specific | hypermethylation |
| 3 | 169530141 | 169530260 | Cancer-specific | hypermethylation |
| 3 | 169530258 | 169530335 | Cancer-specific | hypermethylation |
| 3 | 169530332 | 169530394 | Cancer-specific | hypermethylation |
| 3 | 170137253 | 170137559 | Cancer-specific | hypermethylation |
| 3 | 170137563 | 170137817 | Cancer-specific | hypermethylation |
| 3 | 170302883 | 170302907 | Cancer-specific | hypermethylation |
| 3 | 172165564 | 172165762 | Cancer-specific | hypermethylation |
| 3 | 172165767 | 172166004 | Cancer-specific | hypermethylation |
| 3 | 172166115 | 172166330 | Cancer-specific | hypermethylation |
| 3 | 172166503 | 172166643 | Cancer-specific | hypermethylation |
| 3 | 172167562 | 172167700 | Cancer-specific | hypermethylation |
| 3 | 172167693 | 172167859 | Cancer-specific | hypermethylation |
| 3 | 173115969 | 173116056 | Cancer-specific | hypermethylation |
| 3 | 179754514 | 179754610 | Cancer-specific | hypermethylation |
| 3 | 179754778 | 179754854 | Cancer-specific | hypermethylation |
| 3 | 179754842 | 179754893 | Cancer-specific | hypermethylation |
| 3 | 179754881 | 179754981 | Cancer-specific | hypermethylation |
| 3 | 179755109 | 179755197 | Cancer-specific | hypermethylation |
| 3 | 180397685 | 180397958 | Cancer-specific | hypermethylation |
| 3 | 181413249 | 181413348 | Cancer-specific | hypermethylation |
| 3 | 181413366 | 181413489 | Cancer-specific | hypermethylation |
| 3 | 181413840 | 181413972 | Cancer-specific | hypermethylation |
| 3 | 181437178 | 181437283 | Cancer-specific | hypermethylation |
| 3 | 181437284 | 181437424 | Cancer-specific | hypermethylation |
| 3 | 182972202 | 182972350 | Cancer-specific | hypermethylation |
| 3 | 182972344 | 182972451 | Cancer-specific | hypermethylation |
| 3 | 184095856 | 184096117 | Both | hypomethylation |
| 3 | 186490220 | 186490341 | Cancer-specific | hypermethylation |
| 3 | 186490337 | 186490460 | Cancer-specific | hypermethylation |
| 3 | 189837957 | 189838121 | Cancer-specific | hypermethylation |
| 3 | 189838113 | 189838196 | Cancer-specific | hypermethylation |
| 3 | 19189447 | 19189582 | Cancer-specific | hypermethylation |
| 3 | 19189634 | 19189815 | Cancer-specific | hypermethylation |
| 3 | 19189804 | 19189913 | Cancer-specific | hypermethylation |
| 3 | 192232082 | 192232203 | Cancer-specific | hypermethylation |
| 3 | 196755858 | 196755973 | Cancer-specific | hypermethylation |
| 3 | 196755966 | 196756067 | Cancer-specific | hypermethylation |
| 3 | 197300107 | 197300263 | Both | hypomethylation |
| 3 | 2141607 | 2141707 | Cancer-specific | hypermethylation |
| 3 | 2141695 | 2141790 | Cancer-specific | hypermethylation |
| 3 | 238822 | 239017 | Cancer-specific | hypermethylation |
| 3 | 32860348 | 32860428 | Cancer-specific | hypermethylation |
| 3 | 32860425 | 32860560 | Cancer-specific | hypermethylation |
| 3 | 33260700 | 33260789 | Cancer-specific | hypermethylation |
| 3 | 3840621 | 3840866 | Cancer-specific | hypermethylation |
| 3 | 39850318 | 39850402 | Cancer-specific | hypermethylation |
| 3 | 39850402 | 39850521 | Cancer-specific | hypermethylation |
| 3 | 44037419 | 44037552 | Cancer-specific | hypermethylation |
| 3 | 44381569 | 44381594 | Both | hypermethylation |
| 3 | 44726943 | 44727082 | Cancer-specific | hypermethylation |
| 3 | 45837346 | 45837488 | Cancer-specific | hypermethylation |
| 3 | 45837487 | 45837579 | Cancer-specific | hypermethylation |
| 3 | 48699248 | 48699358 | Cancer-specific | hypermethylation |
| 3 | 48699366 | 48699488 | Cancer-specific | hypermethylation |
| 3 | 49028055 | 49028143 | Cancer-specific | hypermethylation |
| 3 | 49028139 | 49028197 | Cancer-specific | hypermethylation |
| 3 | 49028188 | 49028259 | Cancer-specific | hypermethylation |
| 3 | 49314275 | 49314445 | Cancer-specific | hypermethylation |
| 3 | 49314438 | 49314496 | Cancer-specific | hypermethylation |
| 3 | 50284091 | 50284154 | Both | hypermethylation |
| 3 | 50284267 | 50284312 | Both | hypermethylation |
| 3 | 50378156 | 50378265 | Cancer-specific | hypermethylation |
| 3 | 50378264 | 50378379 | Cancer-specific | hypermethylation |
| 3 | 5137662 | 5137907 | Cancer-specific | hypermethylation |
| 3 | 5137921 | 5138084 | Cancer-specific | hypermethylation |
| 3 | 54155538 | 54155675 | Cancer-specific | hypermethylation |
| 3 | 54155673 | 54155783 | Cancer-specific | hypermethylation |
| 3 | 56501481 | 56501530 | Cancer-specific | hypermethylation |
| 3 | 56501553 | 56501692 | Cancer-specific | hypermethylation |
| 3 | 61550256 | 61550412 | Cancer-specific | hypermethylation |
| 3 | 61550436 | 61550494 | Cancer-specific | hypermethylation |
| 3 | 62355783 | 62355887 | Cancer-specific | hypermethylation |
| 3 | 62355877 | 62355992 | Cancer-specific | hypermethylation |
| 3 | 68056854 | 68056949 | Cancer-specific | hypermethylation |
| 3 | 68056957 | 68057087 | Cancer-specific | hypermethylation |
| 3 | 68057260 | 68057282 | Cancer-specific | hypermethylation |
| 3 | 68057299 | 68057397 | Cancer-specific | hypermethylation |
| 3 | 68980925 | 68981018 | Cancer-specific | hypermethylation |
| 3 | 68981027 | 68981131 | Cancer-specific | hypermethylation |
| 3 | 71802618 | 71802709 | Cancer-specific | hypermethylation |
| 3 | 72788356 | 72788422 | Both | hypermethylation |
| 3 | 73674110 | 73674173 | Cancer-specific | hypermethylation |
| 4 | 107956836 | 107956955 | Cancer-specific | hypermethylation |
| 4 | 108853185 | 108853260 | Cancer-specific | hypermethylation |
| 4 | 108853253 | 108853327 | Cancer-specific | hypermethylation |
| 4 | 110223481 | 110223715 | Cancer-specific | hypermethylation |
| 4 | 110223706 | 110223907 | Cancer-specific | hypermethylation |
| 4 | 113444175 | 113444255 | Cancer-specific | hypermethylation |
| 4 | 113444274 | 113444441 | Cancer-specific | hypermethylation |
| 4 | 119809838 | 119809978 | Cancer-specific | hypermethylation |
| 4 | 119809968 | 119810046 | Cancer-specific | hypermethylation |
| 4 | 1203535 | 1203874 | Tissue-specific | hypermethylation |
| 4 | 121844084 | 121844235 | Cancer-specific | hypermethylation |
| 4 | 122686340 | 122686463 | Cancer-specific | hypermethylation |
| 4 | 122686481 | 122686571 | Cancer-specific | hypermethylation |
| 4 | 134069202 | 134069332 | Cancer-specific | hypermethylation |
| 4 | 13525500 | 13525705 | Cancer-specific | hypermethylation |
| 4 | 13525698 | 13525851 | Cancer-specific | hypermethylation |
| 4 | 13544185 | 13544308 | Cancer-specific | hypermethylation |
| 4 | 13544357 | 13544554 | Both | hypermethylation |
| 4 | 1396466 | 1396579 | Cancer-specific | hypermethylation |
| 4 | 1396580 | 1396716 | Cancer-specific | hypermethylation |
| 4 | 142053713 | 142053825 | Cancer-specific | hypermethylation |
| 4 | 148402450 | 148402557 | Cancer-specific | hypermethylation |
| 4 | 155662388 | 155662548 | Both | hypermethylation |
| 4 | 155662603 | 155662755 | Cancer-specific | hypermethylation |
| 4 | 156297489 | 156297631 | Cancer-specific | hypermethylation |
| 4 | 156680273 | 156680417 | Cancer-specific | hypermethylation |
| 4 | 156680409 | 156680525 | Cancer-specific | hypermethylation |
| 4 | 15780183 | 15780345 | Cancer-specific | hypermethylation |
| 4 | 15780335 | 15780430 | Cancer-specific | hypermethylation |
| 4 | 157997574 | 157997681 | Cancer-specific | hypermethylation |
| 4 | 16085406 | 16085519 | Cancer-specific | hypermethylation |
| 4 | 164265699 | 164265778 | Cancer-specific | hypermethylation |
| 4 | 169737207 | 169737475 | Both | hypermethylation |
| 4 | 172734166 | 172734244 | Cancer-specific | hypermethylation |
| 4 | 172734854 | 172734948 | Cancer-specific | hypermethylation |
| 4 | 174450333 | 174450415 | Cancer-specific | hypermethylation |
| 4 | 174450436 | 174450532 | Cancer-specific | hypermethylation |
| 4 | 175139074 | 175139196 | Cancer-specific | hypermethylation |
| 4 | 176986828 | 176987027 | Cancer-specific | hypermethylation |
| 4 | 176987023 | 176987110 | Cancer-specific | hypermethylation |
| 4 | 17783287 | 17783447 | Cancer-specific | hypermethylation |
| 4 | 185942139 | 185942176 | Cancer-specific | hypermethylation |
| 4 | 186048599 | 186048704 | Cancer-specific | hypermethylation |
| 4 | 186048707 | 186048820 | Cancer-specific | hypermethylation |
| 4 | 187026587 | 187026699 | Cancer-specific | hypermethylation |
| 4 | 187026688 | 187026783 | Cancer-specific | hypermethylation |
| 4 | 187026775 | 187026828 | Cancer-specific | hypermethylation |
| 4 | 188916599 | 188916839 | Cancer-specific | hypermethylation |
| 4 | 20255075 | 20255194 | Cancer-specific | hypermethylation |
| 4 | 20256280 | 20256418 | Cancer-specific | hypermethylation |
| 4 | 206747 | 206951 | Cancer-specific | hypermethylation |
| 4 | 21950122 | 21950271 | Cancer-specific | hypermethylation |
| 4 | 30723166 | 30723282 | Cancer-specific | hypermethylation |
| 4 | 30723283 | 30723410 | Cancer-specific | hypermethylation |
| 4 | 30820941 | 30821172 | Cancer-specific | hypomethylation |
| 4 | 37246110 | 37246237 | Cancer-specific | hypermethylation |
| 4 | 38673046 | 38673151 | Both | hypomethylation |
| 4 | 41875186 | 41875453 | Both | hypermethylation |
| 4 | 41882107 | 41882349 | Cancer-specific | hypermethylation |
| 4 | 41882444 | 41882672 | Cancer-specific | hypermethylation |
| 4 | 41883100 | 41883156 | Cancer-specific | hypermethylation |
| 4 | 42154026 | 42154147 | Cancer-specific | hypermethylation |
| 4 | 42154155 | 42154252 | Cancer-specific | hypermethylation |
| 4 | 42399671 | 42399805 | Cancer-specific | hypermethylation |
| 4 | 42399816 | 42399959 | Cancer-specific | hypermethylation |
| 4 | 4389593 | 4389629 | Cancer-specific | hypermethylation |
| 4 | 46995525 | 46995834 | Cancer-specific | hypermethylation |
| 4 | 52917296 | 52917534 | Cancer-specific | hypermethylation |
| 4 | 54975777 | 54975913 | Cancer-specific | hypermethylation |
| 4 | 54975911 | 54976016 | Cancer-specific | hypermethylation |
| 4 | 570246 | 570339 | Cancer-specific | hypermethylation |
| 4 | 57371594 | 57371699 | Cancer-specific | hypermethylation |
| 4 | 57371712 | 57371834 | Cancer-specific | hypermethylation |
| 4 | 57522138 | 57522217 | Cancer-specific | hypermethylation |
| 4 | 57976802 | 57976874 | Cancer-specific | hypermethylation |
| 4 | 6665780 | 6665902 | Cancer-specific | hypermethylation |
| 4 | 6665898 | 6666029 | Cancer-specific | hypermethylation |
| 4 | 7219751 | 7219877 | Both | hypomethylation |
| 4 | 7219922 | 7219969 | Both | hypomethylation |
| 4 | 76555444 | 76555641 | Cancer-specific | hypermethylation |
| 4 | 76555658 | 76555774 | Cancer-specific | hypermethylation |
| 4 | 76555765 | 76555971 | Cancer-specific | hypermethylation |
| 4 | 7657335 | 7657488 | Both | hypomethylation |
| 4 | 7657504 | 7657656 | Both | hypomethylation |
| 4 | 7940479 | 7940635 | Cancer-specific | hypermethylation |
| 4 | 7940626 | 7940688 | Cancer-specific | hypermethylation |
| 4 | 81031109 | 81031246 | Cancer-specific | hypomethylation |
| 4 | 81106558 | 81106641 | Cancer-specific | hypermethylation |
| 4 | 81106639 | 81106779 | Cancer-specific | hypermethylation |
| 4 | 81189699 | 81189836 | Cancer-specific | hypermethylation |
| 4 | 84035830 | 84035960 | Both | hypomethylation |
| 4 | 91045096 | 91045216 | Both | hypomethylation |
| 4 | 96470279 | 96470411 | Cancer-specific | hypermethylation |
| 4 | 96549935 | 96549949 | Tissue-specific | hypomethylation |
| 5 | 102131988 | 102132028 | Both | hypomethylation |
| 5 | 112073297 | 112073452 | Cancer-specific | hypermethylation |
| 5 | 112073463 | 112073614 | Cancer-specific | hypermethylation |
| 5 | 127873417 | 127873538 | Cancer-specific | hypermethylation |
| 5 | 127873536 | 127873824 | Cancer-specific | hypermethylation |
| 5 | 127874337 | 127874401 | Cancer-specific | hypermethylation |
| 5 | 127874400 | 127874581 | Cancer-specific | hypermethylation |
| 5 | 134880229 | 134880413 | Cancer-specific | hypermethylation |
| 5 | 134880404 | 134880479 | Cancer-specific | hypermethylation |
| 5 | 140474017 | 140474069 | Cancer-specific | hypomethylation |
| 5 | 140753484 | 140753543 | Cancer-specific | hypermethylation |
| 5 | 140856218 | 140856337 | Cancer-specific | hypermethylation |
| 5 | 140856330 | 140856446 | Cancer-specific | hypermethylation |
| 5 | 141891627 | 141892020 | Both | hypomethylation |
| 5 | 1444736 | 1444838 | Cancer-specific | hypermethylation |
| 5 | 1445250 | 1445363 | Cancer-specific | hypermethylation |
| 5 | 1445465 | 1445684 | Cancer-specific | hypermethylation |
| 5 | 145713729 | 145713870 | Cancer-specific | hypermethylation |
| 5 | 157098420 | 157098522 | Cancer-specific | hypermethylation |
| 5 | 157098521 | 157098652 | Cancer-specific | hypermethylation |
| 5 | 158527695 | 158527846 | Cancer-specific | hypermethylation |
| 5 | 158527853 | 158528131 | Cancer-specific | hypermethylation |
| 5 | 1594669 | 1594815 | Cancer-specific | hypermethylation |
| 5 | 159738693 | 159738820 | Cancer-specific | hypermethylation |
| 5 | 160975846 | 160975911 | Cancer-specific | hypermethylation |
| 5 | 16179746 | 16180426 | Cancer-specific | hypermethylation |
| 5 | 166406005 | 166406084 | Cancer-specific | hypermethylation |
| 5 | 16664415 | 16664655 | Cancer-specific | hypomethylation |
| 5 | 16670749 | 16671157 | Cancer-specific | hypomethylation |
| 5 | 16711067 | 16711412 | Both | hypomethylation |
| 5 | 16741968 | 16742361 | Both | hypomethylation |
| 5 | 16812385 | 16812574 | Both | hypomethylation |
| 5 | 16820014 | 16820226 | Both | hypomethylation |
| 5 | 168727774 | 168727888 | Cancer-specific | hypermethylation |
| 5 | 170735906 | 170735990 | Cancer-specific | hypermethylation |
| 5 | 170735997 | 170736139 | Cancer-specific | hypermethylation |
| 5 | 170736209 | 170736475 | Cancer-specific | hypermethylation |
| 5 | 170736468 | 170736696 | Cancer-specific | hypermethylation |
| 5 | 170738154 | 170738304 | Cancer-specific | hypermethylation |
| 5 | 170739220 | 170739302 | Cancer-specific | hypermethylation |
| 5 | 170740546 | 170740965 | Cancer-specific | hypermethylation |
| 5 | 170742483 | 170742615 | Cancer-specific | hypermethylation |
| 5 | 170742625 | 170742732 | Cancer-specific | hypermethylation |
| 5 | 170743443 | 170743592 | Cancer-specific | hypermethylation |
| 5 | 170743592 | 170743682 | Cancer-specific | hypermethylation |
| 5 | 17147764 | 17147935 | Both | hypomethylation |
| 5 | 172175478 | 172175766 | Tissue-specific | hypermethylation |
| 5 | 172175758 | 172175922 | Tissue-specific | hypermethylation |
| 5 | 172656048 | 172656146 | Cancer-specific | hypermethylation |
| 5 | 174178770 | 174178850 | Cancer-specific | hypermethylation |
| 5 | 174178846 | 174178989 | Cancer-specific | hypermethylation |
| 5 | 174872068 | 174872147 | Cancer-specific | hypermethylation |
| 5 | 175298509 | 175298594 | Cancer-specific | hypermethylation |
| 5 | 175792926 | 175793067 | Cancer-specific | hypermethylation |
| 5 | 175793058 | 175793173 | Cancer-specific | hypermethylation |
| 5 | 176057052 | 176057183 | Cancer-specific | hypermethylation |
| 5 | 178017803 | 178017860 | Cancer-specific | hypermethylation |
| 5 | 180479579 | 180479637 | Cancer-specific | hypermethylation |
| 5 | 1882878 | 1883319 | Cancer-specific | hypermethylation |
| 5 | 1883817 | 1883957 | Cancer-specific | hypermethylation |
| 5 | 1883947 | 1884066 | Cancer-specific | hypermethylation |
| 5 | 2751697 | 2751989 | Cancer-specific | hypermethylation |
| 5 | 320782 | 320895 | Cancer-specific | hypermethylation |
| 5 | 320898 | 320985 | Cancer-specific | hypermethylation |
| 5 | 3602148 | 3602424 | Cancer-specific | hypermethylation |
| 5 | 3602441 | 3602652 | Cancer-specific | hypermethylation |
| 5 | 36690201 | 36690303 | Cancer-specific | hypermethylation |
| 5 | 40681205 | 40681338 | Cancer-specific | hypermethylation |
| 5 | 40681346 | 40681447 | Cancer-specific | hypermethylation |
| 5 | 40681679 | 40681871 | Cancer-specific | hypermethylation |
| 5 | 40681860 | 40682152 | Both | hypermethylation |
| 5 | 41870868 | 41870985 | Cancer-specific | hypermethylation |
| 5 | 42952000 | 42952120 | Cancer-specific | hypermethylation |
| 5 | 42952136 | 42952216 | Cancer-specific | hypermethylation |
| 5 | 42992648 | 42992768 | Cancer-specific | hypermethylation |
| 5 | 42992764 | 42992863 | Cancer-specific | hypermethylation |
| 5 | 42992852 | 42993067 | Cancer-specific | hypermethylation |
| 5 | 42994662 | 42994796 | Cancer-specific | hypermethylation |
| 5 | 45696345 | 45696432 | Cancer-specific | hypermethylation |
| 5 | 50259990 | 50260060 | Cancer-specific | hypermethylation |
| 5 | 50260065 | 50260165 | Cancer-specific | hypermethylation |
| 5 | 50685389 | 50685500 | Cancer-specific | hypermethylation |
| 5 | 510132 | 510342 | Both | hypomethylation |
| 5 | 510370 | 510499 | Both | hypomethylation |
| 5 | 528502 | 528622 | Cancer-specific | hypermethylation |
| 5 | 528614 | 528747 | Cancer-specific | hypermethylation |
| 5 | 54179412 | 54179522 | Cancer-specific | hypermethylation |
| 5 | 54179530 | 54179652 | Cancer-specific | hypermethylation |
| 5 | 54519010 | 54519305 | Cancer-specific | hypermethylation |
| 5 | 54519300 | 54519583 | Cancer-specific | hypermethylation |
| 5 | 72529809 | 72529897 | Cancer-specific | hypermethylation |
| 5 | 72594339 | 72594451 | Cancer-specific | hypermethylation |
| 5 | 72678066 | 72678152 | Cancer-specific | hypermethylation |
| 5 | 72678167 | 72678312 | Cancer-specific | hypermethylation |
| 5 | 72715606 | 72715708 | Cancer-specific | hypermethylation |
| 5 | 72732202 | 72732285 | Cancer-specific | hypermethylation |
| 5 | 72732359 | 72732442 | Cancer-specific | hypermethylation |
| 5 | 73623201 | 73623215 | Both | hypomethylation |
| 5 | 7396852 | 7396954 | Cancer-specific | hypermethylation |
| 5 | 75377996 | 75378089 | Cancer-specific | hypermethylation |
| 5 | 75380718 | 75380802 | Cancer-specific | hypermethylation |
| 5 | 76012479 | 76012598 | Cancer-specific | hypermethylation |
| 5 | 76012589 | 76012685 | Cancer-specific | hypermethylation |
| 5 | 76923850 | 76923963 | Cancer-specific | hypermethylation |
| 5 | 76923956 | 76924067 | Cancer-specific | hypermethylation |
| 5 | 76928687 | 76928817 | Cancer-specific | hypermethylation |
| 5 | 77268617 | 77268741 | Cancer-specific | hypermethylation |
| 5 | 77268734 | 77268962 | Cancer-specific | hypermethylation |
| 5 | 79330857 | 79331048 | Cancer-specific | hypermethylation |
| 5 | 79331040 | 79331123 | Cancer-specific | hypermethylation |
| 5 | 79331120 | 79331260 | Cancer-specific | hypermethylation |
| 5 | 87976617 | 87976685 | Cancer-specific | hypermethylation |
| 5 | 94955782 | 94955887 | Cancer-specific | hypermethylation |
| 5 | 94955886 | 94955977 | Cancer-specific | hypermethylation |
| 6 | 100037462 | 100037550 | Cancer-specific | hypermethylation |
| 6 | 100050774 | 100050894 | Cancer-specific | hypermethylation |
| 6 | 100051012 | 100051138 | Cancer-specific | hypermethylation |
| 6 | 100051140 | 100051238 | Cancer-specific | hypermethylation |
| 6 | 100060838 | 100061069 | Cancer-specific | hypermethylation |
| 6 | 100061068 | 100061237 | Cancer-specific | hypermethylation |
| 6 | 100061273 | 100061432 | Cancer-specific | hypermethylation |
| 6 | 100909008 | 100909235 | Both | hypermethylation |
| 6 | 100909231 | 100909361 | Cancer-specific | hypermethylation |
| 6 | 100912815 | 100912913 | Cancer-specific | hypermethylation |
| 6 | 100912928 | 100913009 | Cancer-specific | hypermethylation |
| 6 | 10415471 | 10415706 | Cancer-specific | hypermethylation |
| 6 | 10415758 | 10415969 | Cancer-specific | hypermethylation |
| 6 | 108486008 | 108486130 | Cancer-specific | hypermethylation |
| 6 | 108488215 | 108488330 | Cancer-specific | hypermethylation |
| 6 | 108488328 | 108488437 | Cancer-specific | hypermethylation |
| 6 | 108488677 | 108488851 | Cancer-specific | hypermethylation |
| 6 | 108488855 | 108489033 | Cancer-specific | hypermethylation |
| 6 | 108490804 | 108490924 | Cancer-specific | hypermethylation |
| 6 | 108490969 | 108491021 | Cancer-specific | hypermethylation |
| 6 | 108492260 | 108492416 | Cancer-specific | hypermethylation |
| 6 | 108492430 | 108492613 | Both | hypermethylation |
| 6 | 109267200 | 109267479 | Both | hypermethylation |
| 6 | 110678989 | 110679150 | Cancer-specific | hypermethylation |
| 6 | 110679447 | 110679580 | Cancer-specific | hypermethylation |
| 6 | 110679573 | 110679692 | Cancer-specific | hypermethylation |
| 6 | 117086739 | 117086840 | Cancer-specific | hypermethylation |
| 6 | 117086843 | 117086948 | Cancer-specific | hypermethylation |
| 6 | 118228387 | 118228638 | Cancer-specific | hypermethylation |
| 6 | 118229610 | 118229774 | Cancer-specific | hypermethylation |
| 6 | 118229781 | 118229843 | Cancer-specific | hypermethylation |
| 6 | 127836768 | 127836854 | Cancer-specific | hypermethylation |
| 6 | 133561749 | 133562131 | Cancer-specific | hypermethylation |
| 6 | 133562119 | 133562282 | Cancer-specific | hypermethylation |
| 6 | 133562431 | 133562601 | Cancer-specific | hypermethylation |
| 6 | 137241937 | 137242019 | Cancer-specific | hypermethylation |
| 6 | 137242055 | 137242156 | Cancer-specific | hypermethylation |
| 6 | 137311194 | 137311250 | Cancer-specific | hypermethylation |
| 6 | 137311251 | 137311373 | Cancer-specific | hypermethylation |
| 6 | 1391146 | 1391244 | Cancer-specific | hypermethylation |
| 6 | 1391242 | 1391372 | Cancer-specific | hypermethylation |
| 6 | 13925385 | 13925440 | Cancer-specific | hypermethylation |
| 6 | 149068857 | 149068971 | Cancer-specific | hypermethylation |
| 6 | 149068961 | 149069097 | Cancer-specific | hypermethylation |
| 6 | 149069333 | 149069417 | Cancer-specific | hypermethylation |
| 6 | 150286385 | 150286468 | Cancer-specific | hypermethylation |
| 6 | 150286485 | 150286632 | Cancer-specific | hypermethylation |
| 6 | 152957250 | 152957320 | Cancer-specific | hypermethylation |
| 6 | 1601618 | 1601761 | Cancer-specific | hypermethylation |
| 6 | 1601795 | 1601866 | Cancer-specific | hypermethylation |
| 6 | 1601875 | 1602074 | Cancer-specific | hypermethylation |
| 6 | 166422664 | 166422748 | Cancer-specific | hypermethylation |
| 6 | 166667439 | 166667497 | Cancer-specific | hypermethylation |
| 6 | 167104820 | 167105012 | Cancer-specific | hypomethylation |
| 6 | 167105024 | 167105172 | Both | hypomethylation |
| 6 | 16729526 | 16729704 | Cancer-specific | hypomethylation |
| 6 | 19144012 | 19144404 | Cancer-specific | hypomethylation |
| 6 | 19836913 | 19837022 | Cancer-specific | hypermethylation |
| 6 | 19837025 | 19837133 | Cancer-specific | hypermethylation |
| 6 | 26183816 | 26183836 | Cancer-specific | hypermethylation |
| 6 | 26183892 | 26183917 | Cancer-specific | hypermethylation |
| 6 | 26233908 | 26234048 | Cancer-specific | hypermethylation |
| 6 | 26235070 | 26235329 | Cancer-specific | hypermethylation |
| 6 | 26250563 | 26250699 | Cancer-specific | hypermethylation |
| 6 | 26250705 | 26250943 | Cancer-specific | hypermethylation |
| 6 | 26251695 | 26251912 | Cancer-specific | hypermethylation |
| 6 | 26251903 | 26252008 | Cancer-specific | hypermethylation |
| 6 | 26271598 | 26271834 | Cancer-specific | hypermethylation |
| 6 | 26272214 | 26272546 | Cancer-specific | hypermethylation |
| 6 | 26272535 | 26272629 | Cancer-specific | hypermethylation |
| 6 | 26550684 | 26550910 | Cancer-specific | hypermethylation |
| 6 | 27064974 | 27065094 | Cancer-specific | hypermethylation |
| 6 | 27235781 | 27235860 | Cancer-specific | hypermethylation |
| 6 | 27235862 | 27235941 | Cancer-specific | hypermethylation |
| 6 | 27258672 | 27258789 | Cancer-specific | hypermethylation |
| 6 | 27279887 | 27280088 | Cancer-specific | hypermethylation |
| 6 | 27463105 | 27463257 | Cancer-specific | hypermethylation |
| 6 | 27463252 | 27463371 | Cancer-specific | hypermethylation |
| 6 | 27834638 | 27834721 | Both | hypomethylation |
| 6 | 27835259 | 27835385 | Cancer-specific | hypermethylation |
| 6 | 27835378 | 27835467 | Cancer-specific | hypermethylation |
| 6 | 27840836 | 27841139 | Cancer-specific | hypermethylation |
| 6 | 27841145 | 27841430 | Cancer-specific | hypermethylation |
| 6 | 27858511 | 27858570 | Cancer-specific | hypermethylation |
| 6 | 27858630 | 27858758 | Cancer-specific | hypermethylation |
| 6 | 28175327 | 28175453 | Cancer-specific | hypermethylation |
| 6 | 28175501 | 28175572 | Cancer-specific | hypermethylation |
| 6 | 28175783 | 28175859 | Cancer-specific | hypermethylation |
| 6 | 28249605 | 28249668 | Cancer-specific | hypermethylation |
| 6 | 28249692 | 28249789 | Cancer-specific | hypermethylation |
| 6 | 28410985 | 28411247 | Cancer-specific | hypermethylation |
| 6 | 28411264 | 28411468 | Cancer-specific | hypermethylation |
| 6 | 28782040 | 28782209 | Cancer-specific | hypermethylation |
| 6 | 28979203 | 28979314 | Cancer-specific | hypermethylation |
| 6 | 28979319 | 28979415 | Cancer-specific | hypermethylation |
| 6 | 29407720 | 29408076 | Both | hypomethylation |
| 6 | 29795896 | 29795978 | Cancer-specific | hypermethylation |
| 6 | 29796008 | 29796131 | Cancer-specific | hypermethylation |
| 6 | 30095319 | 30095556 | Cancer-specific | hypermethylation |
| 6 | 30095563 | 30095827 | Cancer-specific | hypermethylation |
| 6 | 30131577 | 30131978 | Both | hypomethylation |
| 6 | 30168302 | 30168387 | Both | hypomethylation |
| 6 | 30418956 | 30419104 | Cancer-specific | hypermethylation |
| 6 | 32087073 | 32087341 | Both | hypomethylation |
| 6 | 32166715 | 32166879 | Cancer-specific | hypomethylation |
| 6 | 32222983 | 32223074 | Cancer-specific | hypermethylation |
| 6 | 32223069 | 32223215 | Cancer-specific | hypermethylation |
| 6 | 32223219 | 32223348 | Cancer-specific | hypermethylation |
| 6 | 33161169 | 33161401 | Cancer-specific | hypermethylation |
| 6 | 33173300 | 33173432 | Cancer-specific | hypomethylation |
| 6 | 33173475 | 33173622 | Cancer-specific | hypomethylation |
| 6 | 33739286 | 33739432 | Cancer-specific | hypermethylation |
| 6 | 33739421 | 33739493 | Cancer-specific | hypermethylation |
| 6 | 36807084 | 36807304 | Cancer-specific | hypermethylation |
| 6 | 36808371 | 36808434 | Cancer-specific | hypermethylation |
| 6 | 36808487 | 36808620 | Cancer-specific | hypermethylation |
| 6 | 37673487 | 37673637 | Cancer-specific | hypermethylation |
| 6 | 37673628 | 37673719 | Cancer-specific | hypermethylation |
| 6 | 38683070 | 38683179 | Cancer-specific | hypermethylation |
| 6 | 38683179 | 38683307 | Cancer-specific | hypermethylation |
| 6 | 391107 | 391311 | Cancer-specific | hypermethylation |
| 6 | 391657 | 392056 | Cancer-specific | hypermethylation |
| 6 | 39281097 | 39281190 | Cancer-specific | hypermethylation |
| 6 | 393117 | 393206 | Cancer-specific | hypermethylation |
| 6 | 393202 | 393363 | Cancer-specific | hypermethylation |
| 6 | 393498 | 393623 | Cancer-specific | hypermethylation |
| 6 | 393613 | 393742 | Cancer-specific | hypermethylation |
| 6 | 40567509 | 40567586 | Cancer-specific | hypermethylation |
| 6 | 41339338 | 41339438 | Cancer-specific | hypermethylation |
| 6 | 41339431 | 41339554 | Cancer-specific | hypermethylation |
| 6 | 43211108 | 43211242 | Cancer-specific | hypermethylation |
| 6 | 43211236 | 43211327 | Cancer-specific | hypermethylation |
| 6 | 43243664 | 43243968 | Cancer-specific | hypermethylation |
| 6 | 43265839 | 43265853 | Both | hypomethylation |
| 6 | 43613285 | 43613340 | Cancer-specific | hypermethylation |
| 6 | 43613333 | 43613470 | Cancer-specific | hypermethylation |
| 6 | 44698690 | 44698866 | Cancer-specific | hypomethylation |
| 6 | 45391123 | 45391199 | Cancer-specific | hypermethylation |
| 6 | 45391195 | 45391265 | Cancer-specific | hypermethylation |
| 6 | 4665173 | 4665460 | Cancer-specific | hypomethylation |
| 6 | 47666241 | 47666537 | Both | hypomethylation |
| 6 | 52227896 | 52227954 | Cancer-specific | hypermethylation |
| 6 | 52227955 | 52228030 | Cancer-specific | hypermethylation |
| 6 | 56819357 | 56819461 | Cancer-specific | hypermethylation |
| 6 | 5851604 | 5851700 | Cancer-specific | hypermethylation |
| 6 | 5996137 | 5996262 | Cancer-specific | hypermethylation |
| 6 | 5996259 | 5996347 | Cancer-specific | hypermethylation |
| 6 | 6002640 | 6002787 | Cancer-specific | hypermethylation |
| 6 | 6002792 | 6002872 | Cancer-specific | hypermethylation |
| 6 | 6004087 | 6004259 | Cancer-specific | hypermethylation |
| 6 | 6004261 | 6004452 | Cancer-specific | hypermethylation |
| 6 | 6007602 | 6007803 | Cancer-specific | hypermethylation |
| 6 | 62996509 | 62996595 | Cancer-specific | hypermethylation |
| 6 | 73935073 | 73935314 | Both | hypomethylation |
| 6 | 7728802 | 7728904 | Cancer-specific | hypermethylation |
| 6 | 78172339 | 78172452 | Cancer-specific | hypermethylation |
| 6 | 79620244 | 79620393 | Cancer-specific | hypermethylation |
| 6 | 79620388 | 79620481 | Cancer-specific | hypermethylation |
| 6 | 84418761 | 84418923 | Cancer-specific | hypermethylation |
| 6 | 84419227 | 84419435 | Cancer-specific | hypermethylation |
| 6 | 85474399 | 85474537 | Cancer-specific | hypermethylation |
| 6 | 88876957 | 88877045 | Cancer-specific | hypermethylation |
| 6 | 88877054 | 88877177 | Cancer-specific | hypermethylation |
| 6 | 94128254 | 94128306 | Cancer-specific | hypermethylation |
| 6 | 99295942 | 99296075 | Cancer-specific | hypermethylation |
| 6 | 99296069 | 99296189 | Cancer-specific | hypermethylation |
| 7 | 105221904 | 105222005 | Cancer-specific | hypermethylation |
| 7 | 105222012 | 105222128 | Cancer-specific | hypermethylation |
| 7 | 112726528 | 112726663 | Cancer-specific | hypermethylation |
| 7 | 117119501 | 117119733 | Cancer-specific | hypermethylation |
| 7 | 117119740 | 117120002 | Cancer-specific | hypermethylation |
| 7 | 121941890 | 121942017 | Cancer-specific | hypermethylation |
| 7 | 121942013 | 121942136 | Cancer-specific | hypermethylation |
| 7 | 121946487 | 121946587 | Cancer-specific | hypermethylation |
| 7 | 121946583 | 121946687 | Cancer-specific | hypermethylation |
| 7 | 121956537 | 121956813 | Cancer-specific | hypermethylation |
| 7 | 121956839 | 121957015 | Cancer-specific | hypermethylation |
| 7 | 126891304 | 126891443 | Cancer-specific | hypermethylation |
| 7 | 1270476 | 1270570 | Cancer-specific | hypermethylation |
| 7 | 1270587 | 1270725 | Cancer-specific | hypermethylation |
| 7 | 127256669 | 127256927 | Both | hypomethylation |
| 7 | 127260267 | 127260366 | Cancer-specific | hypermethylation |
| 7 | 128337614 | 128337711 | Cancer-specific | hypermethylation |
| 7 | 128337718 | 128337795 | Cancer-specific | hypermethylation |
| 7 | 128470473 | 128470596 | Cancer-specific | hypermethylation |
| 7 | 128470587 | 128470677 | Cancer-specific | hypermethylation |
| 7 | 129423330 | 129423475 | Cancer-specific | hypermethylation |
| 7 | 132171187 | 132171276 | Both | hypomethylation |
| 7 | 132171323 | 132171399 | Both | hypomethylation |
| 7 | 133811691 | 133811710 | Both | hypermethylation |
| 7 | 134143125 | 134143251 | Cancer-specific | hypermethylation |
| 7 | 134143242 | 134143371 | Cancer-specific | hypermethylation |
| 7 | 134143708 | 134143928 | Cancer-specific | hypermethylation |
| 7 | 134143917 | 134144125 | Cancer-specific | hypermethylation |
| 7 | 139168358 | 139168472 | Cancer-specific | hypermethylation |
| 7 | 139208012 | 139208071 | Cancer-specific | hypermethylation |
| 7 | 139208729 | 139208836 | Cancer-specific | hypermethylation |
| 7 | 139208845 | 139208977 | Cancer-specific | hypermethylation |
| 7 | 141211989 | 141212082 | Cancer-specific | hypermethylation |
| 7 | 143042502 | 143042605 | Both | hypermethylation |
| 7 | 143042628 | 143042751 | Cancer-specific | hypermethylation |
| 7 | 145813370 | 145813446 | Cancer-specific | hypermethylation |
| 7 | 149412165 | 149412258 | Cancer-specific | hypermethylation |
| 7 | 149412283 | 149412402 | Cancer-specific | hypermethylation |
| 7 | 151107153 | 151107256 | Cancer-specific | hypermethylation |
| 7 | 151107250 | 151107375 | Cancer-specific | hypermethylation |
| 7 | 151107520 | 151107669 | Cancer-specific | hypermethylation |
| 7 | 151107680 | 151107761 | Cancer-specific | hypermethylation |
| 7 | 152622255 | 152622462 | Cancer-specific | hypermethylation |
| 7 | 152622470 | 152622571 | Cancer-specific | hypermethylation |
| 7 | 152622661 | 152622803 | Cancer-specific | hypermethylation |
| 7 | 155164874 | 155164980 | Cancer-specific | hypermethylation |
| 7 | 155164982 | 155165118 | Cancer-specific | hypermethylation |
| 7 | 155166226 | 155166386 | Cancer-specific | hypermethylation |
| 7 | 155249312 | 155249367 | Cancer-specific | hypermethylation |
| 7 | 155252593 | 155252708 | Cancer-specific | hypermethylation |
| 7 | 155258846 | 155258953 | Cancer-specific | hypermethylation |
| 7 | 155302795 | 155302940 | Cancer-specific | hypermethylation |
| 7 | 155302952 | 155303006 | Cancer-specific | hypermethylation |
| 7 | 155596014 | 155596165 | Both | hypermethylation |
| 7 | 155596169 | 155596305 | Both | hypermethylation |
| 7 | 157129210 | 157129278 | Cancer-specific | hypermethylation |
| 7 | 157407980 | 157408057 | Both | hypomethylation |
| 7 | 157408111 | 157408269 | Both | hypomethylation |
| 7 | 157483210 | 157483431 | Cancer-specific | hypermethylation |
| 7 | 157483421 | 157483531 | Cancer-specific | hypermethylation |
| 7 | 157483521 | 157483601 | Cancer-specific | hypermethylation |
| 7 | 157781695 | 157781825 | Cancer-specific | hypomethylation |
| 7 | 158936662 | 158936873 | Cancer-specific | hypermethylation |
| 7 | 158937003 | 158937131 | Cancer-specific | hypermethylation |
| 7 | 158937131 | 158937233 | Cancer-specific | hypermethylation |
| 7 | 158937948 | 158938041 | Cancer-specific | hypermethylation |
| 7 | 158938040 | 158938157 | Cancer-specific | hypermethylation |
| 7 | 19156518 | 19156628 | Cancer-specific | hypermethylation |
| 7 | 19156628 | 19156705 | Cancer-specific | hypermethylation |
| 7 | 19156819 | 19156912 | Cancer-specific | hypermethylation |
| 7 | 19156902 | 19157017 | Cancer-specific | hypermethylation |
| 7 | 19157138 | 19157270 | Cancer-specific | hypermethylation |
| 7 | 20122461 | 20122612 | Both | hypomethylation |
| 7 | 20158197 | 20158296 | Both | hypomethylation |
| 7 | 20167738 | 20168005 | Both | hypomethylation |
| 7 | 20309817 | 20309831 | Both | hypomethylation |
| 7 | 20816099 | 20816282 | Cancer-specific | hypermethylation |
| 7 | 20816300 | 20816440 | Cancer-specific | hypermethylation |
| 7 | 20823873 | 20823990 | Cancer-specific | hypermethylation |
| 7 | 20823979 | 20824118 | Cancer-specific | hypermethylation |
| 7 | 20824126 | 20824246 | Cancer-specific | hypermethylation |
| 7 | 21209235 | 21209345 | Cancer-specific | hypermethylation |
| 7 | 21209365 | 21209437 | Cancer-specific | hypermethylation |
| 7 | 26192860 | 26193068 | Cancer-specific | hypermethylation |
| 7 | 26193069 | 26193230 | Both | hypermethylation |
| 7 | 27135677 | 27135845 | Cancer-specific | hypermethylation |
| 7 | 27135844 | 27135939 | Cancer-specific | hypermethylation |
| 7 | 27136069 | 27136135 | Cancer-specific | hypermethylation |
| 7 | 27136131 | 27136309 | Cancer-specific | hypermethylation |
| 7 | 27179056 | 27179212 | Both | hypermethylation |
| 7 | 27179203 | 27179408 | Both | hypermethylation |
| 7 | 27190982 | 27191035 | Cancer-specific | hypermethylation |
| 7 | 27191050 | 27191212 | Cancer-specific | hypermethylation |
| 7 | 27195834 | 27195952 | Cancer-specific | hypermethylation |
| 7 | 27195947 | 27196057 | Cancer-specific | hypermethylation |
| 7 | 27196398 | 27196485 | Cancer-specific | hypermethylation |
| 7 | 27204854 | 27205592 | Cancer-specific | hypermethylation |
| 7 | 27208065 | 27208238 | Cancer-specific | hypermethylation |
| 7 | 27208278 | 27208483 | Both | hypermethylation |
| 7 | 27209680 | 27209797 | Cancer-specific | hypermethylation |
| 7 | 27209797 | 27209905 | Cancer-specific | hypermethylation |
| 7 | 27213484 | 27213608 | Cancer-specific | hypermethylation |
| 7 | 27213598 | 27213735 | Cancer-specific | hypermethylation |
| 7 | 27213825 | 27214003 | Cancer-specific | hypermethylation |
| 7 | 27214003 | 27214213 | Cancer-specific | hypermethylation |
| 7 | 27232710 | 27232874 | Cancer-specific | hypermethylation |
| 7 | 27232901 | 27233378 | Cancer-specific | hypermethylation |
| 7 | 27240144 | 27240258 | Cancer-specific | hypermethylation |
| 7 | 27240270 | 27240365 | Cancer-specific | hypermethylation |
| 7 | 27241879 | 27242054 | Cancer-specific | hypermethylation |
| 7 | 27242069 | 27242168 | Cancer-specific | hypermethylation |
| 7 | 27244524 | 27244585 | Cancer-specific | hypermethylation |
| 7 | 27244596 | 27244680 | Cancer-specific | hypermethylation |
| 7 | 27252612 | 27252818 | Cancer-specific | hypermethylation |
| 7 | 27252813 | 27252998 | Cancer-specific | hypermethylation |
| 7 | 27280571 | 27280656 | Cancer-specific | hypermethylation |
| 7 | 27281757 | 27282151 | Cancer-specific | hypermethylation |
| 7 | 27282226 | 27282372 | Cancer-specific | hypermethylation |
| 7 | 27282419 | 27282544 | Cancer-specific | hypermethylation |
| 7 | 28997369 | 28997509 | Cancer-specific | hypermethylation |
| 7 | 28997515 | 28997610 | Cancer-specific | hypermethylation |
| 7 | 28997804 | 28997892 | Cancer-specific | hypermethylation |
| 7 | 28997918 | 28998085 | Cancer-specific | hypermethylation |
| 7 | 28998090 | 28998298 | Cancer-specific | hypermethylation |
| 7 | 30029710 | 30029764 | Cancer-specific | hypermethylation |
| 7 | 30029769 | 30029851 | Cancer-specific | hypermethylation |
| 7 | 3033376 | 3033911 | Cancer-specific | hypomethylation |
| 7 | 30722194 | 30722334 | Cancer-specific | hypermethylation |
| 7 | 30722330 | 30722474 | Cancer-specific | hypermethylation |
| 7 | 3341399 | 3341637 | Cancer-specific | hypermethylation |
| 7 | 3341808 | 3341898 | Cancer-specific | hypermethylation |
| 7 | 3341927 | 3341997 | Cancer-specific | hypermethylation |
| 7 | 35293645 | 35293790 | Cancer-specific | hypermethylation |
| 7 | 35293783 | 35293876 | Cancer-specific | hypermethylation |
| 7 | 38670679 | 38670883 | Cancer-specific | hypermethylation |
| 7 | 38670885 | 38671083 | Cancer-specific | hypermethylation |
| 7 | 39649247 | 39649369 | Cancer-specific | hypermethylation |
| 7 | 39649380 | 39649485 | Cancer-specific | hypermethylation |
| 7 | 4030588 | 4030912 | Cancer-specific | hypomethylation |
| 7 | 43152014 | 43152261 | Cancer-specific | hypermethylation |
| 7 | 43152262 | 43152417 | Cancer-specific | hypermethylation |
| 7 | 44143886 | 44143987 | Cancer-specific | hypermethylation |
| 7 | 44143978 | 44144096 | Cancer-specific | hypermethylation |
| 7 | 44349274 | 44349408 | Cancer-specific | hypermethylation |
| 7 | 44349397 | 44349516 | Cancer-specific | hypermethylation |
| 7 | 49812954 | 49813072 | Cancer-specific | hypermethylation |
| 7 | 49813072 | 49813167 | Cancer-specific | hypermethylation |
| 7 | 49813360 | 49813482 | Cancer-specific | hypermethylation |
| 7 | 49813479 | 49813613 | Cancer-specific | hypermethylation |
| 7 | 49815515 | 49815758 | Cancer-specific | hypermethylation |
| 7 | 4998367 | 4998508 | Cancer-specific | hypermethylation |
| 7 | 4998499 | 4998620 | Cancer-specific | hypermethylation |
| 7 | 50343243 | 50343394 | Cancer-specific | hypermethylation |
| 7 | 54609900 | 54609999 | Cancer-specific | hypermethylation |
| 7 | 64030072 | 64030225 | Cancer-specific | hypermethylation |
| 7 | 64030223 | 64030354 | Cancer-specific | hypermethylation |
| 7 | 65037718 | 65037805 | Cancer-specific | hypermethylation |
| 7 | 79083937 | 79084085 | Cancer-specific | hypermethylation |
| 7 | 80507142 | 80507156 | Both | hypomethylation |
| 7 | 80544472 | 80544699 | Both | hypomethylation |
| 7 | 82072098 | 82072156 | Cancer-specific | hypermethylation |
| 7 | 83813613 | 83813819 | Both | hypomethylation |
| 7 | 86688376 | 86688494 | Cancer-specific | hypermethylation |
| 7 | 87935807 | 87935991 | Cancer-specific | hypermethylation |
| 7 | 87936008 | 87936126 | Cancer-specific | hypermethylation |
| 7 | 87974703 | 87974844 | Cancer-specific | hypermethylation |
| 7 | 88388291 | 88388350 | Cancer-specific | hypermethylation |
| 7 | 89950561 | 89950643 | Cancer-specific | hypermethylation |
| 7 | 89950664 | 89950784 | Cancer-specific | hypermethylation |
| 7 | 93519141 | 93519227 | Both | hypermethylation |
| 7 | 96622035 | 96622150 | Cancer-specific | hypermethylation |
| 7 | 96625546 | 96625713 | Cancer-specific | hypermethylation |
| 7 | 96631465 | 96631714 | Cancer-specific | hypermethylation |
| 7 | 96631714 | 96632129 | Cancer-specific | hypermethylation |
| 7 | 96632739 | 96632784 | Cancer-specific | hypermethylation |
| 7 | 96632798 | 96632857 | Cancer-specific | hypermethylation |
| 7 | 96634706 | 96634787 | Cancer-specific | hypermethylation |
| 7 | 96636489 | 96636787 | Cancer-specific | hypermethylation |
| 7 | 96636786 | 96636985 | Cancer-specific | hypermethylation |
| 7 | 96647769 | 96648148 | Cancer-specific | hypermethylation |
| 7 | 96654331 | 96654561 | Cancer-specific | hypermethylation |
| 7 | 96654565 | 96655051 | Cancer-specific | hypermethylation |
| 7 | 97501305 | 97501459 | Cancer-specific | hypermethylation |
| 7 | 98245894 | 98246013 | Cancer-specific | hypermethylation |
| 7 | 98246006 | 98246121 | Cancer-specific | hypermethylation |
| 7 | 98467258 | 98467528 | Cancer-specific | hypermethylation |
| 7 | 98467536 | 98467629 | Cancer-specific | hypermethylation |
| 7 | 98467618 | 98467697 | Cancer-specific | hypermethylation |
| 7 | 98468040 | 98468123 | Cancer-specific | hypermethylation |
| 7 | 98468153 | 98468289 | Cancer-specific | hypermethylation |
| 7 | 98990500 | 98990566 | Cancer-specific | hypermethylation |
| 7 | 98990619 | 98990753 | Cancer-specific | hypermethylation |
| 7 | 99674857 | 99675037 | Both | hypomethylation |
| 8 | 101661793 | 101661918 | Cancer-specific | hypermethylation |
| 8 | 101661909 | 101662102 | Cancer-specific | hypermethylation |
| 8 | 102504422 | 102504571 | Cancer-specific | hypermethylation |
| 8 | 104153048 | 104153180 | Cancer-specific | hypermethylation |
| 8 | 104153179 | 104153290 | Cancer-specific | hypermethylation |
| 8 | 104511332 | 104511409 | Cancer-specific | hypermethylation |
| 8 | 104512735 | 104512988 | Cancer-specific | hypermethylation |
| 8 | 104513017 | 104513090 | Cancer-specific | hypermethylation |
| 8 | 104513558 | 104513777 | Cancer-specific | hypermethylation |
| 8 | 10753022 | 10753404 | Both | hypomethylation |
| 8 | 109095968 | 109096041 | Cancer-specific | hypermethylation |
| 8 | 11204736 | 11204871 | Cancer-specific | hypermethylation |
| 8 | 11204873 | 11204957 | Cancer-specific | hypermethylation |
| 8 | 11205043 | 11205121 | Cancer-specific | hypermethylation |
| 8 | 114444879 | 114444996 | Cancer-specific | hypermethylation |
| 8 | 11540665 | 11540859 | Cancer-specific | hypermethylation |
| 8 | 11540865 | 11541020 | Cancer-specific | hypermethylation |
| 8 | 11557759 | 11558100 | Cancer-specific | hypermethylation |
| 8 | 11558104 | 11558258 | Cancer-specific | hypermethylation |
| 8 | 11558265 | 11558415 | Cancer-specific | hypermethylation |
| 8 | 11617015 | 11617132 | Tissue-specific | hypermethylation |
| 8 | 11617132 | 11617200 | Both | hypermethylation |
| 8 | 120650959 | 120651076 | Cancer-specific | hypermethylation |
| 8 | 120651183 | 120651405 | Cancer-specific | hypermethylation |
| 8 | 121137759 | 121137820 | Cancer-specific | hypermethylation |
| 8 | 121137838 | 121137966 | Cancer-specific | hypermethylation |
| 8 | 124332854 | 124332868 | Both | hypomethylation |
| 8 | 1248935 | 1249274 | Both | hypomethylation |
| 8 | 126403691 | 126403739 | Both | hypomethylation |
| 8 | 126403900 | 126403966 | Both | hypomethylation |
| 8 | 127489100 | 127489472 | Cancer-specific | hypomethylation |
| 8 | 127568794 | 127568919 | Cancer-specific | hypermethylation |
| 8 | 127568915 | 127569117 | Cancer-specific | hypermethylation |
| 8 | 1310962 | 1311022 | Cancer-specific | hypomethylation |
| 8 | 1311056 | 1311185 | Both | hypomethylation |
| 8 | 141468583 | 141468750 | Cancer-specific | hypermethylation |
| 8 | 144511284 | 144511338 | Cancer-specific | hypermethylation |
| 8 | 144650643 | 144650799 | Both | hypermethylation |
| 8 | 144650795 | 144650844 | Both | hypermethylation |
| 8 | 1448297 | 1448528 | Both | hypomethylation |
| 8 | 1449394 | 1449745 | Both | hypomethylation |
| 8 | 145106175 | 145106323 | Both | hypermethylation |
| 8 | 145106319 | 145106423 | Both | hypermethylation |
| 8 | 145925590 | 145925901 | Cancer-specific | hypermethylation |
| 8 | 145925917 | 145926006 | Cancer-specific | hypermethylation |
| 8 | 145926013 | 145926200 | Cancer-specific | hypermethylation |
| 8 | 19459973 | 19460072 | Tissue-specific | hypermethylation |
| 8 | 19460072 | 19460250 | Tissue-specific | hypermethylation |
| 8 | 23583980 | 23584049 | Cancer-specific | hypermethylation |
| 8 | 2363659 | 2363928 | Both | hypomethylation |
| 8 | 25902162 | 25902262 | Cancer-specific | hypermethylation |
| 8 | 25905356 | 25905485 | Cancer-specific | hypermethylation |
| 8 | 25905502 | 25905603 | Cancer-specific | hypermethylation |
| 8 | 26305917 | 26306002 | Cancer-specific | hypermethylation |
| 8 | 26305998 | 26306084 | Cancer-specific | hypermethylation |
| 8 | 26437360 | 26437476 | Both | hypomethylation |
| 8 | 37823173 | 37823279 | Cancer-specific | hypermethylation |
| 8 | 37823274 | 37823416 | Cancer-specific | hypermethylation |
| 8 | 38965071 | 38965170 | Cancer-specific | hypermethylation |
| 8 | 38965162 | 38965308 | Cancer-specific | hypermethylation |
| 8 | 41504071 | 41504204 | Cancer-specific | hypermethylation |
| 8 | 49293275 | 49293386 | Cancer-specific | hypermethylation |
| 8 | 49293375 | 49293464 | Cancer-specific | hypermethylation |
| 8 | 49293468 | 49293504 | Cancer-specific | hypermethylation |
| 8 | 53852094 | 53852218 | Cancer-specific | hypermethylation |
| 8 | 53852224 | 53852305 | Cancer-specific | hypermethylation |
| 8 | 54164204 | 54164430 | Cancer-specific | hypermethylation |
| 8 | 54790365 | 54790476 | Cancer-specific | hypermethylation |
| 8 | 55366687 | 55366802 | Cancer-specific | hypermethylation |
| 8 | 55379898 | 55380015 | Cancer-specific | hypermethylation |
| 8 | 55380013 | 55380135 | Cancer-specific | hypermethylation |
| 8 | 57069784 | 57069926 | Cancer-specific | hypermethylation |
| 8 | 57069924 | 57070098 | Cancer-specific | hypermethylation |
| 8 | 57358340 | 57358460 | Cancer-specific | hypermethylation |
| 8 | 57358457 | 57358580 | Cancer-specific | hypermethylation |
| 8 | 61822421 | 61822545 | Cancer-specific | hypermethylation |
| 8 | 62200373 | 62200470 | Cancer-specific | hypermethylation |
| 8 | 67873360 | 67873713 | Cancer-specific | hypermethylation |
| 8 | 67873726 | 67873925 | Cancer-specific | hypermethylation |
| 8 | 67874052 | 67874172 | Cancer-specific | hypermethylation |
| 8 | 67874160 | 67874344 | Cancer-specific | hypermethylation |
| 8 | 67874916 | 67874995 | Cancer-specific | hypermethylation |
| 8 | 67875329 | 67875448 | Cancer-specific | hypermethylation |
| 8 | 67875441 | 67875560 | Cancer-specific | hypermethylation |
| 8 | 689271 | 689399 | Cancer-specific | hypermethylation |
| 8 | 69244389 | 69244517 | Cancer-specific | hypermethylation |
| 8 | 70946932 | 70947023 | Cancer-specific | hypermethylation |
| 8 | 70947025 | 70947171 | Cancer-specific | hypermethylation |
| 8 | 70982158 | 70982369 | Cancer-specific | hypermethylation |
| 8 | 70983480 | 70983614 | Cancer-specific | hypermethylation |
| 8 | 70984143 | 70984362 | Cancer-specific | hypermethylation |
| 8 | 70984357 | 70984545 | Cancer-specific | hypermethylation |
| 8 | 72468713 | 72468888 | Cancer-specific | hypermethylation |
| 8 | 72756119 | 72756264 | Cancer-specific | hypermethylation |
| 8 | 72756269 | 72756445 | Cancer-specific | hypermethylation |
| 8 | 72756703 | 72756832 | Cancer-specific | hypermethylation |
| 8 | 77585120 | 77585229 | Cancer-specific | hypermethylation |
| 8 | 77585228 | 77585325 | Cancer-specific | hypermethylation |
| 8 | 82193355 | 82193458 | Cancer-specific | hypermethylation |
| 8 | 82193453 | 82193566 | Cancer-specific | hypermethylation |
| 8 | 85096924 | 85097066 | Cancer-specific | hypermethylation |
| 8 | 85097056 | 85097253 | Cancer-specific | hypermethylation |
| 8 | 91803988 | 91804095 | Cancer-specific | hypermethylation |
| 8 | 93115197 | 93115423 | Cancer-specific | hypermethylation |
| 8 | 95168223 | 95168497 | Both | hypomethylation |
| 8 | 95246418 | 95246521 | Cancer-specific | hypermethylation |
| 8 | 95246517 | 95246632 | Cancer-specific | hypermethylation |
| 8 | 95651251 | 95651391 | Cancer-specific | hypermethylation |
| 8 | 95651426 | 95651494 | Cancer-specific | hypermethylation |
| 8 | 98290029 | 98290340 | Cancer-specific | hypermethylation |
| 8 | 98290352 | 98290581 | Both | hypermethylation |
| 8 | 99951300 | 99951427 | Cancer-specific | hypermethylation |
| 8 | 99951442 | 99951505 | Both | hypermethylation |
| 8 | 99952472 | 99952563 | Cancer-specific | hypermethylation |
| 8 | 99952556 | 99952686 | Cancer-specific | hypermethylation |
| 8 | 99986060 | 99986263 | Cancer-specific | hypermethylation |
| 8 | 99986259 | 99986436 | Cancer-specific | hypermethylation |
| 9 | 1051961 | 1052067 | Cancer-specific | hypermethylation |
| 9 | 110228144 | 110228278 | Cancer-specific | hypermethylation |
| 9 | 110228268 | 110228389 | Cancer-specific | hypermethylation |
| 9 | 116355880 | 116355985 | Both | hypermethylation |
| 9 | 116356051 | 116356115 | Cancer-specific | hypermethylation |
| 9 | 120176542 | 120176637 | Cancer-specific | hypermethylation |
| 9 | 122131928 | 122132112 | Cancer-specific | hypermethylation |
| 9 | 124384783 | 124384930 | Cancer-specific | hypomethylation |
| 9 | 124888943 | 124889064 | Cancer-specific | hypermethylation |
| 9 | 124889052 | 124889146 | Cancer-specific | hypermethylation |
| 9 | 124981552 | 124981618 | Cancer-specific | hypermethylation |
| 9 | 124982080 | 124982173 | Cancer-specific | hypermethylation |
| 9 | 124982170 | 124982316 | Cancer-specific | hypermethylation |
| 9 | 126777566 | 126777690 | Cancer-specific | hypermethylation |
| 9 | 126777740 | 126777797 | Cancer-specific | hypermethylation |
| 9 | 129276464 | 129276635 | Cancer-specific | hypermethylation |
| 9 | 133534836 | 133534970 | Cancer-specific | hypermethylation |
| 9 | 133534971 | 133535080 | Cancer-specific | hypermethylation |
| 9 | 139085324 | 139085555 | Cancer-specific | hypermethylation |
| 9 | 139438222 | 139438447 | Both | hypermethylation |
| 9 | 139438451 | 139438557 | Both | hypermethylation |
| 9 | 139939811 | 139939869 | Cancer-specific | hypermethylation |
| 9 | 139939886 | 139939933 | Cancer-specific | hypermethylation |
| 9 | 140033387 | 140033491 | Cancer-specific | hypermethylation |
| 9 | 140051091 | 140051212 | Cancer-specific | hypermethylation |
| 9 | 140051206 | 140051329 | Cancer-specific | hypermethylation |
| 9 | 140780544 | 140780664 | Cancer-specific | hypomethylation |
| 9 | 140780692 | 140780855 | Both | hypomethylation |
| 9 | 140944782 | 140944843 | Both | hypomethylation |
| 9 | 21968118 | 21968311 | Cancer-specific | hypermethylation |
| 9 | 21968313 | 21968494 | Cancer-specific | hypermethylation |
| 9 | 21974580 | 21974726 | Cancer-specific | hypermethylation |
| 9 | 21974718 | 21974825 | Cancer-specific | hypermethylation |
| 9 | 21974951 | 21975038 | Cancer-specific | hypermethylation |
| 9 | 21975045 | 21975198 | Cancer-specific | hypermethylation |
| 9 | 23820998 | 23821106 | Cancer-specific | hypermethylation |
| 9 | 34624495 | 34624598 | Both | hypermethylation |
| 9 | 34624603 | 34624657 | Both | hypermethylation |
| 9 | 37030243 | 37030317 | Cancer-specific | hypermethylation |
| 9 | 37036416 | 37036531 | Cancer-specific | hypermethylation |
| 9 | 37036526 | 37036607 | Cancer-specific | hypermethylation |
| 9 | 37037941 | 37038081 | Cancer-specific | hypermethylation |
| 9 | 37038093 | 37038168 | Cancer-specific | hypermethylation |
| 9 | 87283344 | 87283443 | Cancer-specific | hypermethylation |
| 9 | 89560769 | 89560888 | Cancer-specific | hypermethylation |
| 9 | 89560880 | 89561005 | Cancer-specific | hypermethylation |
| 9 | 91849625 | 91849722 | Cancer-specific | hypermethylation |
| 9 | 91849721 | 91849832 | Cancer-specific | hypermethylation |
| 9 | 96108592 | 96108900 | Cancer-specific | hypermethylation |
| 9 | 96714171 | 96714320 | Cancer-specific | hypermethylation |
| 9 | 96714353 | 96714420 | Cancer-specific | hypermethylation |
