# Supplementary material for: Cell-free DNA methylation markers for differential diagnosis of hepatocellular carcinoma
Source: BMC Med. 2022 Jan 14;20:8. doi: 10.1186/s12916-021-02201-3 (PMC8759185; doi:10.1186/s12916-021-02201-3)
Supplement: Supplementary file 2 — Additional file 2: Table S2. Table S2. Performance of tissue-derived markers in tissue-samples. [file 12916_2021_2201_MOESM2_ESM.docx]

Table S2: Performance of tissue-derived markers in tissue-samples

|  | | Predicted | | | |
| --- | --- | --- | --- | --- | --- |
|  | **Total** | **Negative** | **Positive** | **Sensitivity (%)** | **Specificity (%)** |
| *HCC* | 31 | 2 | 29 | 94 |  |
| *Cirrhosis* | 17 | 17 | 0 |  | 100 |
| *Healthy Individuals* | 15 | 15 | 0 |  | 100 |
