# Supplementary material for: Cell-free DNA methylation markers for differential diagnosis of hepatocellular carcinoma
Source: BMC Med. 2022 Jan 14;20:8. doi: 10.1186/s12916-021-02201-3 (PMC8759185; doi:10.1186/s12916-021-02201-3)
Supplement: Supplementary file 3 — Additional file 3: Figures S1-S11. Fig. S1 The input DNA amount health, benign, and tumor tissue samples in sequencing analysis. Fig. S2 cfDNA methylation analysis of HCC diagnosis. Fig. S3 GO and KEGG pathway analysis of benign and cancer signals. Fig. S4 Unsupervised hierarchical clustering of tissue-derived 2321 methylation markers selected for HCC diagnosis in the tissue samples and plasma samples. Fig. S5 The specificity and sensitivity of MH model distinguishing tumor from healthy individuals in the training and the validation cohort. Fig. S6 The specificity and sensitivity of BH model distinguishing tumor from healthy individuals in the training and the validation cohort. Fig. S7 The comparison of non-tissue tissue-specific markers and tissue-specific markers in BH model contribution. Fig. S8 The specificity and sensitivity of MB model distinguishing tumor from healthy individuals in the training and the validation cohort. Fig. S9 The ROC of the HCC screening model (malignant score) for HCC diagnosis in the training and the validation cohort. Fig. S10 The correlation of malignant score and benign score with cause of cirrhosis (HCV or non-HCV). Fig. S11 Clinical characteristics of misclassified cirrhotic samples. [file 12916_2021_2201_MOESM3_ESM.docx]

Supplemental Figures

Figure S1: The input DNA amount health, benign, and tumor tissue samples in sequencing analysis.


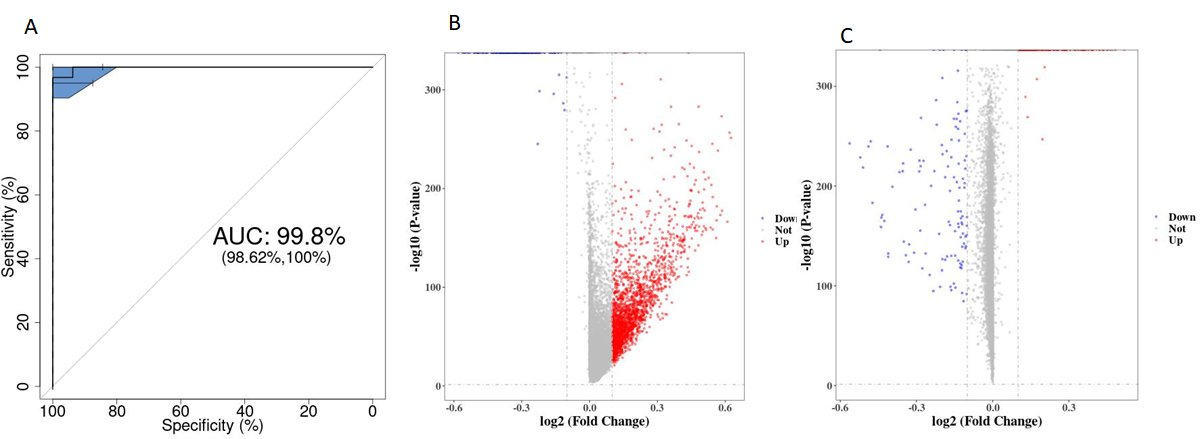


Figure S2: cfDNA methylation analysis of HCC diagnosis. A. ROC of the diagnostic prediction model with methylation markers in tissue samples; B. Of 2,293 tumor-specific markers, 2,082 were hypermethylated and 211 were hypomethylated; C. of 279 tissue-specific markers, 158 were hypermethylated and 121 were hypomethylated.


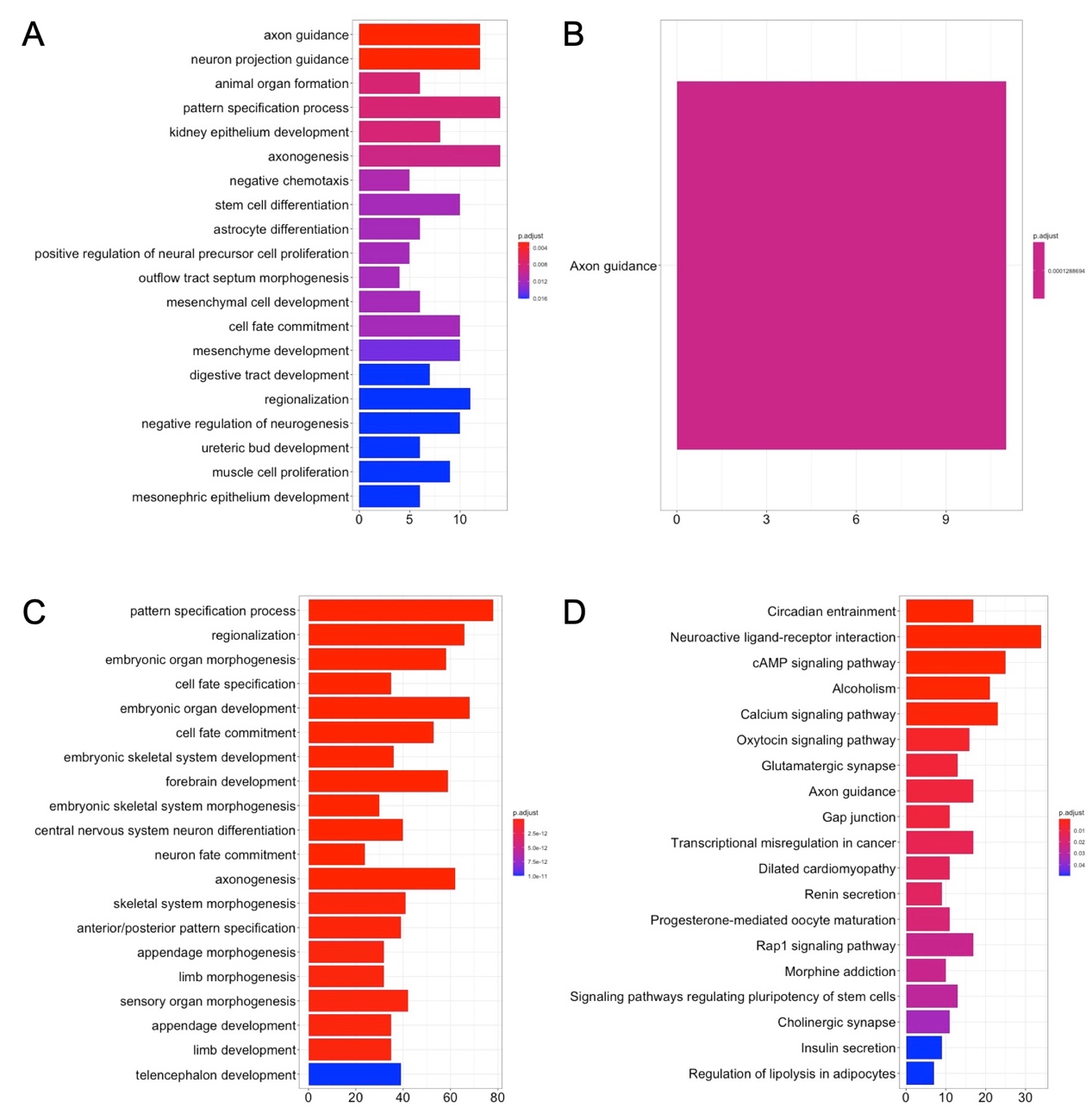


Figure S3: GO and KEGG pathway analysis of benign and cancer signals. A. GO analysis of benign signals; B KEGG pathway analysis of benign signals; C A. GO analysis of cancer signals; D KEGG pathway analysis of cancer signals.


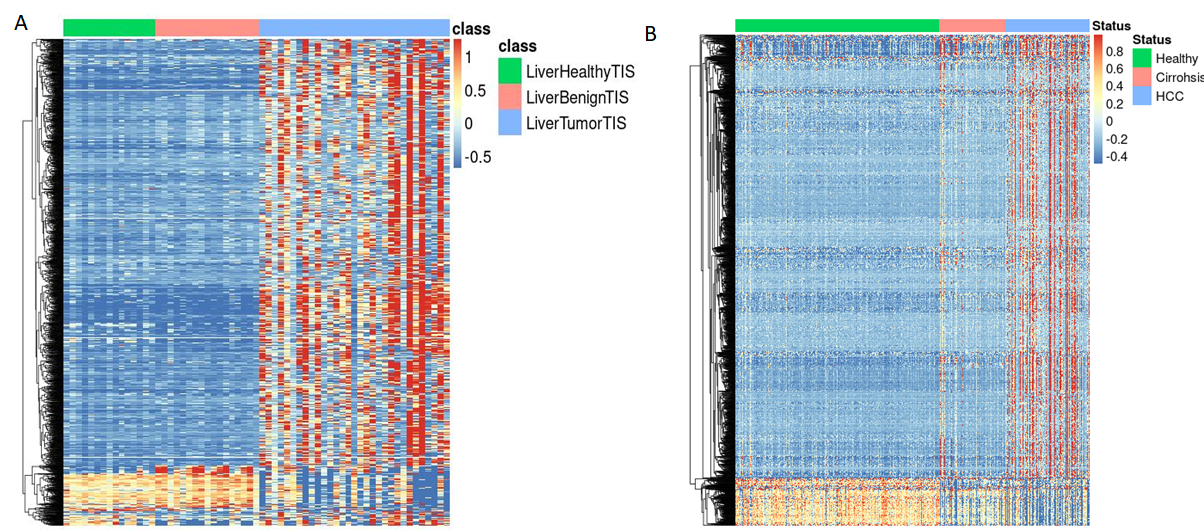


Figure S4: Unsupervised hierarchical clustering of tissue-derived 2321 methylation markers selected for HCC diagnosis in the tissue samples (A) and plasma samples (B).


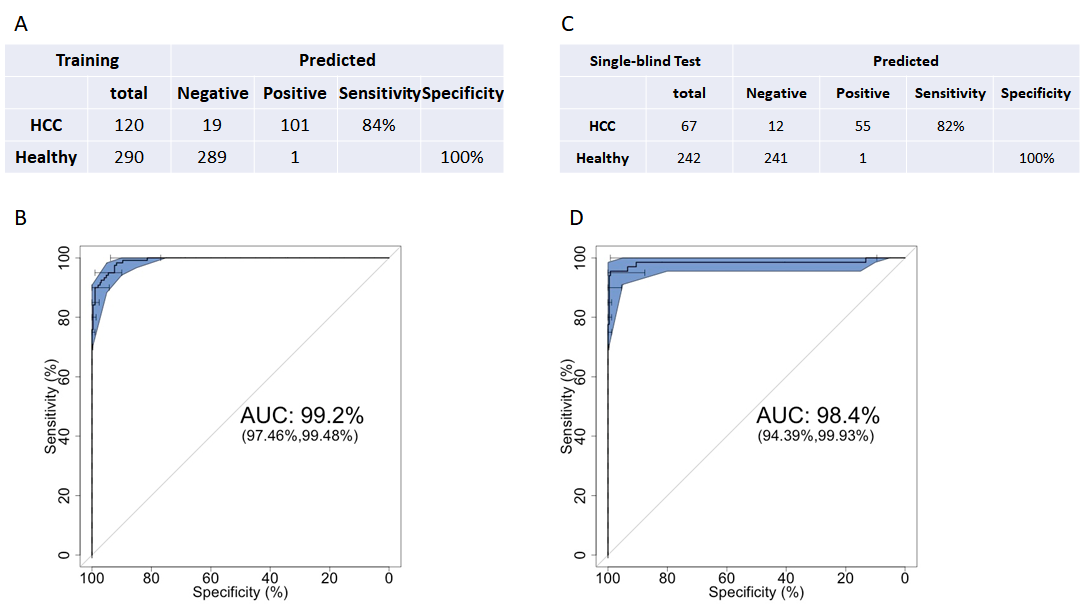


Figure S5: The specificity and sensitivity of malignant vs. healthy (MH) model distinguishing tumor from healthy individuals in the training cohort (A, B) and the validation cohort (C, D).


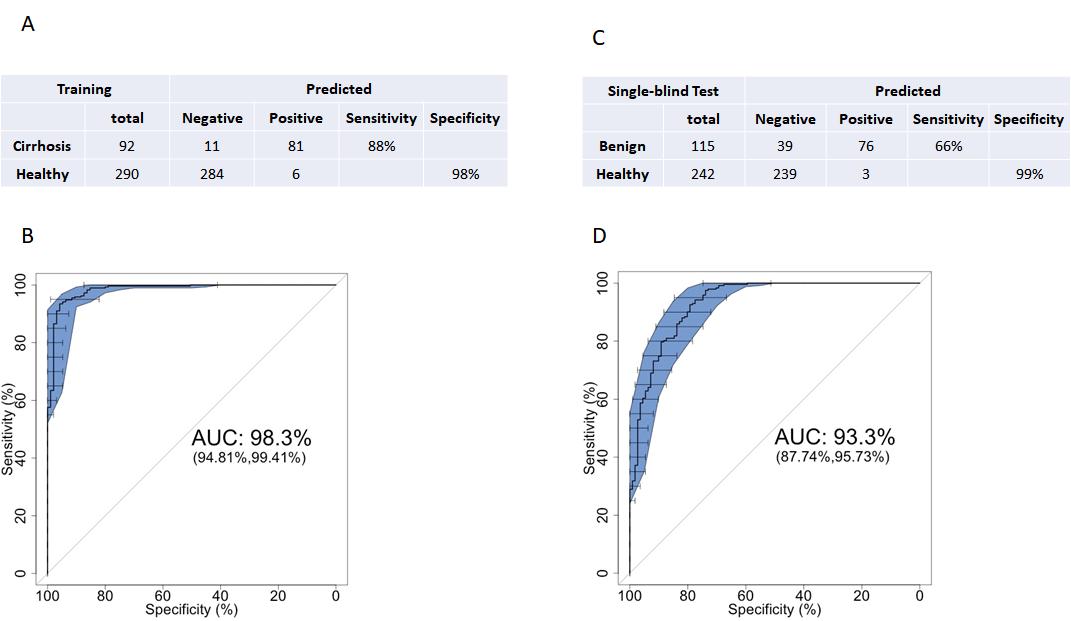


Figure S6: The specificity and sensitivity of benign vs healthy model (BH model) distinguishing LC samples from healthy individuals in the training cohort (A, B) and the validation cohort (C, D).


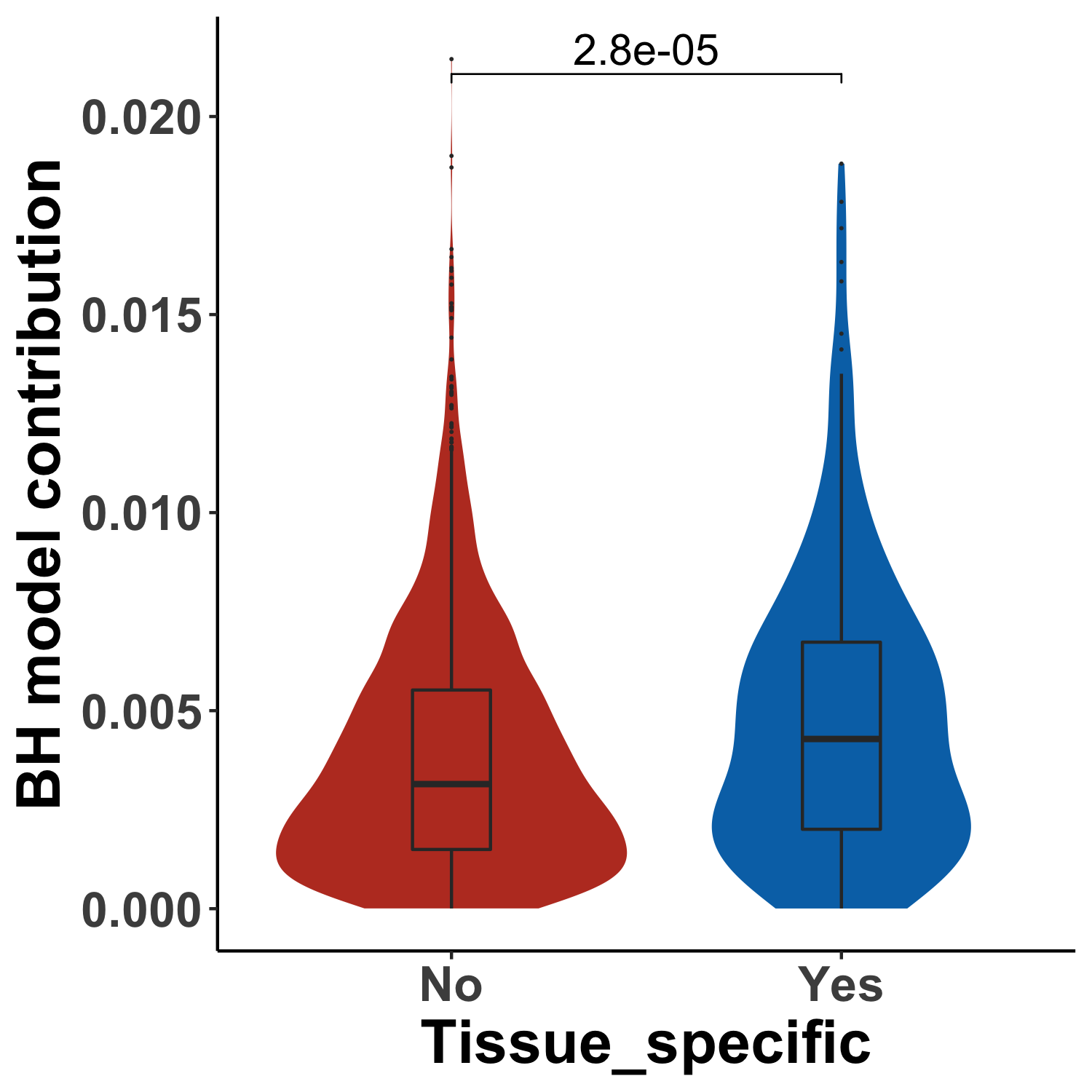


Figure S7: The comparison of non-tissue tissue-specific markers and tissue-specific markers in BH model contribution.


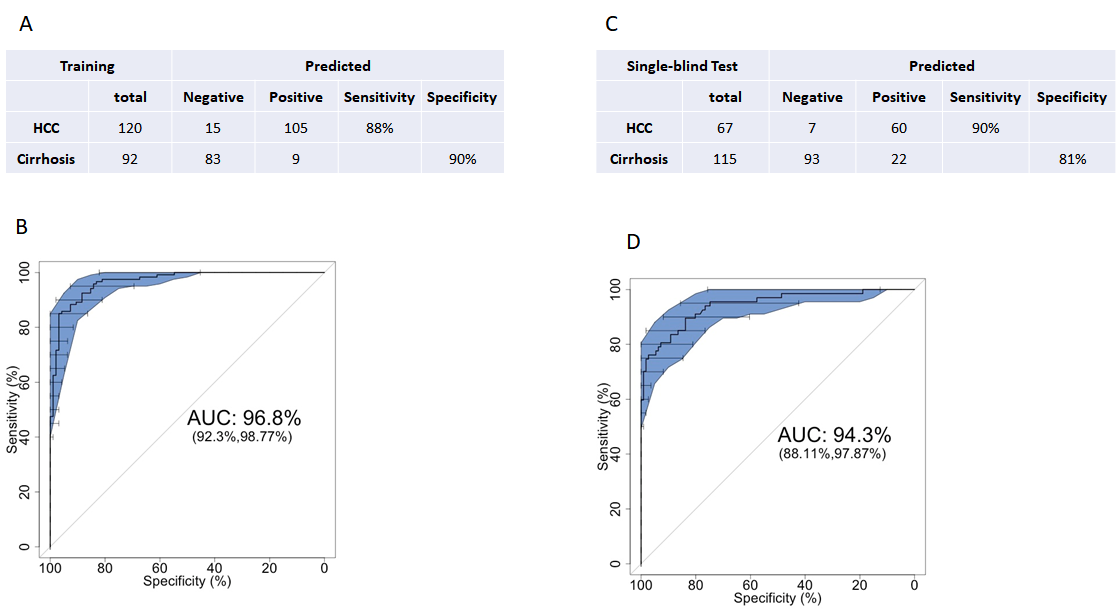


Figure S8: The specificity and sensitivity of malignant vs benign model (MB model) distinguishing tumor from LC controls in the training cohort (A, B) and the validation cohort (C, D).


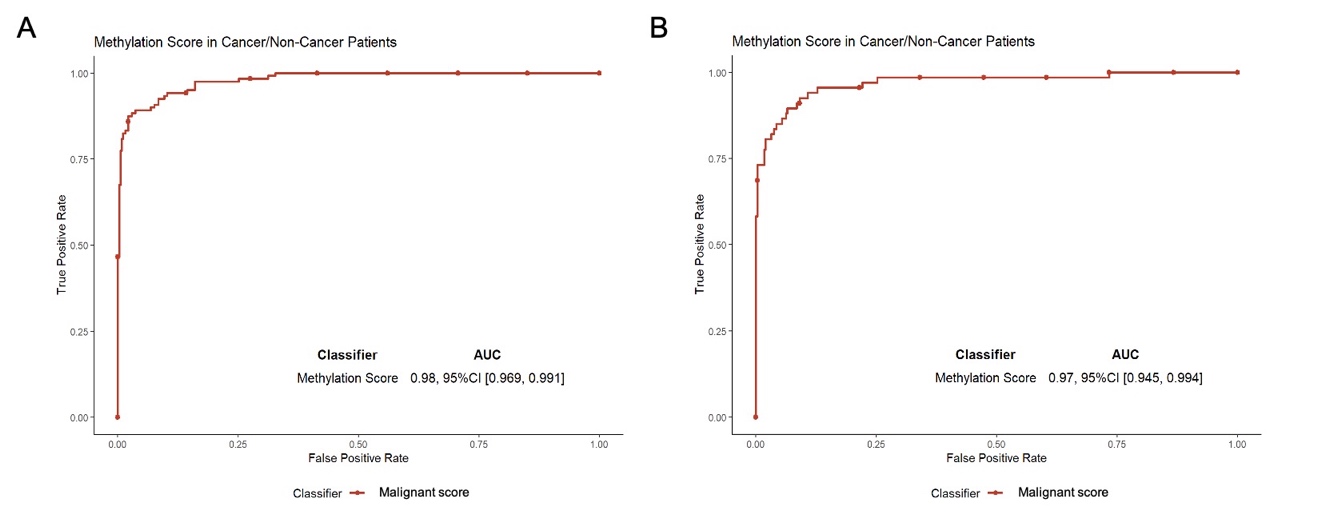


Figure S9: The ROC of the HCC screening model (malignant score) for HCC diagnosis in the training (A) and the validation cohort (B)


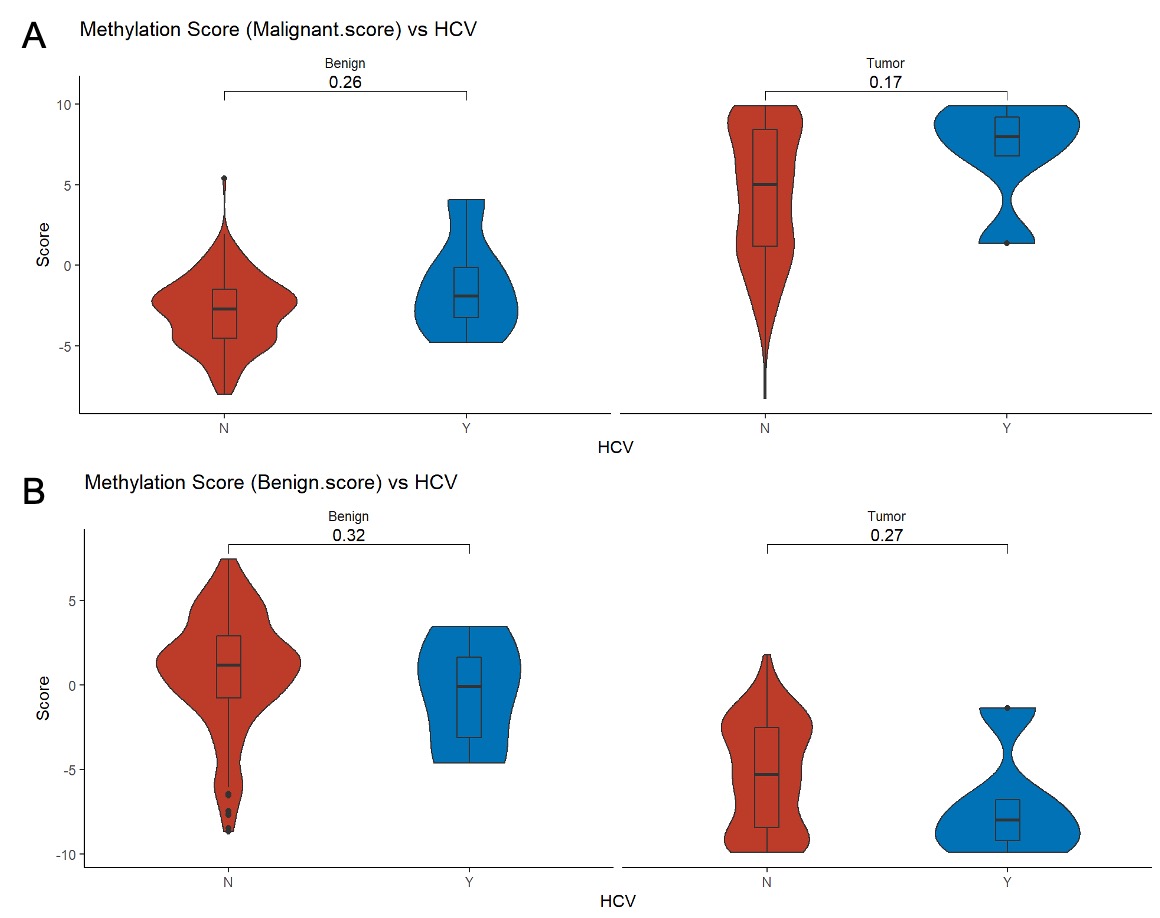


Figure S10: The correlation of malignant score(A) and benign score(B) with cause of cirrhosis (HCV or non-HCV)


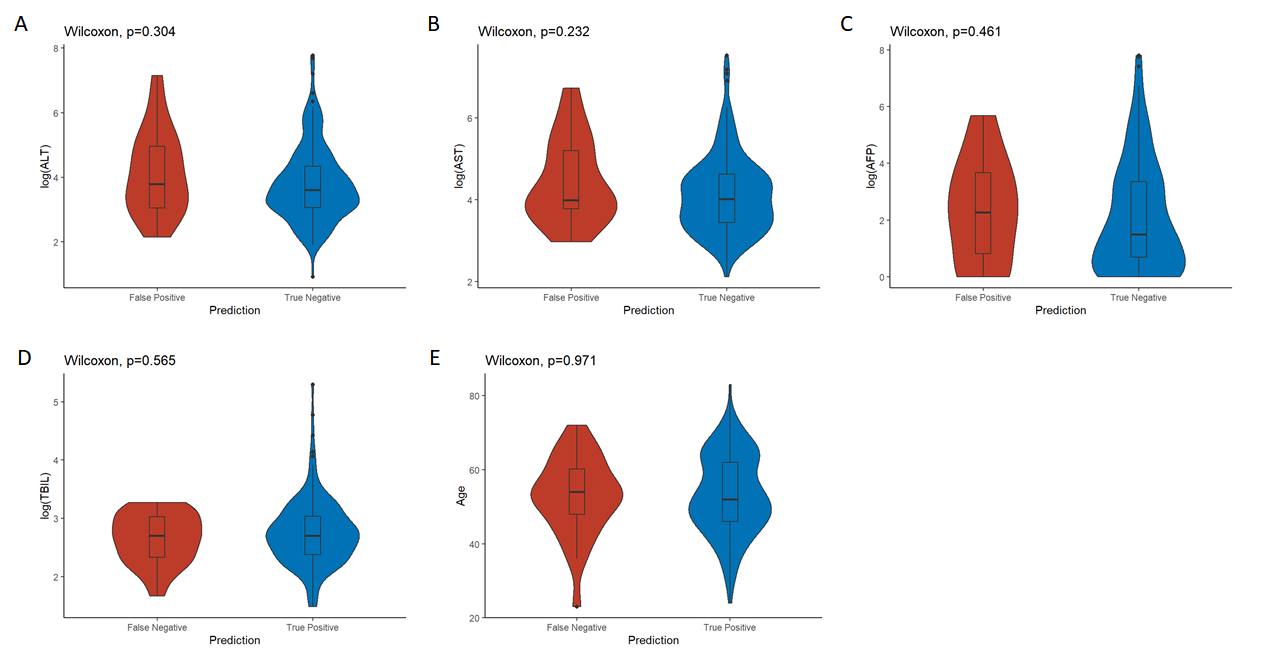


Figure S11: Clinical characteristics of misclassified cirrhotic samples. (A-C) ALT, AST, AFP levels were comparable between 19 cirrhotic samples; (D-E) Bilirubin level, age levels were comparable between 28 HCC samples.
